# Supplementary material for: Pollution index of waterfowl farm assessment and prediction based on temporal convoluted network
Source: PLoS One. 2021 Jul 23;16(7):e0254179. doi: 10.1371/journal.pone.0254179 (PMC8301615; doi:10.1371/journal.pone.0254179)
Supplement: Attachment — Submitted filename: PONE-D-21-03161_R2_reviewer.pdf [file pone.0254179.s004.pdf]

PLOS ONE

Pollution index of waterfowl farm assessment and prediction based on temporal convoluted network

--Manuscript Draft--

**Manuscript Number:**	PONE-D-21-03161R2
**Article Type:**	Research Article
**Full Title:**	Pollution index of waterfowl farm assessment and prediction based on temporal convoluted network
**Short Title:**	Pollution index of waterfowl farm assessment and prediction based on temporal convoluted network
**Corresponding Author:**	Shuangyin Liu<br>Zhongkai University of Agriculture and Engineering<br>Guangzhou, CHINA CHINA
**Keywords:**	Agricultural informatization; environmental quality assessment; temporal convoluted network; regression prediction
**Abstract:**	The pollutiEnvironment quality is a major factor that directly impacts waterfowl productivity. Accurate prediction of PI is the key to improving environmental management and pollution control. This study applied a new neural network model called temporal convolutional network and a denoising algorithm called wavelet transform (WT) for predicting future 12, 24, and 48-hours PI values at a waterfowl farm in Shanwei, China. The TCN model performance was compared with that of the recurrent architectures with the same capacity, long-short time memory neural network (LSTM), and gated recurrent unit (GRU). Denoised environmental data including ammonia, temperature, relative humidity, carbon dioxide (Co 2), and total suspended particles (TSP) were used for constructing the forecasting model. The simulation results showed that the TCN model in general produced a more precise PI prediction and provided the highest prediction accuracy for all phases (MAE = 0.0842, 0.0859, 0.1115, RMSE = 0.0154, 0.0167, 0.0273, R2 = 0.9789, 0.9791, 0.9635). The PI assessment prediction model based on TCN exhibited the best prediction accuracy, and general performance compared with other parallel forecasting models and is a suitable and useful tool for predicting PI in waterfowl farms.
**Order of Authors:**	jiande Huang
	Shuangyin Liu
	Shahbaz Gul Hassan
	Longqin Xu
**Response to Reviewers:**	Response to Journal:<br><br>Reviewer #1: While I commend the authors for working diligently on the suggested comments by both reviewers, the manuscript is still not in a publishable state. I still maintain that the work is interesting and prudent, but the authors have not fully addressed some of the major comments brought up previously. First being the English is still not at a level of an academic paper and really needs major edits which I cannot put forth the time to do.<br><br>Answer: Thank you very much for your prestigious comments. This paper was edited for proper English language, grammar, punctuation, spelling, and overall style by one or more of the highly qualified native English speaking editors at AJE.<br><br>Second, the paper is still very methods focus despite the fact that both reviewers pointed out that there needs to be more on the "why" do the study. What's the motivation? How can it help farmers. The authors added a few lines here and there but it is still not compelling.<br><br>Answer: We have added more references to this study. It includes why we need to monitor and control the poultry house environment, what methods have been used to

Powered by Editorial Manager® and ProduXion Manager® from Aries Systems Corporation

do that thing by others, how can they help the farmer and why we need the new method.

Labor shortages and increasing biosecurity practices will make it more difficult for producers to monitor and manage the production, health, and welfare status of all of their birds. Employing modern poultry management technology is necessary to increase production [6]. An example of how modern management technology can be used to monitor and control the poultry house environment is exemplified by humidity regulation via ventilation rate changes mediated by relative humidity sensors, as relative humidity is one of the more important environmental aspects of a poultry house [7]. In addition, more advanced systems are being researched. Bustamante et al. used a multisensor system to effectively assess barn ventilation system function by tracking temperature, air velocity and differential pressure in broiler houses [8]. Hanif et al. proposed an internet of things technology-based protection and monitoring of the environment of a poultry house to monitor the environment-related parameters such as air temperature, air humidity, CO2 level of concentration and ammonia concentration., which has been implemented successfully, leading to a safe environment and profit for the poultry industry [9]. The techniques mentioned above are both solutions for real-time environmental monitoring and control; however, relying on hardware monitoring in real time cannot capture the trend of environmental changes [10], and it is easy to miss the best time for adjustment, which leads to waterfowl health damage and property loss.

Cultivation environment forecasting has been studied for many years and has made some achievements in aquaculture and livestock breeding [11–14]. The technique estimates or predicts the future changes in target variables that cannot be obtained directly. For example, Jackman et al. generated a prediction model by using sensor inputs of relative humidity, carbon dioxide, temperature, and ammonia for environmental parameter and crop yield prediction [15]. In waterfowl production, a system such as this would allow for actions to be taken sooner by farmers if the environment is projected to be bad. However, few studies have applied prediction in waterfowl breeding. Therefore, it is necessary to apply environmental prediction technology to waterfowl production to fill this gap.

1. While the first table highlights the data chosen for the PI. The text does not go into much detail about why these were chosen, and what are the acceptable limits. I still do not see a mention of other similar studies on waterfowl or poultry in the introduction.

Answer: We have added more references to detail describe why data were chosen, and what are the acceptable limits.

Labor shortages and increasing biosecurity practices will make it more difficult for producers to monitor and manage the production, health, and welfare status of all of their birds. Employing modern poultry management technology is necessary to increase production [6]. An example of how modern management technology can be used to monitor and control the poultry house environment is exemplified by humidity regulation via ventilation rate changes mediated by relative humidity sensors, as relative humidity is one of the more important environmental aspects of a poultry house [7]. In addition, more advanced systems are being researched. Bustamante et al. used a multisensor system to effectively assess barn ventilation system function by tracking temperature, air velocity and differential pressure in broiler houses [8]. Hanif et al. proposed an internet of things technology-based protection and monitoring of the environment of a poultry house to monitor the environment-related parameters such as air temperature, air humidity, CO2 level of concentration and ammonia concentration., which has been implemented successfully, leading to a safe environment and profit for the poultry industry [9]. The techniques mentioned above are both solutions for real-time environmental monitoring and control; however, relying on hardware monitoring in real time cannot capture the trend of environmental changes [10], and it is easy to miss the best time for adjustment, which leads to waterfowl health damage and property loss.

Cultivation environment forecasting has been studied for many years and has made some achievements in aquaculture and livestock breeding [11–14]. The technique estimates or predicts the future changes in target variables that cannot be obtained directly. For example, Jackman et al. generated a prediction model by using sensor inputs of relative humidity, carbon dioxide, temperature, and ammonia for

Powered by Editorial Manager® and ProduXion Manager® from Aries Systems Corporation

environmental parameter and crop yield prediction [15]. In waterfowl production, a system such as this would allow for actions to be taken sooner by farmers if the environment is projected to be bad. However, few studies have applied prediction in waterfowl breeding. Therefore, it is necessary to apply environmental prediction technology to waterfowl production to fill this gap.

According to the importance of the environment and expert research, we selected 5 environmental factors, as shown in Table 1. Among them, ammonia is a toxic gas and the greatest concern of environmental pollution in waterfowl production, adversely affecting the ecosystem, environment, and health of birds and people. Less than 10 ppm is the ideal limit [42]. Relative humidity can impact bird health, and high relative humidity may worsen broiler geese performance; the ideal value is 70% [43]. Heat stress is a major concern in waterfowl production; high and low temperatures will reduce the growth performance and survivability of waterfowl, and temperatures less than 27°C and more than 10°C are ideal [44]. TSP is from the birds themselves as well as from the feed, litter, and building materials and may serve as a pathogen disseminator and bring about lung damage; less than 10 mg/m3 is the ideal limit [42]. Additionally, the levels of relative humidity, ammonia, carbon dioxide, temperature and TSP are known to be correlated with each other [6]. These factors are important for waterfowl production and can be monitored and optimized.
2. There is now way too much detail in the methods. I'm not sure you need every detail and plot for each AI technique in your paper unless this is the first time they are introduced. You should provide a brief summary of each and then if you want to include this detail move to SI.

Answer: We have simplify introduction of some methods.

Wavelet transform
Continuous wavelet transforms for a given waterfowl environment signal s(k) can be described as follows:
(3)
where denotes the complex conjugate, is the wavelet function, a is the time scale dilation and b is the time translation. By controlling the values of parameters a and b, signal time-frequency positioning can be achieved. The wavelet transform can decompose signals into multiple resolutions. However, the symbols in realization communication are discrete data, so the continuous wavelet transform needs to be discretized. Suppose , when :
(4)
The discrete waterfowl environment signal s(n) can be transformed as:
(5)
The Mallat algorithm can quickly calculate the orthogonal wavelet transform coefficients and realize signal decomposition and reconstruction [47]. The approximation coefficients and detail coefficients can be recurrently related by the Mallat algorithm as follows:
(6)
(7)
where h0 and h1 are the high-pass filter and low-pass filter, respectively. The reconstruction is the inverse process of decomposition:
(8)
The wavelet transform can decompose noise signals into different signal channels at various levels of resolution. In general, the useful signals are distributed in the high-level resolution channel, and the noise signal is likely to be distributed in the lower-level channel. Therefore, the noise of the original data can be reduced as much as possible based on a reasonable threshold. Therefore, the wavelet denoised algorithm can be described as follows:
(1)Perform wavelet transform on the original signal s(k) to obtain wavelet coefficients of various scales.
(2)Determine the threshold and perform threshold denoising on different wavelet coefficients to obtain estimated wavelet coefficients.
(3)Conduct wavelet reconstruction by equation 11 to obtain the denoise signal .
Support vector machine
SVM has good generalization ability in solving nonlinear, small sample, and high-dimensional pattern recognition, and the optimal solution obtained is global, which solves the local optimal problem that cannot be avoided in other algorithms. The

Powered by Editorial Manager® and ProduXion Manager® from Aries Systems Corporation

prediction process of the SVM includes support vector determination, kernel function selection, kernel parameter determination, and solution. The mathematical expression as follow:

(9)

Where SVS is the number of support vectors, αi is the Lagrangian coefficient of each training sample, yi (-1 or 1) is the vector label, K (xi, x) is the selected kernel function, and b0 is the bias. The kernel function replaces the linear quantity in the traditional linear equation and maps the data to high-dimensional space processing. After selected the kernel function, the samples are trained to establish the SVM model and the prediction result Nt is finally obtained.

Long short-term memory neural network

The LSTM neural network is a special kind of recurrent neural network. It was first proposed by Ho-chreiter and Schmidhuber [49]. Its appearance effectively solved the gradient explosion problem of traditional recurrent neural networks. At the same time, the LSTM neural network has long-term memory to handle long-term sequence data. The LSTM cell structure consists of forget gate, input gate, output gate and cell state. LSTM structure is shown in Fig 2 At the time t, there are three input parameters of the LSTM network: input value xt, at time t, output value ht-1 and cell state Ct-1 at t-1. There are two output parameters of the LSTM network: output value ht and cell state Ct at time t. Through the activation function σ LSTM realizes the control of the three gates so as to realize the retention and forgetting of historical information.

Fig.2. The structure of LSTM

Gated recurrent unit

A gated recurrent unit (GRU) is a type of recurrent neural network (RNN). Similar to a long short-term memory neural network (LSTM), it is also proposed to solve long-term and gradient backpropagation problems. LSTM and GRU have similar performances, but compared with LSTM, GRU is computationally cheaper. The structure of GRU is shown in Fig 3 GRU can be descried as follows:

Firstly, two output gateds, r (reset gated) and z (update gated), were obtained by the input X(t) at t time and the output h(t − 1) at t − 1 time.

(10)

(11)

Where CJ is a sigmoid function. Parameter h' selective re- members X(t). Trough the reset gated r we can obtain h’ as follows:

(12)

Finally, the output y(t) and hidden state h(t) at t time can be calculated as follows:

(13)

(14)

Where [ ] denoted the connection of two vectors and * denoted the product of matrices.

Fig.3. The structure of GRU

3. Conclusions and future research again focus way too much on methods and not on the "why".

Answer: We have modified the conclusions.

To further promote waterfowl house environment monitoring and controlling technology, reduce labor and increase the production effect, this study analyses the shortcomings of existing methods and introduces a new way to guide waterfowl house environment management by learning from other fields.

The new method investigates the application of denoised WT and the performance of three neural network models and two mechanical learning models in predicting the pollution index of the waterfowl house environment using environmental quality parameters at different intervals. The results indicate that the TCN model has the best performance in predicting PI. The GRU model has similar performance but lower performance when the prediction time interval changed, and the LSTM model performed the worst among the three models, although it still provided fairly accurate PI predictions.

The models presented in this paper, in particular the TCN model, could provide accurate and long-interval PI predictions of waterfowl house environments and monitor them in real time. The simulation results show that this method can be applied in waterfowl house environment prediction. The future trend of the environment can be estimated and predicted compared with traditional real-time monitoring technology,

	which can allow better waterfowl house environment management practices, better culture plan design, and, in general, contribute to a more sustainable waterfowl house management approach.<br>The environmental parameters selected in the present study may also pose a limitation because of the lack of equipment. Future work may include the use of more environmental parameters to evaluate PI and apply the model in more waterfowl breeding sites to improve the production effect and further verify the model.
Additional Information:	
Question	Response
**Financial Disclosure**<br><br>Enter a financial disclosure statement that describes the sources of funding for the work included in this submission. Review the submission guidelines for detailed requirements. View published research articles from *PLOS ONE* for specific examples.<br><br>This statement is required for submission and **will appear in the published article** if the submission is accepted. Please make sure it is accurate.<br><br>**Unfunded studies**<br>Enter: *The author(s) received no specific funding for this work.*<br><br>**Funded studies**<br>Enter a statement with the following details:<br>• Initials of the authors who received each award<br>• Grant numbers awarded to each author<br>• The full name of each funder<br>• URL of each funder website<br>• Did the sponsors or funders play any role in the study design, data collection and analysis, decision to publish, or preparation of the manuscript?<br>• **NO** - Include this sentence at the end of your statement: *The funders had no role in study design, data collection and analysis, decision to publish, or preparation of the manuscript.*<br>• **YES** - Specify the role(s) played.<br><br>* typeset	This work was supported in part by<br>the National Natural Science Foundation of China under Grant 61871475,61471-131,61571444,<br>in part by the special project of laboratory construction of Guangzhou Innovation Platform Construction Plan under Grant 201905010006,<br>Guangzhou Innovation Plantform Construction Plan under Grant 2017B0101260016, foundation for<br>High-level Talents in Higher Education of Guangdong Province under Grant 2017GCZX00014, 2016K-ZDXM0013,2017KTSCX094,2018LM2168<br>,and Beijing Natural Science Foundation under Grant 4182023.
**Competing Interests**<br><br>Use the instructions below to enter a competing interest statement for this submission. On behalf of all authors,	The authors have declared that no competing interests exist.

Powered by Editorial Manager® and ProduXion Manager® from Aries Systems Corporation

disclose any competing interests that could be perceived to bias this work—acknowledging all financial support and any other relevant financial or non-financial competing interests.<br><br>This statement is required for submission and **will appear in the published article** if the submission is accepted. Please make sure it is accurate and that any funding sources listed in your Funding Information later in the submission form are also declared in your Financial Disclosure statement.<br><br>View published research articles from *PLOS ONE* for specific examples.<br><br>**NO authors have competing interests**<br><br>Enter: *The authors have declared that no competing interests exist.*<br><br>**Authors with competing interests**<br><br>Enter competing interest details beginning with this statement:<br><br>*I have read the journal's policy and the authors of this manuscript have the following competing interests: [insert competing interests here]*<br><br>* typeset	
**Ethics Statement**<br><br>Enter an ethics statement for this submission. This statement is required if the study involved:<br><br>• Human participants<br>• Human specimens or tissue<br>• Vertebrate animals or cephalopods<br>• Vertebrate embryos or tissues<br>• Field research<br><br>Write "N/A" if the submission does not require an ethics statement.	N / A

General guidance is provided below. Consult the submission guidelines for detailed instructions. **Make sure that all information entered here is included in the Methods section of the manuscript.**<br><br>**Format for specific study types**<br><br>**Human Subject Research (involving human participants and/or tissue)**<br>• Give the name of the institutional review board or ethics committee that approved the study<br>• Include the approval number and/or a statement indicating approval of this research<br>• Indicate the form of consent obtained (written/oral) or the reason that consent was not obtained (e.g. the data were analyzed anonymously)<br><br>**Animal Research (involving vertebrate animals, embryos or tissues)**<br>• Provide the name of the Institutional Animal Care and Use Committee (IACUC) or other relevant ethics board that reviewed the study protocol, and indicate whether they approved this research or granted a formal waiver of ethical approval<br>• Include an approval number if one was obtained<br>• If the study involved *non-human primates*, add *additional details* about animal welfare and steps taken to ameliorate suffering<br>• If anesthesia, euthanasia, or any kind of animal sacrifice is part of the study, include briefly which substances and/or methods were applied<br><br>**Field Research**<br><br>Include the following details if this study involves the collection of plant, animal, or other materials from a natural setting:<br>• Field permit number<br>• Name of the institution or relevant body that granted permission	
**Data Availability**<br><br>Authors are required to make all data	Yes - all data are fully available without restriction

Powered by Editorial Manager® and ProduXion Manager® from Aries Systems Corporation

underlying the findings described fully available, without restriction, and from the time of publication. PLOS allows rare exceptions to address legal and ethical concerns. See the PLOS Data Policy and FAQ for detailed information.<br><br>A Data Availability Statement describing where the data can be found is required at submission. Your answers to this question constitute the Data Availability Statement and **will be published in the article**, if accepted.<br><br>**Important:** Stating 'data available on request from the author' is not sufficient. If your data are only available upon request, select 'No' for the first question and explain your exceptional situation in the text box.<br><br>Do the authors confirm that all data underlying the findings described in their manuscript are fully available without restriction?	
**Describe where the data may be found in full sentences. If you are copying our sample text, replace any instances of XXX with the appropriate details.**<br><br>• If the data are **held or will be held in a public repository**, include URLs, accession numbers or DOIs. If this information will only be available after acceptance, indicate this by ticking the box below. For example: *All XXX files are available from the XXX database (accession number(s) XXX, XXX.*).<br>• If the data are all contained **within the manuscript and/or Supporting Information files**, enter the following: *All relevant data are within the manuscript and its Supporting Information files.*<br>• If neither of these applies but you are able to provide **details of access elsewhere**, with or without limitations, please do so. For example:<br><br>*Data cannot be shared publicly because of [XXX]. Data are available from the*	All relevant data are within the manuscript and its Supporting Information files.

XXX Institutional Data Access / Ethics Committee (contact via XXX) for researchers who meet the criteria for access to confidential data.<br><br>*The data underlying the results presented in the study are available from (include the name of the third party and contact information or URL).*<br>• This text is appropriate if the data are owned by a third party and authors do not have permission to share the data.<br><br>* typeset	
Additional data availability information:	

Powered by Editorial Manager® and ProduXion Manager® from Aries Systems Corporation

Manuscript Click here to access/download;Manuscript;Manuscript.docx

Pollution index of waterfowl farm assessment and prediction based on temporal convoluted network

Jiande Huang[1,2,3,4,5,6], Shuangyin Liu[1,2,3,4,5,6,*], Shahbaz Gul Hassan[1,2,3,4,5,6, *],Longqin Xu[1,2,3,4,5,6]

[1]College of Information Science and Technology, Zhongkai University of Agriculture and Engineering, Guangzhou,510225, China

[2]Smart Agriculture Engineering Technology Research Center of Guangdong Higher Education Institues, Zhongkai University of Agriculture and Engineering, Guangzhou, 510225, China

[3]Guangzhou Key Laboratory of Agricultural Products Quality. Safety Traceability Information Technology, Zhongkai University of Agriculture and Engineering, Guangzhou 510225, China

[4]Guangdong Provincial Agricultural Products Safety Big Data Engineering Technology Research Center, Zhongkai University of Agriculture and Engineering, Guangzhou 510225, China

[5]Guangdong Province Key Laboratory of Waterfowl Healthy Breeding, Guangzhou 510225, China

[6]Academy of Smart Agricultural Engineering Innovations, Zhongkai University of Agriculture and Engineering, Guangzhou 510225, China

[*] Corresponding author.

[*] Authors contributed equallys.

E-mail address: shuangyinliu@zhku.edu.cn , mhasan387@zhku.edu.cn

[#a]Current Address: College of Information Science and Technology, Zhongkai University of Agriculture and Engineering, Guangzhou,510225, China.

Abstract

Environmental quality is a major factor that directly impacts waterfowl productivity. Accurate prediction of PI is the key to improving environmental management and pollution control. This study applied a new neural network model called temporal convolutional network and a denoising algorithm called wavelet transform (WT) for predicting future 12-, 24-, and 48-hour PI values at a waterfowl farm in Shanwei, China. The temporal convoluted network (TCN) model performance was compared with that of recurrent architectures with the same capacity, long-short time memory neural network (LSTM), and gated recurrent unit (GRU). Denoised environmental data, including ammonia, temperature, relative humidity, carbon dioxide (CO_2), and total suspended particles (TSP), were used to construct the forecasting model. The simulation results showed that the TCN model in general produced a more precise PI prediction and provided the highest prediction accuracy for all phases (MAE=0.0842, 0.0859, and 0.1115; RMSE=0.0154, 0.0167, and 0.0273; R2=0.9789, 0.9791, and 0.9635). The PI assessment prediction model based on TCN exhibited the best prediction accuracy and general performance compared with other parallel forecasting models and is a suitable and useful tool for predicting PI in waterfowl farms.

Keywords: Agricultural informatization; environmental quality assessment; temporal convoluted network; regression prediction

Introduction

In response to the growing global demand for food, the Chinese waterfowl industry has grown to be a leader in both goose meat and goose egg production [1]. To meet this demand, one mechanism to increase production is to increase housing and manage more geese. However,

as the scale of intensive culture increases, there is a growing concern in China that waterfowl should be raised under conditions that promote animal welfare since productivity is related to environmental conditions in which the waterfowl are raised [2]. For example, the organic matter present in excreta and/or litter resulting in pollutant production, such as CO_2, ammonia, and TSP, will not only impair geese and staff health but also have important consequences for society once the pollutants are out in the atmosphere [3–4]. According to this, to maintain and control optimal conditions for survival and ~~good goose~~ growth, establishing a habitat that is closer to a standard ecosystem is justified [5].

Labor shortages and increasing biosecurity practices will make it more difficult for producers to monitor and manage the production, health, and welfare status of all of their birds. Employing modern poultry management technology is necessary to increase production [6]. An example of how modern management technology can be used to monitor and control the poultry house environment is exemplified by humidity regulation via ventilation rate changes mediated by relative humidity sensors, as relative humidity is one of the more important environmental aspects of a poultry house [7]. In addition, more advanced systems are being researched. Bustamante et al. used a multisensor system to effectively assess barn ventilation system function by tracking temperature, air velocity and differential pressure in broiler houses [8]. Hanif et al. proposed an internet of things technology-based protection and monitoring of the environment of a poultry house to monitor the environment-related parameters such as air temperature, air humidity, CO2 level of concentration and ammonia concentration, which has been implemented successfully, leading to a safe environment and profit for the poultry industry [9]. The techniques mentioned above are both solutions for real-time environmental monitoring

and control; however, relying on hardware monitoring in real time cannot capture the trend of environmental changes [10], and it is easy to miss the best time for adjustment, which leads to waterfowl health damage and property loss.

Cultivation environment forecasting has been studied for many years and has made some achievements in aquaculture and livestock breeding [11–14]. The technique estimates or predicts the future changes in target variables that cannot be obtained directly. For example, Jackman et al. generated a prediction model by using sensor inputs of relative humidity, carbon dioxide, temperature, and ammonia for environmental parameter and crop yield prediction [15]. In waterfowl production, a system such as this would allow for actions to be taken sooner by farmers if the environment is projected to be bad. However, few studies have applied prediction in waterfowl breeding. Therefore, it is necessary to apply environmental prediction technology to waterfowl production to fill this gap.

The pollution index (PI) is a simple and easy assessment method for assessing environmental quality, and precise environmental quality assessment and prediction allow better environmental management practices and contribute to a more sustainable environmental management approach [16-17]. In recent years, many studies on pollution models have been carried out to evaluate the quality of the environment based on the PI method, such as river quality status assessment and prediction [16,18–20], air quality status assessment and prediction [21–23], and soil pollution assessment and prediction [24-25]. Forecasting the concentration of water, air, and soil pollutants is an effective method for protecting public health, productivity, and travel by providing early warnings of harmful pollutants. Likewise, it is also important to assess and predict PI in advance for waterfowl farms so that, if necessary, the

producer can intervene more quickly by using management practices to ensure a healthy context.

In general, PI formulations include lengthy computations and thus require considerable time and effort. Additionally, waterfowl environment pollutant data are dynamic, complex, and have high temporal and spatial variability [26-27], and traditional forecasting methods such as multiple linear regression and autoregressive integrated moving average models have poor performance. Hence, a method to calculate PI in an efficient and precise way is required. Such approaches may benefit farmers when assessing and managing environmental quality.

Over the past few decades, artificial intelligence has been increasingly applied to solve various environmental engineering problems, including water quality modeling [28-29] and air quality modeling [30]. Among them, STM is a class of neural networks that can only use the current input information and historical information. Compared with other AI-based models, LSTM is powerful for modeling sequence data such as time series or natural language [31–33]. However, recursive neural networks such as LSTM and GRU process input sequences in parallel, so the cost of model training will increase with increasing length of input sequences. Moreover, distant historical memory will be forgotten, and they have a weak ability for temporal and spatial feature extraction [34]. Therefore, convolutional neural networks have attracted increasing attention in temporal and spatial sequence modeling. TCN [35], a model with a simple convolutional network architecture, is proposed to be applied to language modeling and music modeling and has demonstrated better performance than recurrent neural networks, especially in a long sequence. TCN has been proven to perform well in long sequence time series modeling. However, TCNs have not yet been applied in PI prediction. Therefore, a better TCN prediction architecture is explored to promote the predicted precision of PI in this

study.

Waterfowl house environment data usually contain many sources of noise. To eliminate noise interference, extract essential feature information, and obtain high-quality data sets, experts have proposed many methods, such as WT, independent component analysis, and empirical mode decomposition [36–38]. Wavelet transform can decompose time series with different resolutions and distinguish between noise and useful signals. It has been successfully applied to pattern recognition and noise elimination. Liu et al. proposed a hybrid wavelet analysis and least squares support vector regression with a Cauchy partial swarm optimization algorithm model for dissolved oxygen prediction [11]. Kumar et al. obtained better-quality denoised electrocardiogram signals by a denoising technique using a stationary wavelet transform compared with empirical mode decomposition, the Fourier decomposition method, and discrete wavelet transforms [39]. To effectively improve the GPR image resolution, Zhang et al. combined WT and F-K filtering [40]. Samuel et al. conducted a comparative analysis of a set of machine learning models, and the results showed that the best combination for predicting the streamflow into the Sobradinho Reservoir was the bootstrap, WT and neural network [41].

Based on the above studies, this paper proposes a new waterfowl house environmental quality assessment and prediction model combining wavelet transform and a temporal convolutional network. The wavelet transform can reduce the noise of original environmental data and obtain high-quality data, and the temporal convolutional network can extract the temporal and spatial features of processed data, output precise environmental quality assessment, and predict results.

Materials and Methods

Study area and data source

~~The~~ waterfowl culture farm in Haifeng County (23 °05’N, 115 °19’E) in Shanwei City, China was investigated in the present study. With an area of approximately 53.3 hm2, the farm is a multifunctional integrated aquacultural base integrating waterfowl breeding, seed~~ing~~ breeding and intensive aquaculture.

The environmental data were collected by the waterfowl culture internet of thing (IoT) online monitoring terminal system, and its architecture is shown in Fig. 1. The system is equipped with a temperature sensor, ammonia concentration sensor, humidity sensor, etc. The environmental information is uploaded to the cloud, and users can obtain on-site information by accessing the web server through mobile phones or personal computers.

Fig. 1 Schematic diagram of the waterfowl farm monitoring system based on the internet of things.

~~According to the~~ importance ~~of~~ the environment and expert research, we selected 5 environmental factors, as shown in Table 1. Among them, ammonia is a toxic gas and the greatest concern of environmental pollution in waterfowl production, adversely affecting the ecosystem, environment, and health of birds and people. Less than 10 ppm is the ideal limit [42]. Relative humidity can impact bird health, and high relative humidity may worsen broiler geese performance; the ideal value is 70% [43]. Heat stress is a major concern in waterfowl production; high and low temperatures will reduce the growth performance and survivability of waterfowl, and temperatures less than 27°C and more than 10°C are ideal [44]. TSP is ~~from~~ the birds themselves as well as from the feed, litter, and building materials and may serve as a

pathogen disseminator and bring about lung damage; less than 10 mg/m^3 is the ideal limit [42]. Additionally, the levels of relative humidity, ammonia, carbon dioxide, temperature and TSP are known to be correlated with each other [6]. These factors are important for waterfowl production and can be monitored and optimized.

Table 1 The selected environmental parameters for PI prediction.

Environmental factor	Unit	Limit	Influences
Ammonia	ppm	10	Ammonia is a toxic gas impairing animal performance and bird and staff health [41-42].
Relative humidity	%	70	High relative humidity may worsen broiler geese performance [43].
Temperature	°C	10~27	Influences poultry welfare and food intake, as well as increases susceptibility to disease and flock mortality rate [44].
CO_2	ppm	1500	A decrease in production and bird health can occur [45].
TSP	mg/m^3	8	A higher incidence of lung damage [42].

Data sets

In this report, environmental data, including ammonia, relative humidity, temperature, CO_2 and TSP, were collected from July 1st to September 28th, 2019, at intervals of 20 minutes. There were 72 sets of data collected per day with a total yield of 6480 observed samples. For model generation, the first 4536 sets of data were used for model training, and the remaining 1944 sets were used as the testing data to estimate the ~~prediction~~ performance of the constructed model.

Pollution index

The pollution index in this study was formulated within the criteria for evaluating the environmental quality of livestock and waterfowl farms by the Quality and Technology Supervision Bureau of China. The single pollution index can be calculated as:

$$I = {}^{C_i}\!\!\diagup_{C_{si}} \quad (1)$$

where C_i is the measured concentration of environmental pollutants and C_{si} is the standard concentration limit of pollutants. The composite pollution index of waterfowl house environment quality can be described as follows:

$$P_i = \left\{ \mathrm{I}_{\max} \cdot (1/n) \sum I_i \right\}^{1/2} \quad (2)$$

where I_{max} is the maximum single pollution index among all pollutants, n is the number of pollutants, and I_i is the single pollution index of pollutant i.

Wavelet transform

Continuous wavelet transforms for a given waterfowl environment signal *s(k)* can be described as follows:

$$WTs(a,b) = \frac{1}{\sqrt{a}} \int_{\infty}^{-\infty} s(k) \overline{\left(\psi\left(\frac{t-b}{a} \right) \right)} dx \quad (3)$$

where $\overline{(\ )}$ denotes the complex conjugate, $\psi(x)$ is the wavelet function, a is the time scale dilation and b is the time translation. By controlling the values of parameters a and b, signal time-frequency positioning can be achieved. The wavelet transform can decompose signals into multiple resolutions. However, the symbols in realization communication are discrete data, so the continuous wavelet transform needs to be discretized. Suppose $a = a_0^j, b = kb_0 a_0^j, j,k \in Z$, when $a_0 = 2, b_0 = 1$:

$$\psi_{j,k}(n) = 2^{-j/2} \psi(2^{-j} n - k) \quad (4)$$

The discrete waterfowl environment signal s(n) can be transformed as:

$$D(j,k) = \sum_k s(n) \overline{\psi_{j,k}}(n) \quad (5)$$

The Mallat algorithm can quickly calculate the orthogonal wavelet transform coefficients

and realize signal decomposition and reconstruction [47]. The approximation coefficients $s_{j+1}(n)$ and detail coefficients $d_{j+1}(n)$ can be recurrently related by the Mallat algorithm as follows:

$$s_{j+1}(k) = \sum_{n} h_0(n-2k)s_j(n) \tag{6}$$

$$d_{j+1}(k) = \sum_{n} h_1(n-2k)s_j(n) \tag{7}$$

where h_0 and h_1 are the high-pass filter and low-pass filter, respectively. The reconstruction is the inverse process of decomposition:

$$s_j(k) = \sum_{n} s_{j+1}(n)h_0(k-2n) + \sum_{n} d_{j+1}(n)h_1(k-2n) \tag{8}$$

Support vector machine

SVM has good generalization ability in solving nonlinear, small sample, and high-dimensional pattern recognition, and the optimal solution obtained is global, which solves the local optimal problem that cannot be avoided in other algorithms. The prediction process of the SVM includes support vector determination, kernel function selection, kernel parameter determination, and solution.

Random forest

RF is a nonlinear ensemble model that establishes and averages a large number of random distribution decision trees for regression or classification tasks [48]. A decision tree or classification and regression tree that constructs the RF is a nonparametric model. According to the complexity of the input data, the tree grows in the process of learning. Decision nodes and leaf nodes are the main components of the decision tree. Each input sample is estimated by a test function of decision

nodes and passed to different branches according to the features of the sample. After all trees are trained, each tree can predict the test sample set according to the node threshold, and the results of each tree are combined to vote to determine the final result of the entire random forest.

Long short-term memory neural network

The LSTM neural network is a special kind of recurrent neural network. It was first proposed by Ho-chreiter and Schmidhuber [49]. Its appearance effectively solved the gradient explosion problem of traditional recurrent neural networks. At the same time, the LSTM neural network has long-term memory to handle long-term sequence data.

Gated recurrent unit

A gated recurrent unit (GRU) is a type of recurrent neural network (RNN). Similar to a long short-term memory neural network (LSTM), it is also proposed to solve long-term and gradient backpropagation problems. LSTM and GRU have similar performances, but compared with LSTM, GRU is computationally cheaper.

Temporal convoluted network

TCN, like LSTM, is a novel neural network architecture that can be used for time series prediction. The outstanding advantage of TCNs is that they not only have much longer memory but also have higher computational efficiency than LSTM and other recurrent neural networks [50].

In general, a nature sequence modeling task is any function $f{:}X^{T+1} \to Y^{T+1}$ that produces the mapping:

$$y_0, y_1, \ldots, y_T = f(x_0, x_1, \ldots, x_t) \tag{9}$$

The goal of the sequence model is to fit this function f to minimize the expected loss. It satisfies

the causal constraint that y_t depends only on $x_0, x_1, \ldots, x_t$ and not on future inputs x_{t+1} and that the output has the same length as the input.

As shown in Fig. 2, to satisfy the causal constraint, TCN uses a 1D fully convolutional network ~~(FCN)~~ architecture [51], which is different from the traditional convoluted neural network in that the value at time *t* only depends on the value at time *t* and before in the previous layer. In addition, zero padding of length (kernel size - 1) is added to keep subsequent layers the same length as previous layers.

Fig. 2 Causal convolution construction.

One of the goals of TCNs is a long effective history size, which means an extremely deep network or very large filters. However, more convolution layers or larger filters bring about the problems of disappearing gradients, complex training, and poor fitting effects. To solve the problems above, dilated convolutions [35] were employed in the TCN. Specifically, given a sequence input $X^{n+1} = \{x_0, x_1, \ldots, x_n\}$ and convolution function $f: \{0, 1, \ldots, k-1\} \to \mathbb{R}$, the dilated convolution operation was defined as follows:

$$F(s) = \sum_{i=0}^{k-1} f(i) \cdot x_{s-i \cdot d} \tag{10}$$

where *k* is the kernel size, *d* is the dilated factor, and $s - i \cdot d$ accounts for the direction of the past. When *d=1,* a dilated convolution is equal to a regular convolution. As shown in Fig. 3, as the number of layers increases, the dilated factor *d* grows, and the top layer can represent a wider range of inputs. On the other hand, choosing a larger kernel size *k* of the filter can also effectively expand the receptive field of a ConvNet.

Fig. 3 Dilated convolution construction.

Residual connections proved to be an effective method for deep network training to converge quickly and reduce the risk of overfitting [52]. As shown in Fig. 4, the residual block used in the TCN has two branches from input to output. The first branch contains a series convolution layer, parameter regularization, rectified linear unit, and dropout layer in order. This was a flexible architecture that allows the layer to modify parameters such as the activation function and dropout rate. The second branch ensures that the output sequence length is equal to the length of the input: if the lengths of the input sequence and output sequence are equal, the output layer is connected to the input layer through identity mapping; otherwise, the output layer is connected to the input layer through a 1×1 convolution.

Fig. 4 An example of residual connection in a TCN.

Processing of environmental data

Different environmental factors may contain different kinds of noise. To achieve the best effect of the denoising process and obtain quality data, we adopt four wavelet functions, Db4, Haar, Coif, and Sym10, to process each environmental dataset. The signal-noise ratio (SNR) and normalized cross correlation (NCC) were used to evaluate the denoised effect, which can be described as:

$$SNR = \frac{{I_1}^2}{(I_2 - I_1)^2} \tag{11}$$

$$NCC = \frac{\sum (I_1 - mean(I_1)) \cdot (I_2 - mean(I_2))}{\sqrt{\sum (I_1 - mean(I_1))^2 \times \sum (I_2 - mean(I_2))^2}} \tag{12}$$

where I_1 is the denoised signal and I_2 is the original signal; theoretically, the larger the SNR and NCC are, the better the noise reduction effect. As shown in Table 2, wavelet function

Db4 was suitable for ammonia data (SNR=9.4318, NCC=0.9485), Sym10 was suitable for temperature data (SNR=7.4635, NCC=0.9221), relative humidity data (SNR=4.0809, NCC=0.8630), and CO_2 data (SNR=1.7785, NCC=0.7793), and Coif was suitable for TSP data (SNR=13.1443, NCC=0.9771). The best denoised results of each environmental dataset are shown in Fig. 5.

Table 2 Denoised performance of five parameters.

Target	Performance criteria	Wavelet function			
		Db4	Haar	Coif	Sym10
Ammonia	SNR/(dB)	9.4318	8.8287	9.3769	9.3602
	NCC	0.9485	0.9432	0.9484	0.9474
Temperature	SNR/(dB)	7.2491	6.5372	7.0205	7.4635
	NCC	0.9192	0.9061	0.9149	0.9221
Relative humidity	SNR/(dB)	3.9422	3.6395	3.8920	4.0809
	NCC	0.8600	0.8559	0.8596	0.8630
CO_2	SNR/(dB)	1.7064	1.3855	1.4991	1.7785
	NCC	0.7764	0.7642	0.7689	0.7793
TSP	SNR/(dB)	12.8848	12.6578	13.1443	13.1142
	NCC	0.9756	0.9745	0.9771	0.9769

(a) Ammonia sequence.

(b) Temperature sequence.

(c) Relative humidity sequence.

(d) CO_2 sequence.

(e) TSP sequence.

Fig. 5 Results of noise reduction of five parameters by WT.

Performance criteria

In this paper, the mean absolute error (MAE), root mean square error (RMSE) and coefficient of determination (R^2) were selected to measure the prediction accuracy and operation efficiency, and MAE, RMSE and R^2 are defined as follows:

$$MAE = \frac{1}{N}\sum_{i=1}^{N}|y_i - y_i'| \tag{13}$$

$$RMSE = \sqrt{\frac{1}{N}\sum_{i=1}^{N}(y_i - y_i')^2} \tag{14}$$

$$R^2 = \frac{\left(\sum_{i=1}^{N}(y_i - \overline{y})(y_i' - \overline{y}')\right)^2}{\sum_{i=1}^{N}(y_i - \overline{y})^2 \cdot \sum_{i=1}^{N}(y_i' - \overline{y}')^2} \tag{15}$$

where N is the total number of actual points in the data, *yi* is the observed value of period i, y_i is the prediction value of period i, y is the average of observed values, and y_t is the average of prediction values.

The waterfowl house environment quality assessment and prediction model

The algorithms used in this paper were implemented in Python 3.7 programming language. The equipment used in this work has an Intel Core i5-5200u processor, CPU @2.20 GHz and 8.0 GB of random access memory installed.

Both WT and TCN have the unique advantage of being able to capture data characteristics in time series. Thus, this paper uses WT-TCN to construct a model to assess and forecast the pollution index of waterfowl houses. The implementation process for our model is shown in

Fig. 6. In this study, we first reduce or eliminate the noise of environmental data by wavelet transform. Then, the denoised data and pollution index data were used as the input to train the TCN, and finally, the waterfowl house environment quality assessment and prediction model was obtained.

Fig. 6 Construction process of the PI prediction model.

Results and discussion

Simulation results and discussion

In this study, we tested the TCN's memory and feature extraction capability for environment data sequences of different lengths. Fig. 7 shows that TCN consistently converges to approximately 0.01 MSE for all sequence lengths, whereas GRU and LSTM degenerate quickly as the sequence lengths grow. These results suggest that TCN is better at long sequence memory and feature extraction than its recurrent counterparts.

Fig. 7 Performance of three models in sequences of different lengths.

Then, TCNs were used to predict PI at different time intervals, including 12 hours, 24 hours, and 48 hours (predicting 12 hours, 24 hours and 48 hours in the future every 20 minutes). Table 3 shows that TCN has the best performance in all simulation results compared with the other models. We noted that as the prediction time interval increased, the prediction effect decreased. This can be expected because a long prediction time interval needs a much longer effective history, which is a challenge for time series models. The performance of TCN fluctuates when the prediction time interval changes from 12 hours to 24 hours, but the wave motion ranges in MAE, RMSE and R_2 of TCN and GRU are less than 10%. However, the wave

motion range in MAE, RMSE and R2 of LSTM reached 25% when the prediction time interval changed from 12 hours to 24 hours and reached 112% when the prediction time interval changed from 24 hours to 48 hours. Moreover, the traditional SVM and RF models had poor performance in this study, which may be caused by the lack of long-time series memory ability compared with the recurrent neural network. In addition, scatter plots, Taylor plots and box plots were also used here to visualize the predictive performance of various models.

Table 3 Comparison of model performance.

	MAE			RMSE			R^2		
Hours	12	24	48	12	24	48	12	48	48
TCN	0.0842	0.0859	0.1115	0.0154	0.0167	0.0273	0.9789	0.9791	0.9635
GRU	0.1728	0.1810	0.1922	0.0759	0.0789	0.0789	0.8937	0.8903	0.8898
LSTM	0.1892	0.2523	0.4434	0.0824	0.1388	0.2953	0.8896	0.8082	0.6078
SVM	0.5919	0.7245	0.8991	0.3292	0.3537	0.8181	0.8512	0.7748	0.6617
RF	0.2744	0.5794	0.9571	0.3407	0.3555	0.7513	0.8809	0.8050	0.6799

The scatter plot in Fig. 8 visualized the agreement between the predicted and observed values of PI. In Fig. 8, the baseline is drawn as a reference, and the perfect agreement between the observed and predicted data is described. SVM and RF models are far away from the best line. All three TCN models showed outstanding prediction performance (points close to the best line). Additionally, in the three phases, TCN is slightly far from the best line when PI reaches 3.5 or more, but GRU and LSTM perform worse. The reason for this phenomenon may

be the input data of the environmental parameter CO_2; all were in the normal range and no data exceeded the standard value or were distributed near the border value (Fig. 5(d)), leading to the trained models having low sensitivity for a high PI and prediction difficulty.

(a) Future 12-hour prediction. **(b) Future 24-hour prediction.**

(c) Future 48-hour prediction.

Fig. 8 Agreement between the observed and predicted PI values for the model considered in this study.

Furthermore, the models were also evaluated using Taylor plots for the three phases in Fig. 9. In a Taylor figure correlation coefficient, normalized standard deviation and RMSE were drawn, and the distance between the point corresponding to the model with the best prediction performance and the "observation" point was the least. Again, Taylor plots showed that the TCN model has the best prediction performance.

(a) Future 12-hour prediction. **(b) Future 24-hour prediction.**

(c) Future 48-hour prediction.

Fig. 9 Normalized Taylor diagrams.

Finally, Fig. 10 compares the observed and predicted PI value dispersion and indicates the median (*M*) in a box plot. The performance of the three models was very similar in phase one, with the median values being very close ($M_{observed}=1.612$, $M_{TCN}=1.602$, $M_{GRU}=1.648$, $M_{LSTM}=1.719$,$M_{SVM}=1.569$, and $M_{RF}=1.854$), but the box shape of TCN is closer to the box shape of the observed data, which also means that all prediction results of TCN were better than the others. In both phase two and phase three, the performances of LSTM and GRU obviously worsen as the prediction interval grows, while the TCN performance does not change much. Overall, TCN substantially

outperforms generic recurrent architectures such as LSTM and GRU.

(a) Future 12-hour prediction. **(b) Future 24-hour prediction.**

(c) Future 48-hour prediction.

Fig. 10 Box plot of observed and predicted PI values.

In summary, TCN combines best practices such as dilations and residual connections with the causal convolutions needed for autoregressive prediction. The experimental results indicate that for all prediction time interval phases, the TCN model provides high performance for PI forecasting, especially in the long prediction time interval problem. On the other hand, the LSTM model appears to be the ‘weakest’ model of all three models; furthermore, it also indicates that a simple convolutional architecture is more effective across time sequence modeling tasks than recurrent architectures such as LSTM. ~~On the other hand~~, ~~a~~ new type of temporal convoluted neural network is more competitive in the PI time series prediction of waterfowl farms than the traditional machine learning model.

Conclusions and future research

To further promote waterfowl house environment monitoring and controlling technology, reduce labor and increase the production effect, this study ~~analyses~~ the shortcomings of existing methods and introduces a new way to guide waterfowl house environment management by ~~learning~~ from other fields.

The new method investigates the application of denoised WT and the performance of three neural network models and two mechanical learning models in predicting the pollution index of the waterfowl house environment using environmental quality parameters at different intervals. The results indicate that the TCN model has the best performance in predicting PI.

The GRU model has similar performance but lower performance when the prediction time interval changed, and the LSTM model performed the worst among the three models, although it still provided fairly accurate PI predictions.

The models presented in this paper, in particular the TCN model, could provide accurate and long-interval PI predictions of waterfowl house environments ~~and monitor them~~ in real time. The simulation results show that this method can be applied in waterfowl house environment prediction. The future trend of the environment can be estimated and predicted compared with traditional real-time monitoring technology, which may allow better waterfowl house environment management practices, better culture plan design, and, in general, contribute to a more sustainable waterfowl house management approach.

The environmental parameters selected in the present study ~~may also~~ pose a limitation because of the lack of equipment. Future work may include the use of more environmental parameters to evaluate PI and apply the model in more waterfowl breeding sites to improve the production effect and further verify the model.

Acknowledgments

The authors would like to thank native English experts Michele Genovese, Dr. Murtaza Hasan and AJE for revising the manuscript. Finally, this work was supported in part by the National Natural Science Foundation of China under Grants 61871475, 61471-131, and 61571444, in part by the special project of laboratory construction of Guangzhou Innovation Platform Construction Plan under Grant 201905010006, Guangzhou Innovation Platform Construction Plan under Grant 2017B0101260016, Foundation for High-level Talents in Higher Education of Guangdong Province under Grants 2017GCZX00014, 2016K-ZDXM0013,

2017KTSCX094, and 2018LM2168, and Beijing Natural Science Foundation under Grant 4182023.

References

1. CSB CSB. Statistical Communique of the People's Republic of China on the 2019 National Economic and Social Development. 2020.
2. Qian Y, Song K, Hu T, Ying T. Environmental status of livestock and poultry sectors in China under current transformation stage. Sci Total Environ. 2018. doi:10.1016/j.scitotenv.2017.12.045
3. Valdebenito B ÁM, Pal S, Behrendt A, Wulfmeyer V, Lammel G. A novel approach for the characterisation of transport and optical properties of aerosol particles near sources - Part II: Microphysics-chemistry-transport model development and application. Atmos Environ. 2011. doi:10.1016/j.atmosenv.2010.09.004
4. Behrendt A, Pal S, Wulfmeyer V, Valdebenito B. ÁM, Lammel G. A novel approach for the characterization of transport and optical properties of aerosol particles near sources - Part I: Measurement of particle backscatter coefficient maps with a scanning UV lidar. Atmos Environ. 2011. doi:10.1016/j.atmosenv.2011.02.061
5. Pereira WF, Fonseca L da S, Putti FF, Góes BC, Naves L de P. Environmental monitoring in a poultry farm using an instrument developed with the internet of things concept. Comput Electron Agric. 2020. doi:10.1016/j.compag.2020.105257
6. Astill J, Dara RA, Fraser EDG, Roberts B, Sharif S. Smart poultry management: Smart sensors, big data, and the internet of things. Computers and Electronics in Agriculture.

2020. doi:10.1016/j.compag.2020.105291
7. Conley S. Poultry Barn Meteorology Managing air quality through humidity control. 2012. Available: https://www.canadianpoultrymag.com/%0Aproduction/bird-management/poultry-barn-meteorology-12641
8. Bustamante E, Guijarro E, García-Diego FJ, Balasch S, Hospitaler A, Torres AG. Multisensor system for isotemporal measurements to assess indoor climatic conditions in poultry farms. Sensors (Switzerland). 2012. doi:10.3390/s120505752
9. Lashari MH, Memon AA, Shah SAA, Nenwani K, Shafqat F. IoT Based poultry environment monitoring system. Proceedings - 2018 IEEE International Conference on Internet of Things and Intelligence System, IOTAIS 2018. 2019. doi:10.1109/IOTAIS.2018.8600837
10. Li D, Yang H. State-of-the-art Review for Internet of Things in Agriculture. Nongye Jixie Xuebao/Transactions of the Chinese Society for Agricultural Machinery. 2018. doi:10.6041/j.issn.1000-1298.2018.01.001
11. Liu S, Xu L, Jiang Y, Li D, Chen Y, Li Z. A hybrid WA-CPSO-LSSVR model for dissolved oxygen content prediction in crab culture. Eng Appl Artif Intell. 2014;29: 114–124. doi:10.1016/j.engappai.2013.09.019
12. Li C, Li Z, Wu J, Zhu L, Yue J. A hybrid model for dissolved oxygen prediction in aquaculture based on multi-scale features. Inf Process Agric. 2018. doi:10.1016/j.inpa.2017.11.002
13. Yang L, Liu C, Guo Y, Deng H, Li D, Duan Q. Prediction of Ammonia Concentration in Fattening Piggery Based on EMD-LSTM. Nongye Jixie Xuebao/Transactions

Chinese Soc Agric Mach. 2019. doi:10.6041/j.issn.1000-1298.2019.S0.054

14. Liu CH, Yang L, Deng H, Guo YC, Li DL, Duan QL. Prediction of ammonia concentration in piggery based on ARIMA and BP neural network. Zhongguo Huanjing Kexue/China Environ Sci. 2019.

15. Jackman P, Ward S, Brennan L, Corkery G, McCarthy U. Application of wireless technologies to forward predict crop yields in the poultry production chain. Agric Eng Int CIGR J. 2015.

16. Asadollah SBHS, Sharafati A, Motta D, Yaseen ZM. River water quality index prediction and uncertainty analysis: A comparative study of machine learning models. J Environ Chem Eng. 2020. doi:10.1016/j.jece.2020.104599

17. Odiete WE. Novel metric for managing the protection of humanity and the environment against pollution and its adverse effects. Heliyon. 2020;6: e05555. doi:https://doi.org/10.1016/j.heliyon.2020.e05555

18. Martinus Y, Astono W, Hendrawan D. Water quality study of Sunter River in Jakarta, Indonesia. IOP Conference Series: Earth and Environmental Science. 2018. doi:10.1088/1755-1315/106/1/012022

19. Rubio-Arias H, Contreras-Caraveo M, Quintana RM, Saucedo-Teran RA, Pinales-Munguia A. An overall water quality index (WQI) for a man-made aquatic reservoir in Mexico. Int J Environ Res Public Health. 2012. doi:10.3390/ijerph9051687

20. Suriadikusumah A, Mulyani O, Sudirja R, Sofyan ET, Maulana MHR, Mulyono A. Analysis of the water quality at Cipeusing river, Indonesia using the pollution index method. Acta Ecol Sin. 2020. doi:10.1016/j.chnaes.2020.08.001

21. Leong WC, Kelani RO, Ahmad Z. Prediction of air pollution index (API) using support vector machine (SVM). J Environ Chem Eng. 2020. doi:10.1016/j.jece.2019.103208

22. Thach TQ, Tsang H, Cao P, Ho LM. A novel method to construct an air quality index based on air pollution profiles. Int J Hyg Environ Health. 2018. doi:10.1016/j.ijheh.2017.09.012

23. Khanoranga, Khalid S. Phytomonitoring of air pollution around brick kilns in Balochistan province Pakistan through air pollution index and metal accumulation index. J Clean Prod. 2019. doi:10.1016/j.jclepro.2019.05.050

24. Yang H, Wang F, Yu J, Huang K, Zhang H, Fu Z. An improved weighted index for the assessment of heavy metal pollution in soils in Zhejiang, China. Environ Res. 2021. doi:10.1016/j.envres.2020.110246

25. Jorfi S, Maleki R, Jaafarzadeh N, Ahmadi M. Pollution load index for heavy metals in Mian-Ab plain soil, Khuzestan, Iran. Data Br. 2017. doi:10.1016/j.dib.2017.10.017

26. Ramsey DSL, O’Brien DJ, Smith RW, Cosgrove MK, Schmitt SM, Rudolph BA. Management of on-farm risk to livestock from bovine tuberculosis in Michigan, USA, white-tailed deer: Predictions from a spatially-explicit stochastic model. Prev Vet Med. 2016. doi:10.1016/j.prevetmed.2016.09.022

27. Choi HL, Sudiarto SIA, Renggaman A. Prediction of livestock manure and mixture higher heating value based on fundamental analysis. Fuel. 2014. doi:10.1016/j.fuel.2013.08.064

28. Kargar K, Samadianfard S, Parsa J, Nabipour N, Shamshirband S, Mosavi A, et al.

Estimating longitudinal dispersion coefficient in natural streams using empirical models and machine learning algorithms. Eng Appl Comput Fluid Mech. 2020. doi:10.1080/19942060.2020.1712260

29. Alizadeh MJ, Kavianpour MR, Danesh M, Adolf J, Shamshirband S, Chau KW. Effect of river flow on the quality of estuarine and coastal waters using machine learning models. Eng Appl Comput Fluid Mech. 2018. doi:10.1080/19942060.2018.1528480

30. Seng D, Zhang Q, Zhang X, Chen G, Chen X. Spatiotemporal prediction of air quality based on LSTM neural network. Alexandria Eng J. 2021;60: 2021–2032. doi:https://doi.org/10.1016/j.aej.2020.12.009

31. Csábrági A, Molnár S, Tanos P, Kovács J. Application of artificial neural networks to the forecasting of dissolved oxygen content in the Hungarian section of the river Danube. Ecol Eng. 2017;100: 63–72. doi:10.1016/j.ecoleng.2016.12.027

32. Ranković V, Radulović J, Radojević I, Ostojić A, Čomić L. Neural network modeling of dissolved oxygen in the Gruža reservoir, Serbia. Ecol Modell. 2010;221: 1239–1244. doi:10.1016/j.ecolmodel.2009.12.023

33. Sun M, Hassan SG, Li D. Models for estimating feed intake in aquaculture: A review. Comput Electron Agric. 2016;127: 425–438. doi:10.1016/j.compag.2016.06.024

34. Peng Q, Liu Z-W. Short-Term Residential Load Forecasting Based on Smart Meter Data Using Temporal Convolutional Networks. 2020 39th Chinese Control Conference (CCC). 2020. pp. 5423–5428. doi:10.23919/CCC50068.2020.9188453

35. Bai S, Kolter JZ, Koltun V. An empirical evaluation of generic convolutional and recurrent networks for sequence modeling. arXiv. 2018.

36. Bavkar S, Iyer B, Deosarkar S. Detection of Alcoholics using EEG and EMD Domain Statistical Features. 2020 International Conference on Emerging Smart Computing and Informatics, ESCI 2020. 2020. pp. 153–157. doi:10.1109/ESCI48226.2020.9167564
37. Yan X, Boudrias MH, Mitsis GD. Artifact Removal in tACS-EEG Recordings: A Combined Methodology Based on the Empirical Wavelet Transform. Proceedings of the Annual International Conference of the IEEE Engineering in Medicine and Biology Society, EMBS. 2020. pp. 944–947. doi:10.1109/EMBC44109.2020.9176488
38. You SD, Hung MJ. Reducing Dimensionality of Spectro-Temporal Data by Independent Component Analysis. 2020 2nd International Conference on Computer Communication and the Internet, ICCCI 2020. 2020. pp. 93–97. doi:10.1109/ICCCI49374.2020.9145984
39. Kumar A, Tomar H, Mehla VK, Komaragiri R, Kumar M. Stationary wavelet transform based ECG signal denoising method. ISA Trans. 2020. doi:https://doi.org/10.1016/j.isatra.2020.12.029
40. Zhang L, Ling T, Yu B, Huang F, Zhang S. Intensive Interferences Processing for GPR signal based on the Wavelet Transform and F-K Filtering. J Appl Geophys. 2021. doi:10.1016/j.jappgeo.2021.104273
41. Saraiva SV, Carvalho F de O, Santos CAG, Barreto LC, Freire PK de MM. Daily streamflow forecasting in Sobradinho Reservoir using machine learning models coupled with wavelet transform and bootstrapping. Appl Soft Comput. 2021. doi:10.1016/j.asoc.2021.107081
42. David B, Moe RO, Michel V, Lund V, Mejdell C. Air Quality in Alternative Housing

Systems May Have an Impact on Laying Hen Welfare. Part I—Dust. 2015; 495–511. doi:10.3390/ani5030368

43. Najafi P, Zulkifli I, Amat Jajuli N, Farjam AS, Ramiah SK, Amir AA, et al. Environmental temperature and stocking density effects on acute phase proteins, heat shock protein 70, circulating corticosterone and performance in broiler chickens. Int J Biometeorol. 2015. doi:10.1007/s00484-015-0964-3

44. Gebregeziabhear E, Ameha N. The effect of stress on productivity of animals: a review. J Biol Agric Healthc. 2015;5: 165–172.

45. David B, Mejdell C, Michel V, Lund V, Moe RO. Air quality in alternative housing systems may have an impact on laying hen welfare. Part II-ammonia. Animals. 2015. doi:10.3390/ani5030389

46. K.Othman J, R. Mahmood J, Y. A. Al- Emarah G. Design and Implementation of Smart Relay based Remote Monitoring and Controlling of Ammonia in Poultry Houses. Int J Comput Appl. 2014. doi:10.5120/18093-9149

47. Mallat SG. A Theory for Multiresolution Signal Decomposition: The Wavelet Representation. IEEE Trans Pattern Anal Mach Intell. 1989. doi:10.1109/34.192463

48. Breiman L. Random forests. Mach Learn. 2001. doi:10.1023/A:1010933404324

49. Sepp H, J?urgen S. 1997Hochreiter_LSTM. Neural Comput. 1997.

50. Zhang Z, Chen H, Huang Y, Lee WJ. Quantile Huber Function Guided TCN for Short-Term Consumer-Side Probabilistic Load Forecasting. 2020 IEEE/IAS Industrial and Commercial Power System Asia, I and CPS Asia 2020. 2020. doi:10.1109/ICPSAsia48933.2020.9208545

51. Long J, Shelhamer E, Darrell T. Fully convolutional networks for semantic segmentation. Proceedings of the IEEE Computer Society Conference on Computer Vision and Pattern Recognition. 2015. doi:10.1109/CVPR.2015.7298965

52. He K, Zhang X, Ren S, Sun J. Deep residual learning for image recognition. Proceedings of the IEEE Computer Society Conference on Computer Vision and Pattern Recognition. 2016. doi:10.1109/CVPR.2016.90

Fig1

Click here to access/download;Figure;Fig1.tif

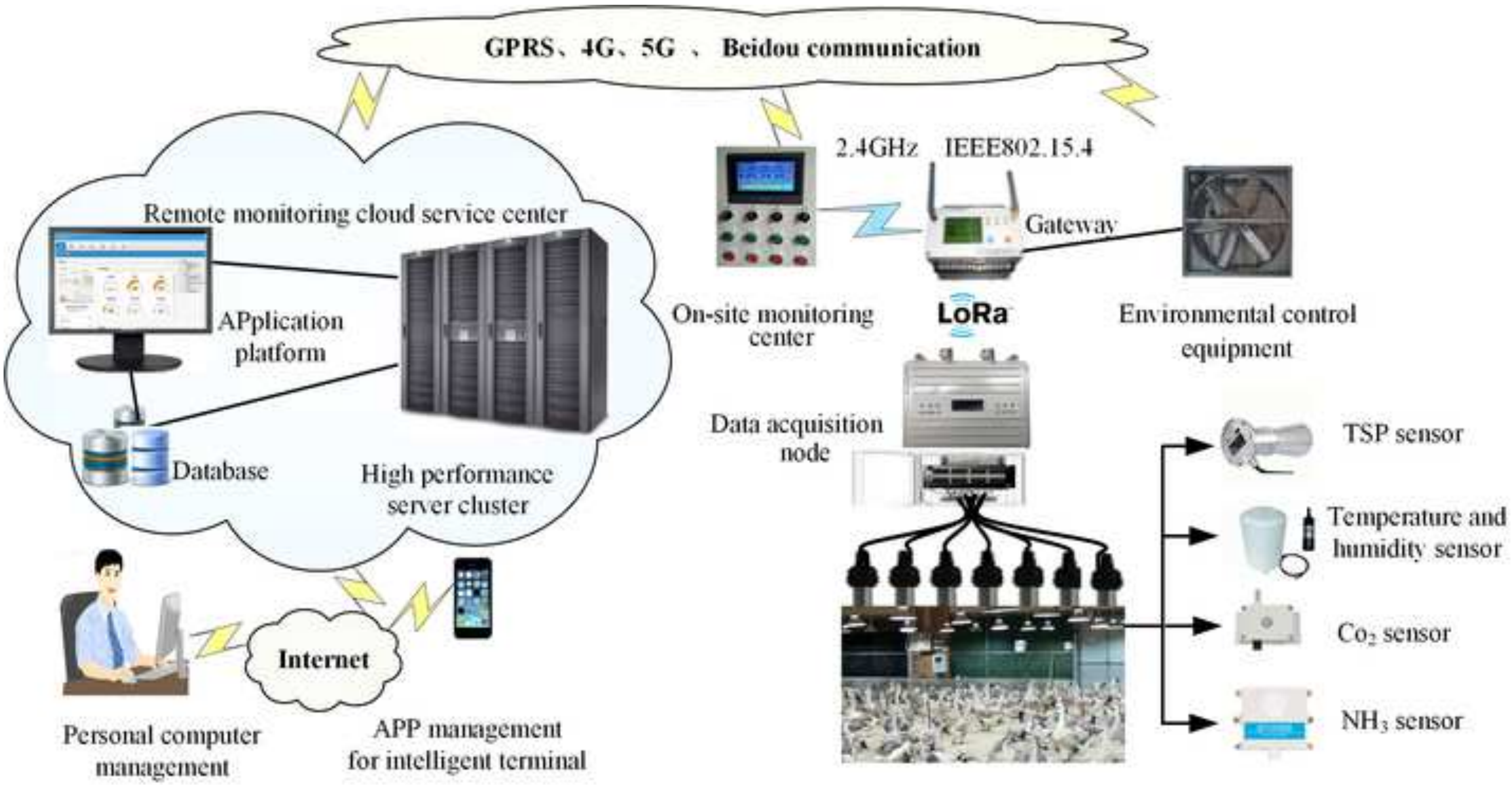

Fig2

Click here to access/download;Figure;Fig2.tif

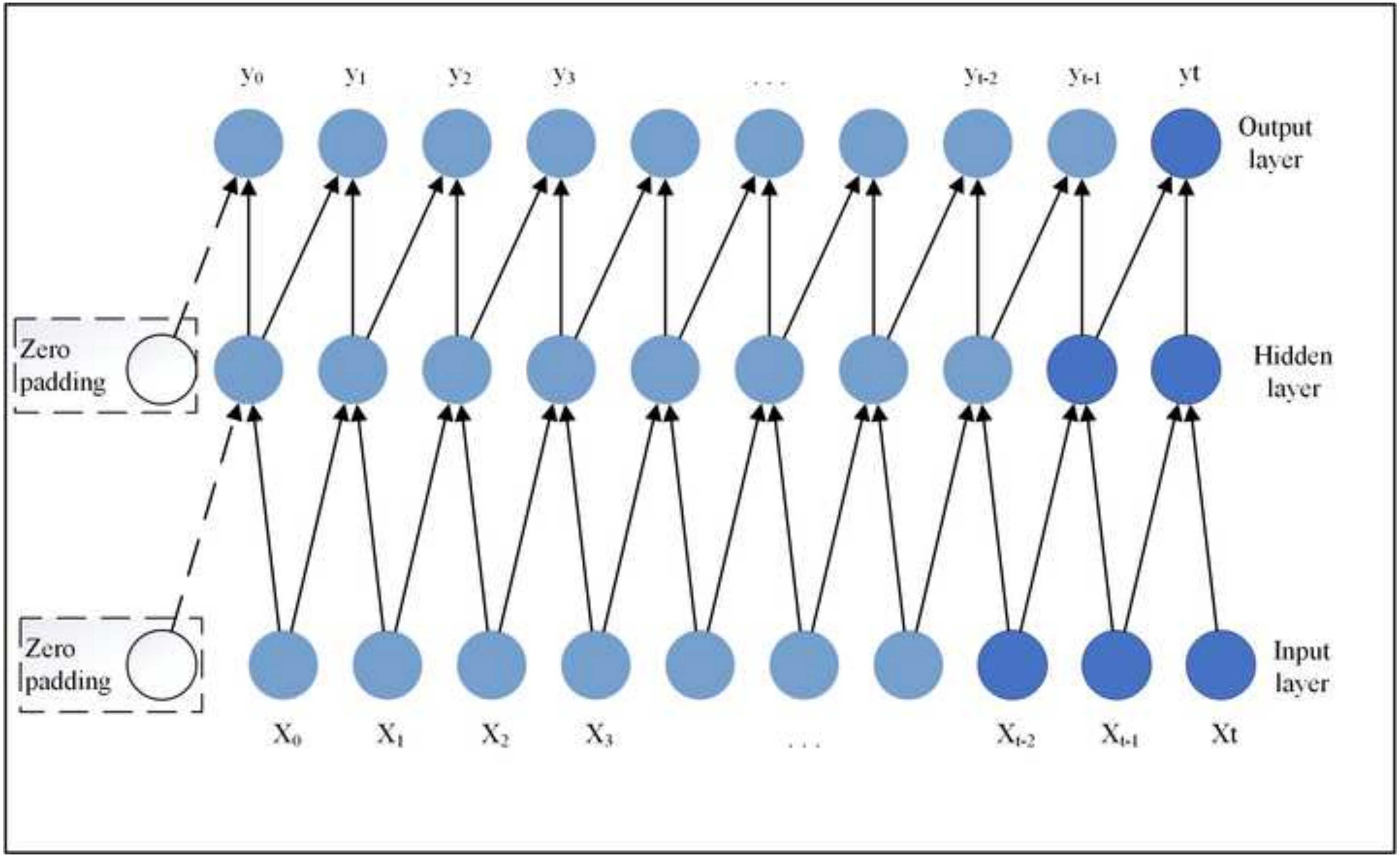

Click here to access/download;Figure;Fig3.tif

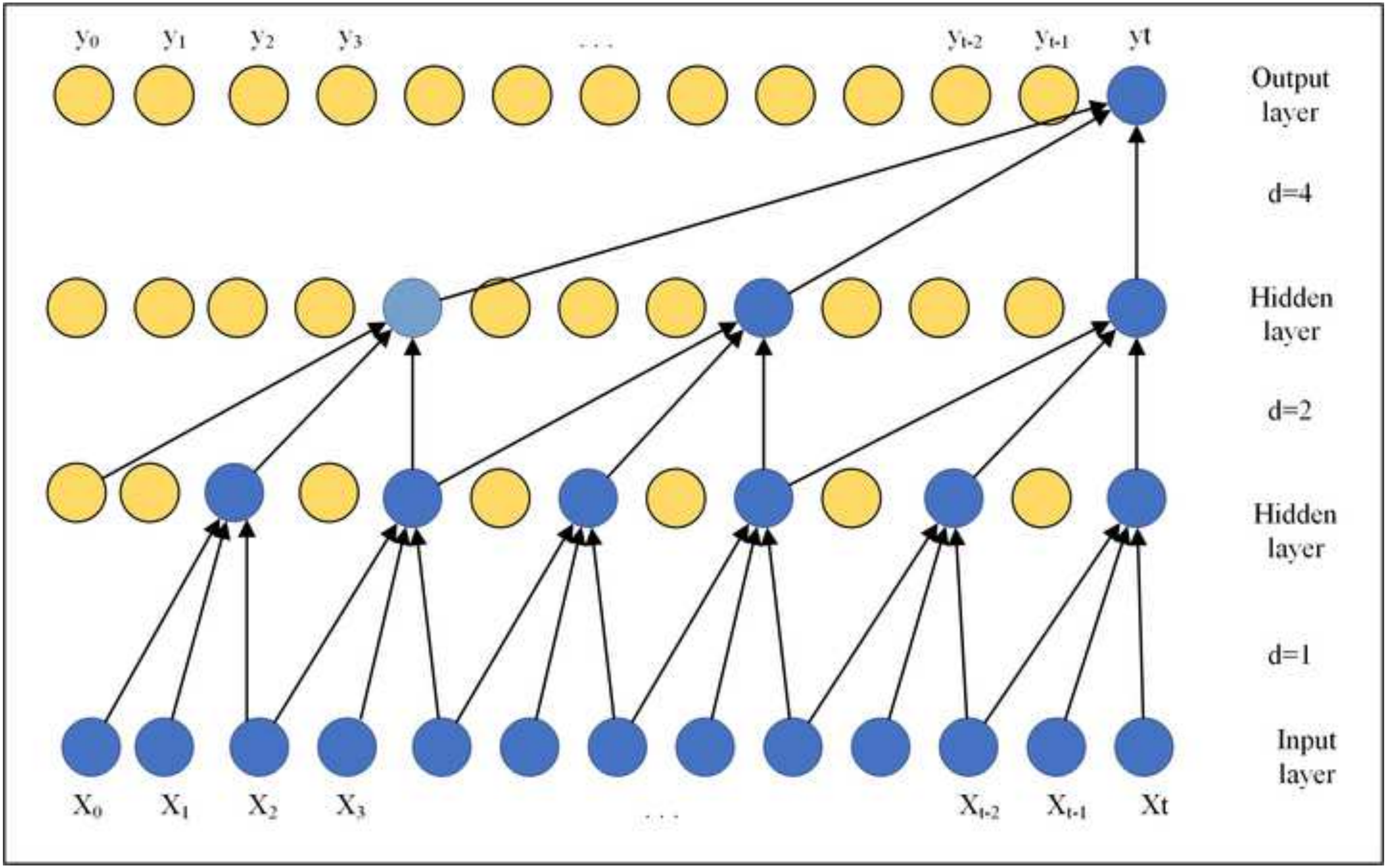

Fig4

Click here to access/download;Figure;Fig4.tif

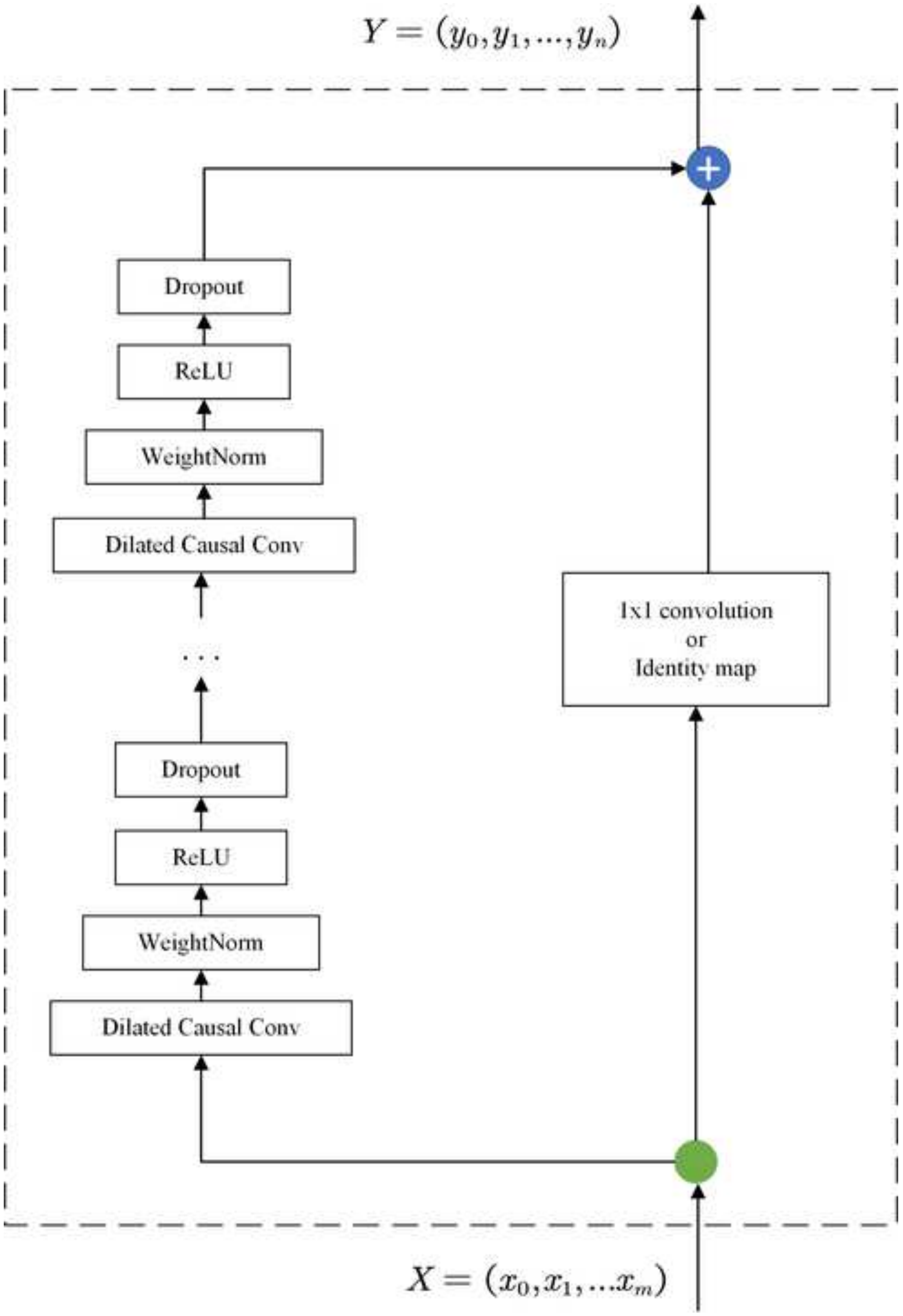

Fig5

Click here to access/download;Figure;Fig5.tif

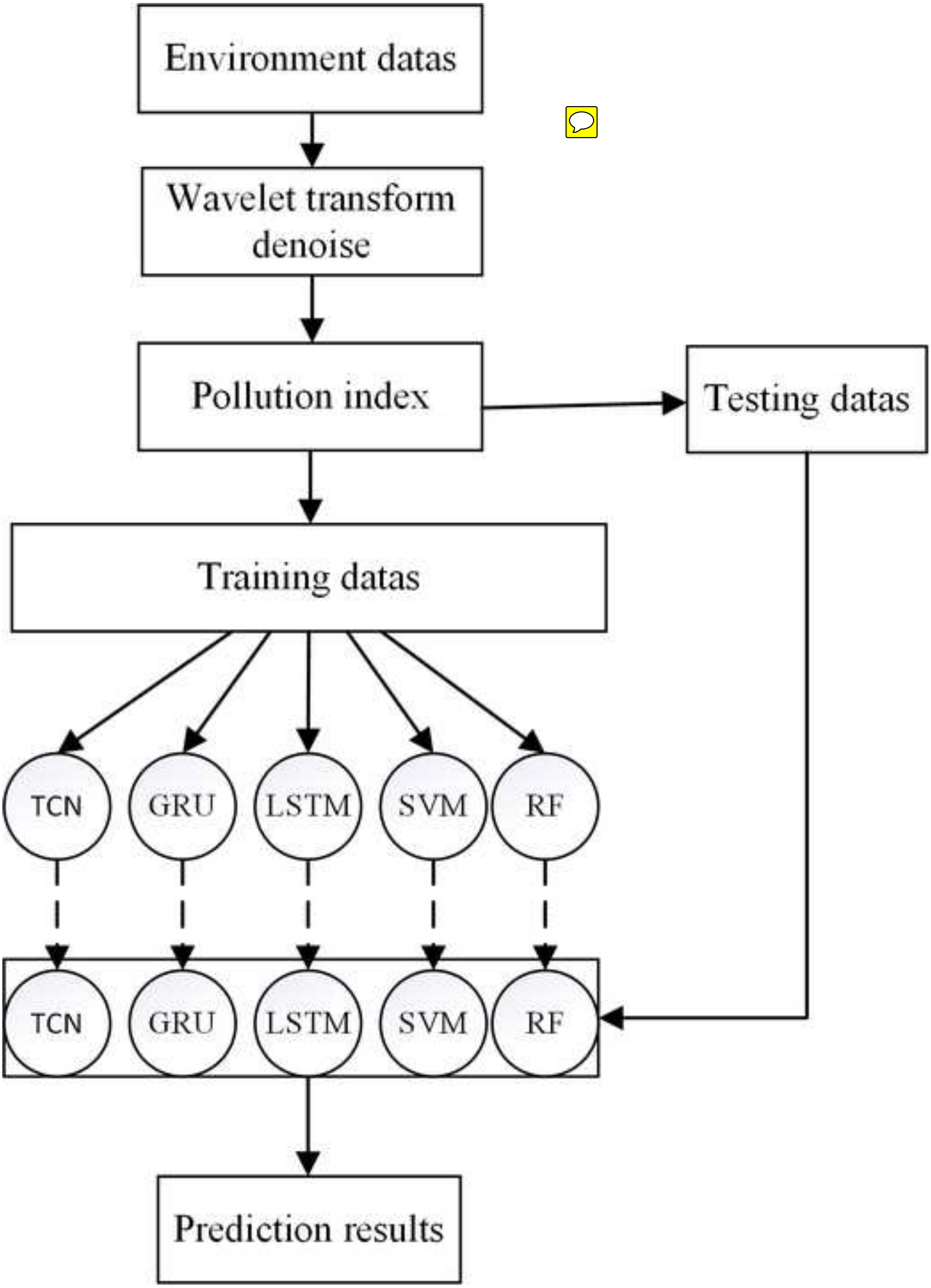

Fig6(a)

Click here to access/download;Figure;Fig6(a).tif

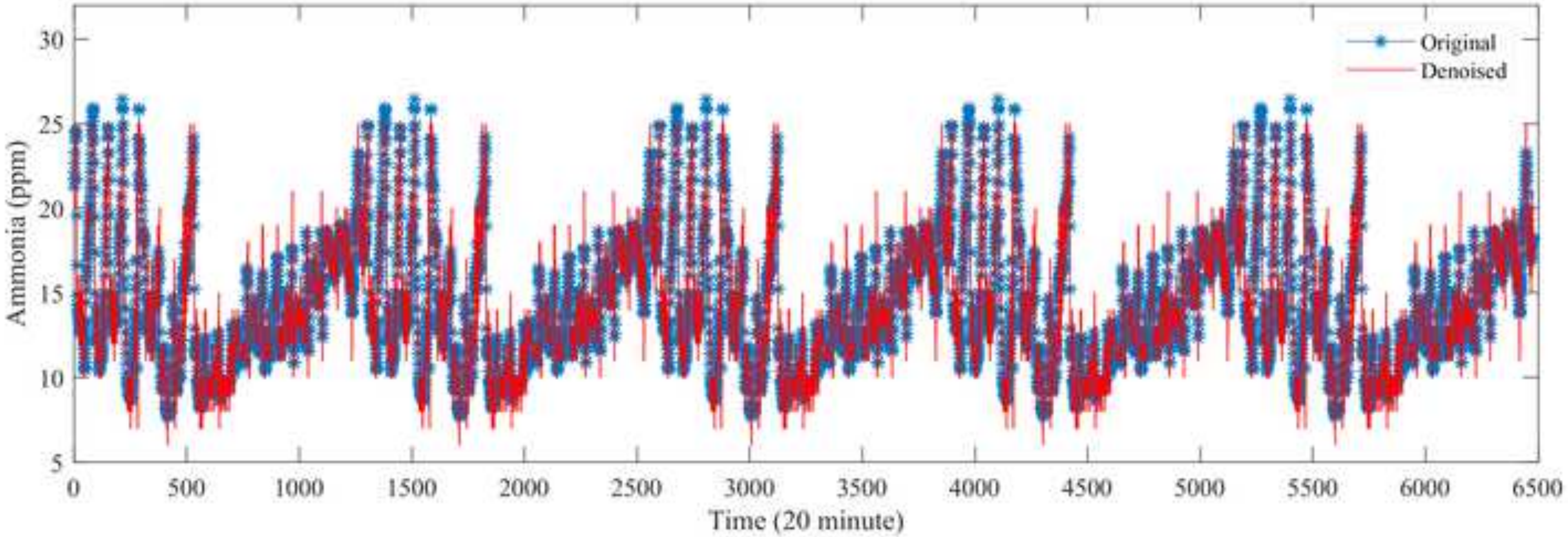

Fig6(b)

Click here to access/download;Figure;Fig6(b).tif

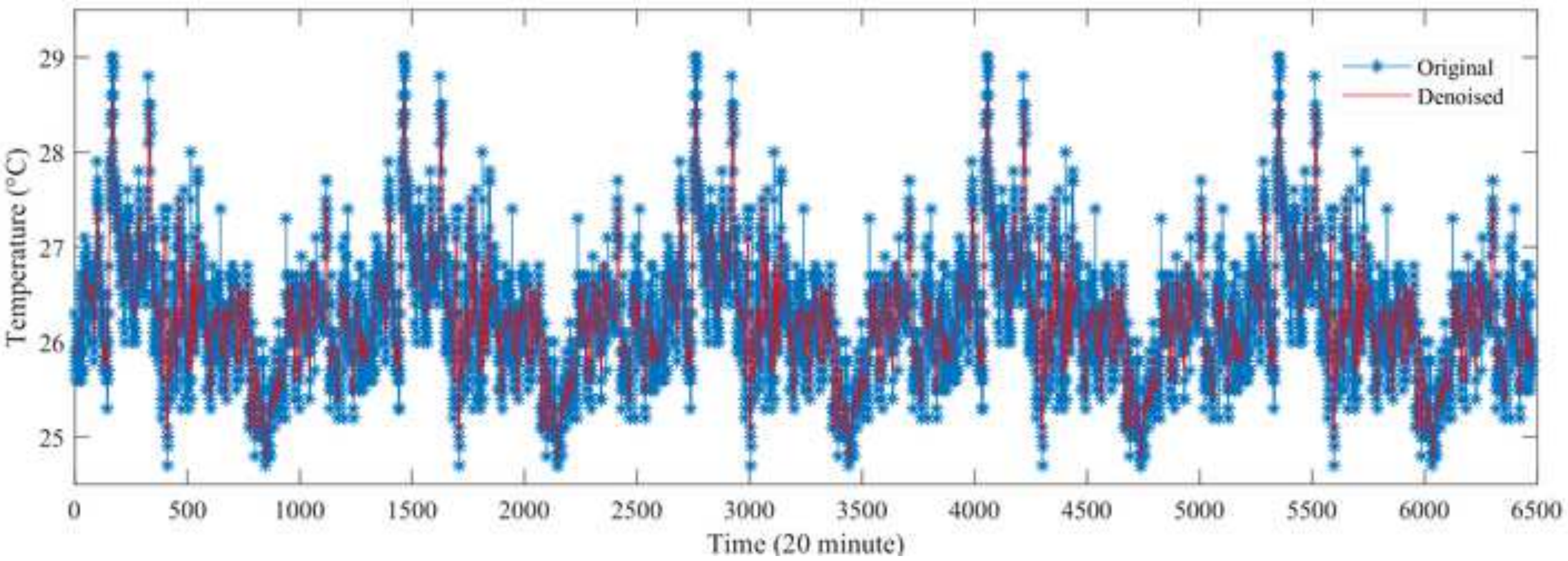

Fig6(c)

Click here to access/download;Figure;Fig6(c).tif

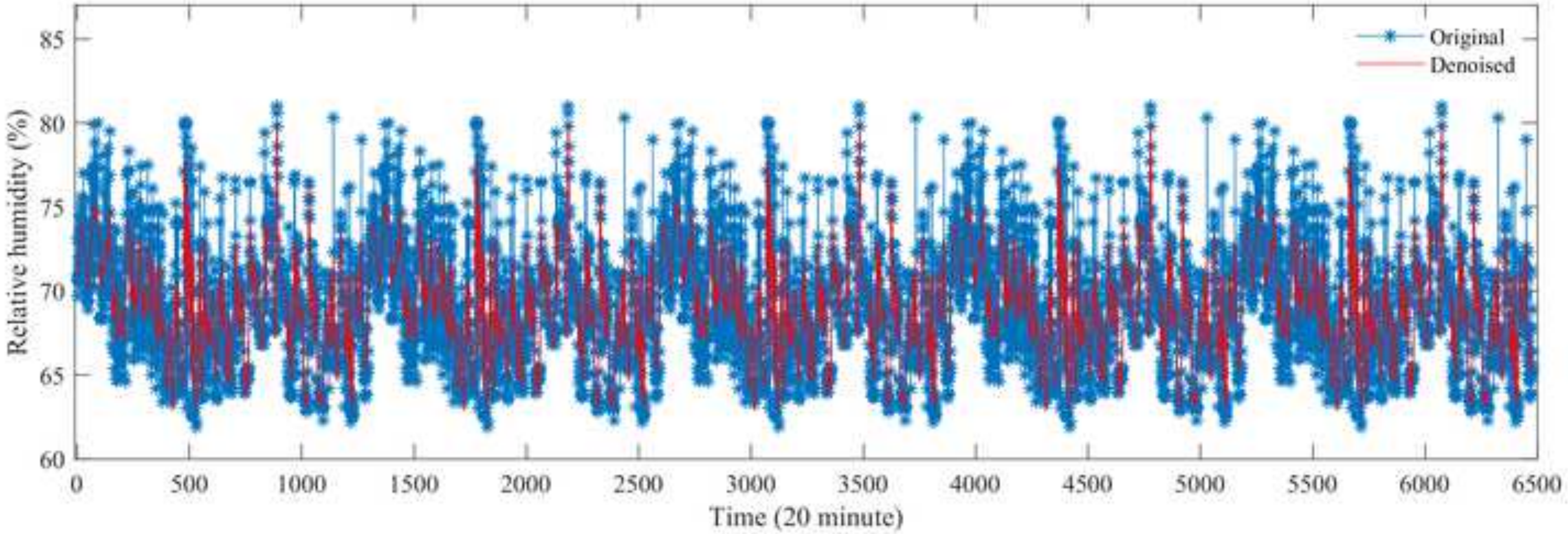

 Click here to access/download;Figure;Fig6(d) .tif

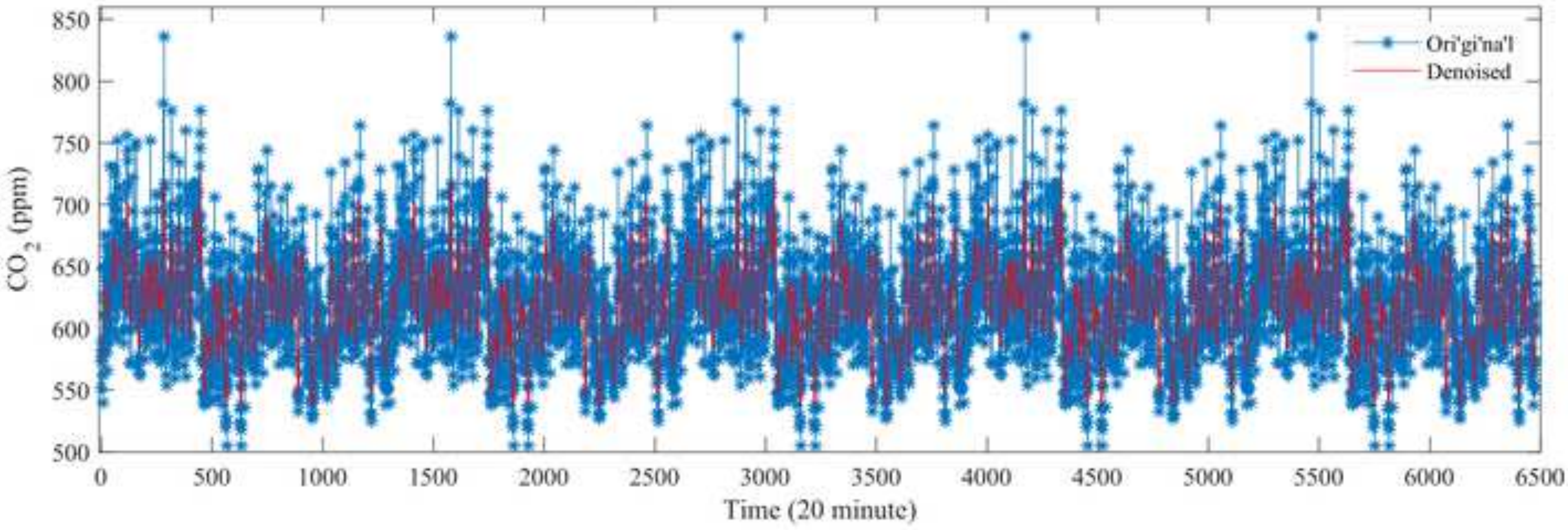

Fig6(e)

Click here to access/download;Figure;Fig6(e).tif

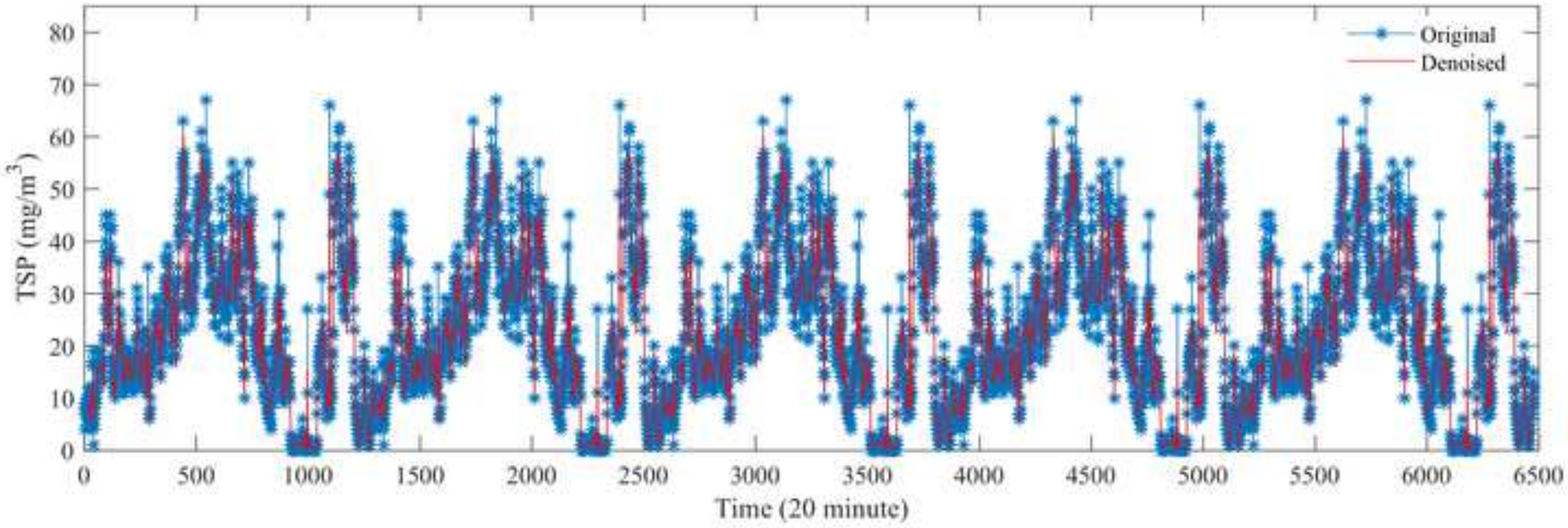

Fig7

Click here to access/download;Figure;Fig7.tif

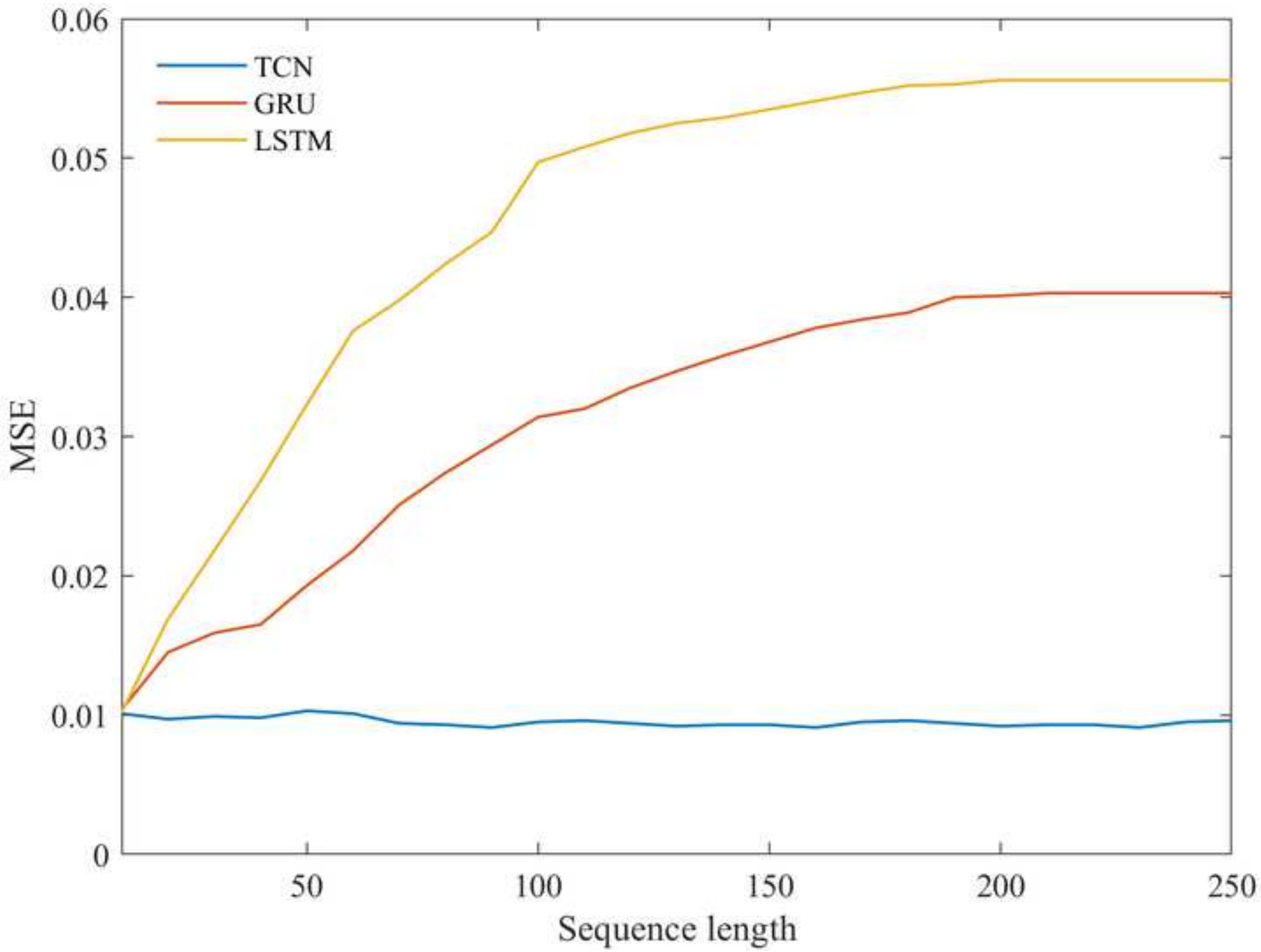

Fig8(a)

Click here to access/download;Figure;Fig8(a).tif

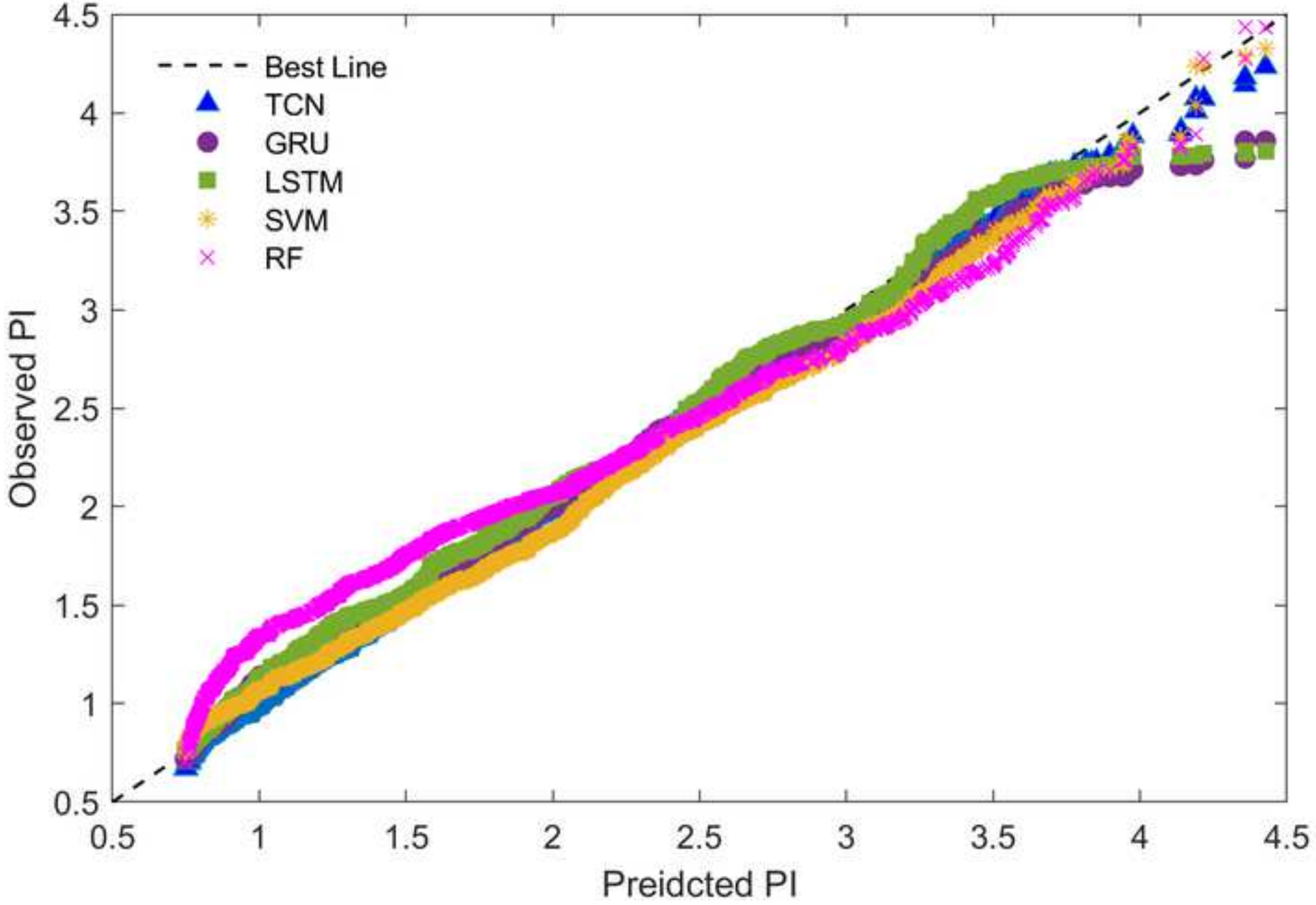

Fig8(b)

Click here to access/download;Figure;Fig8(b).tif

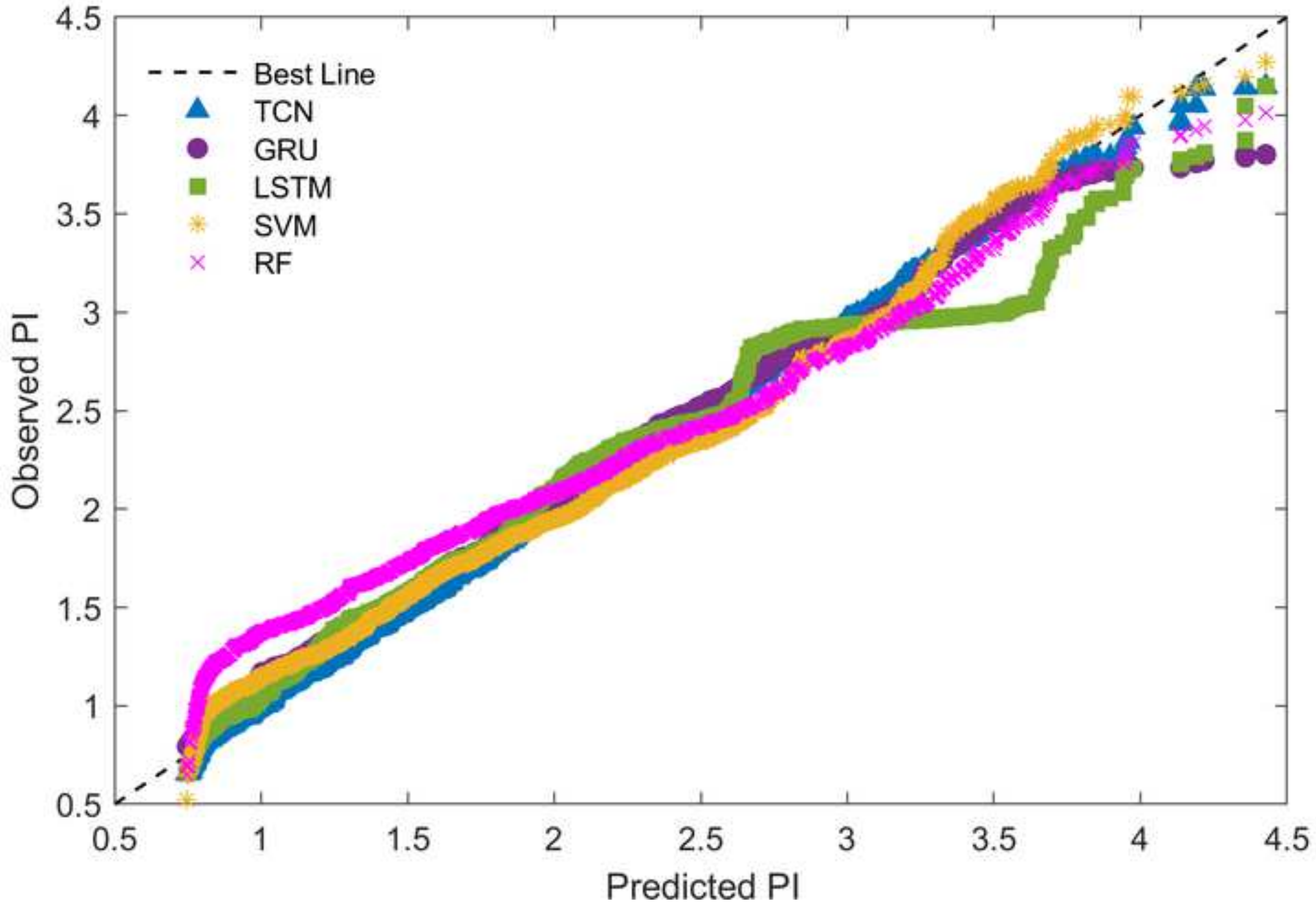

Fig8(c)

Click here to access/download;Figure;Fig8(c).tif

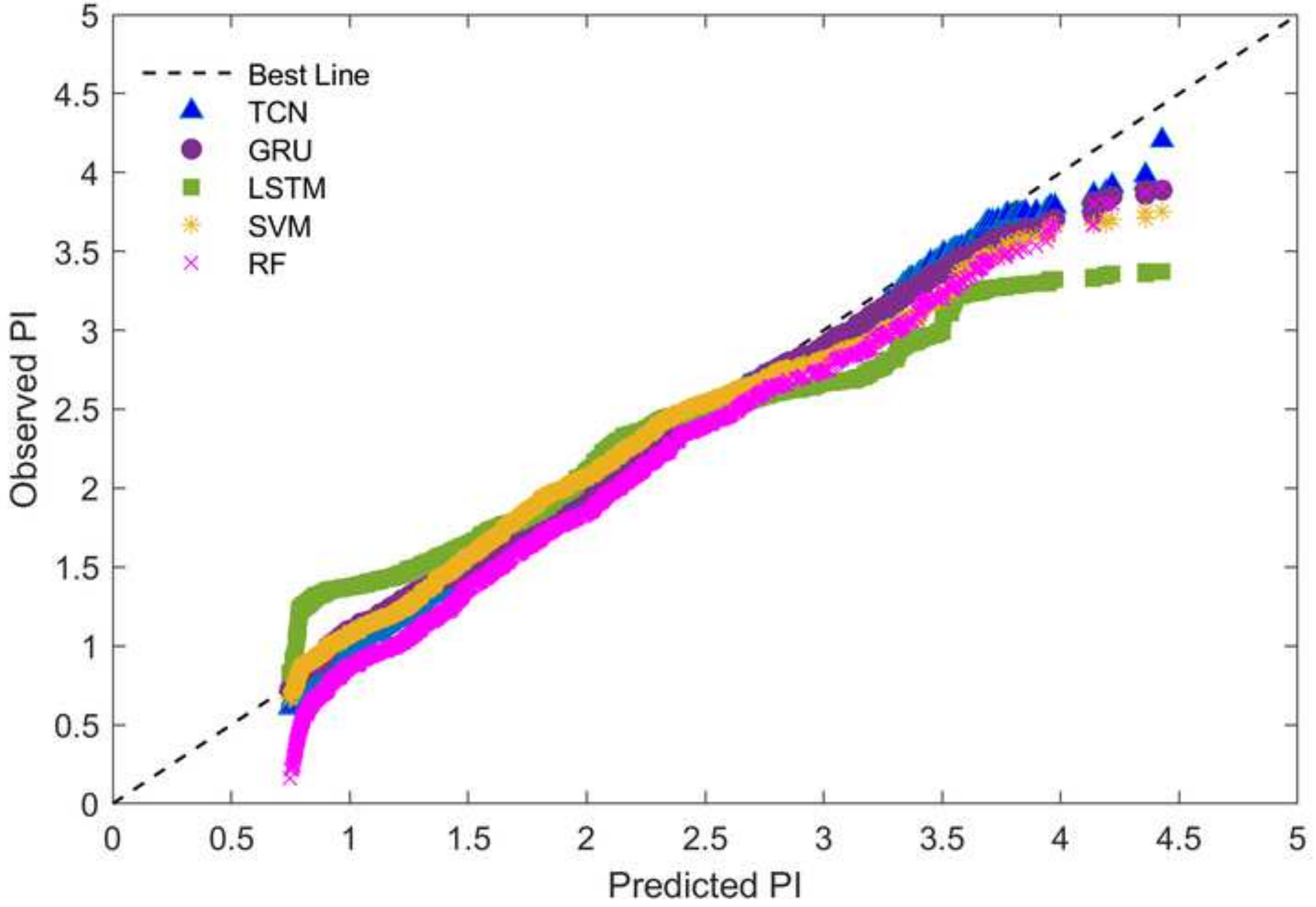

Fig9(a)

Click here to access/download;Figure;Fig9(a).tif

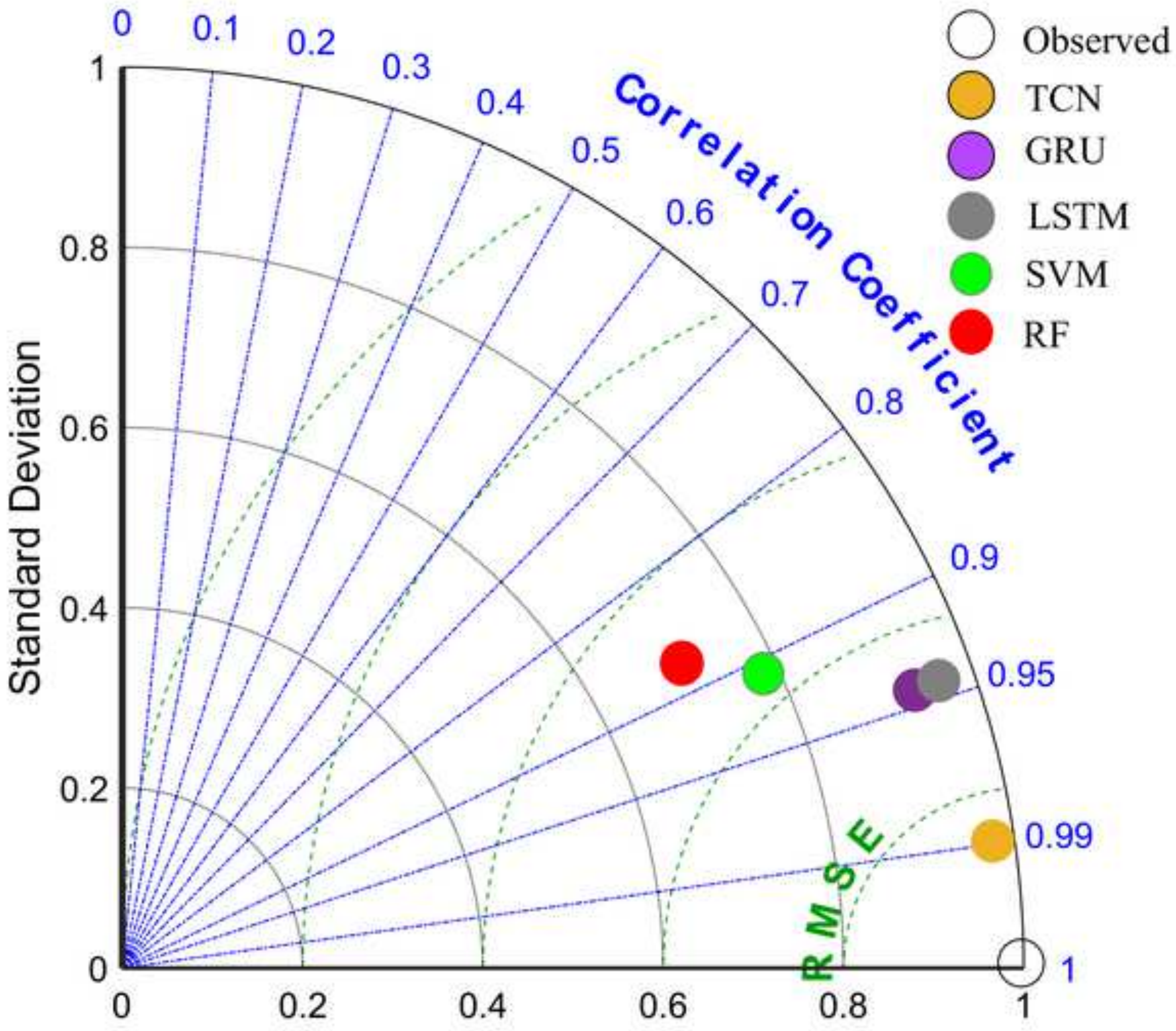

Fig9(b)

Click here to access/download;Figure;Fig9(b).tif

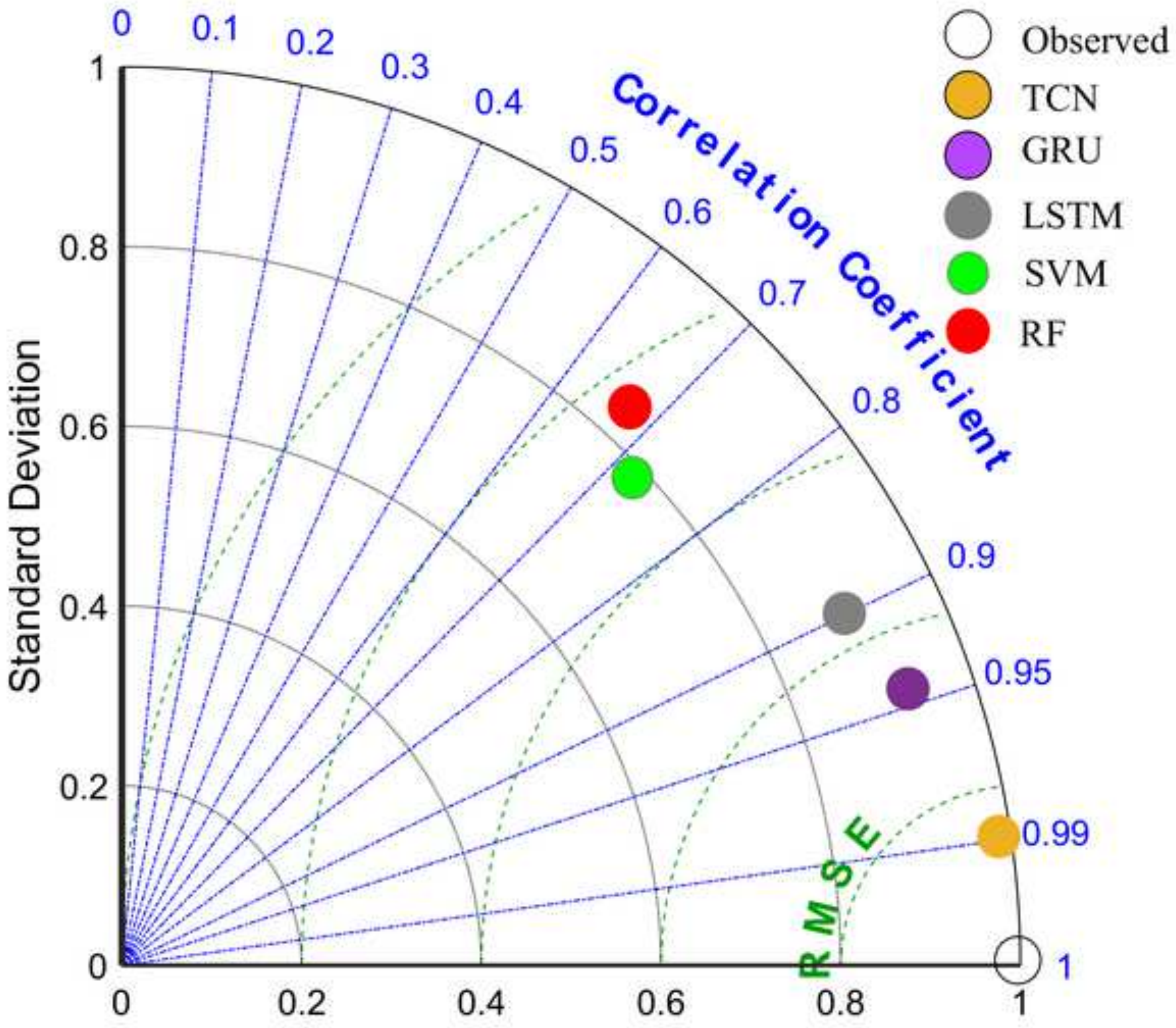

Fig9(c)

Click here to access/download;Figure;Fig9(c).tif

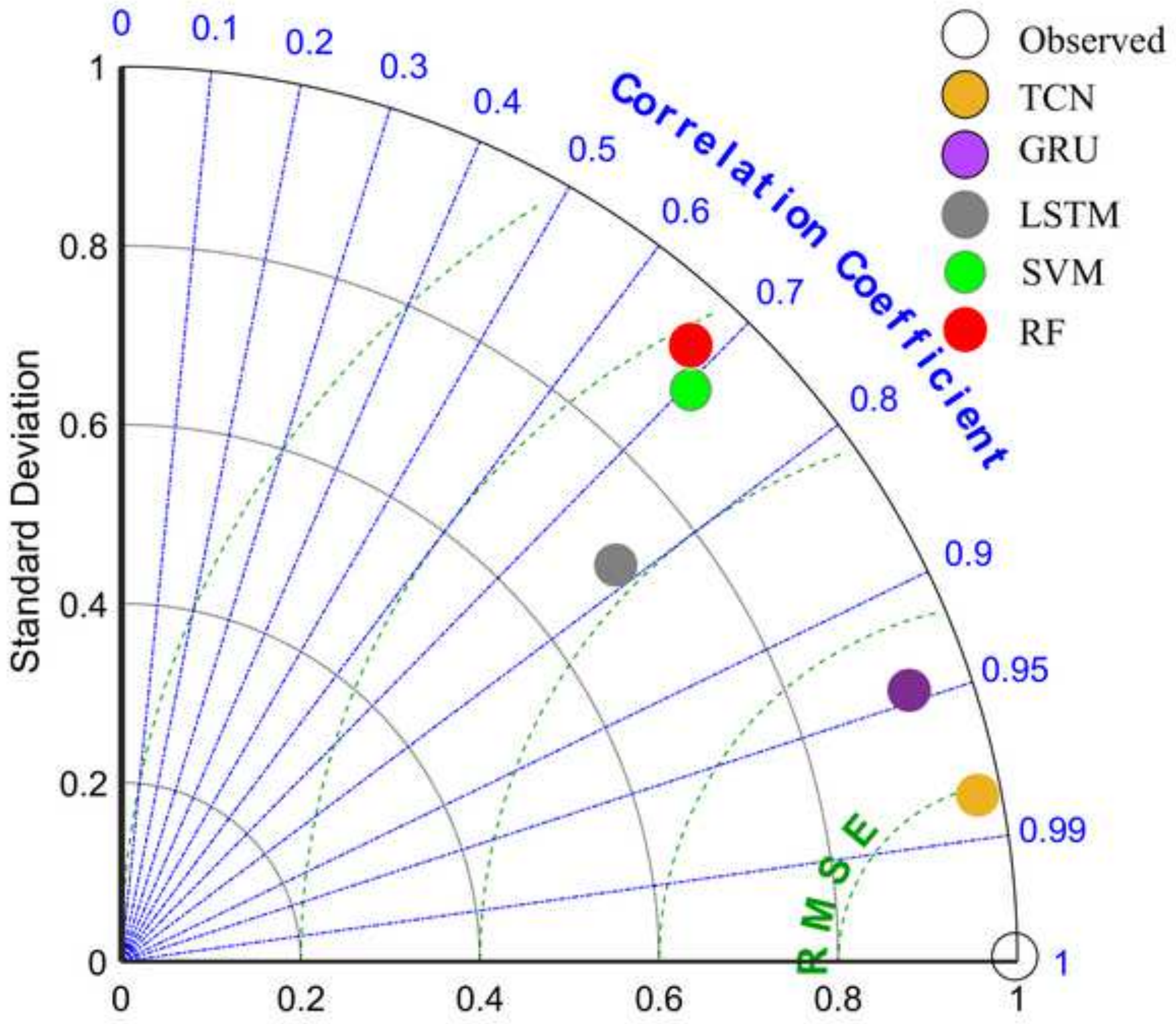

Fig10(a)

Click here to access/download;Figure;Fig10(a).tif

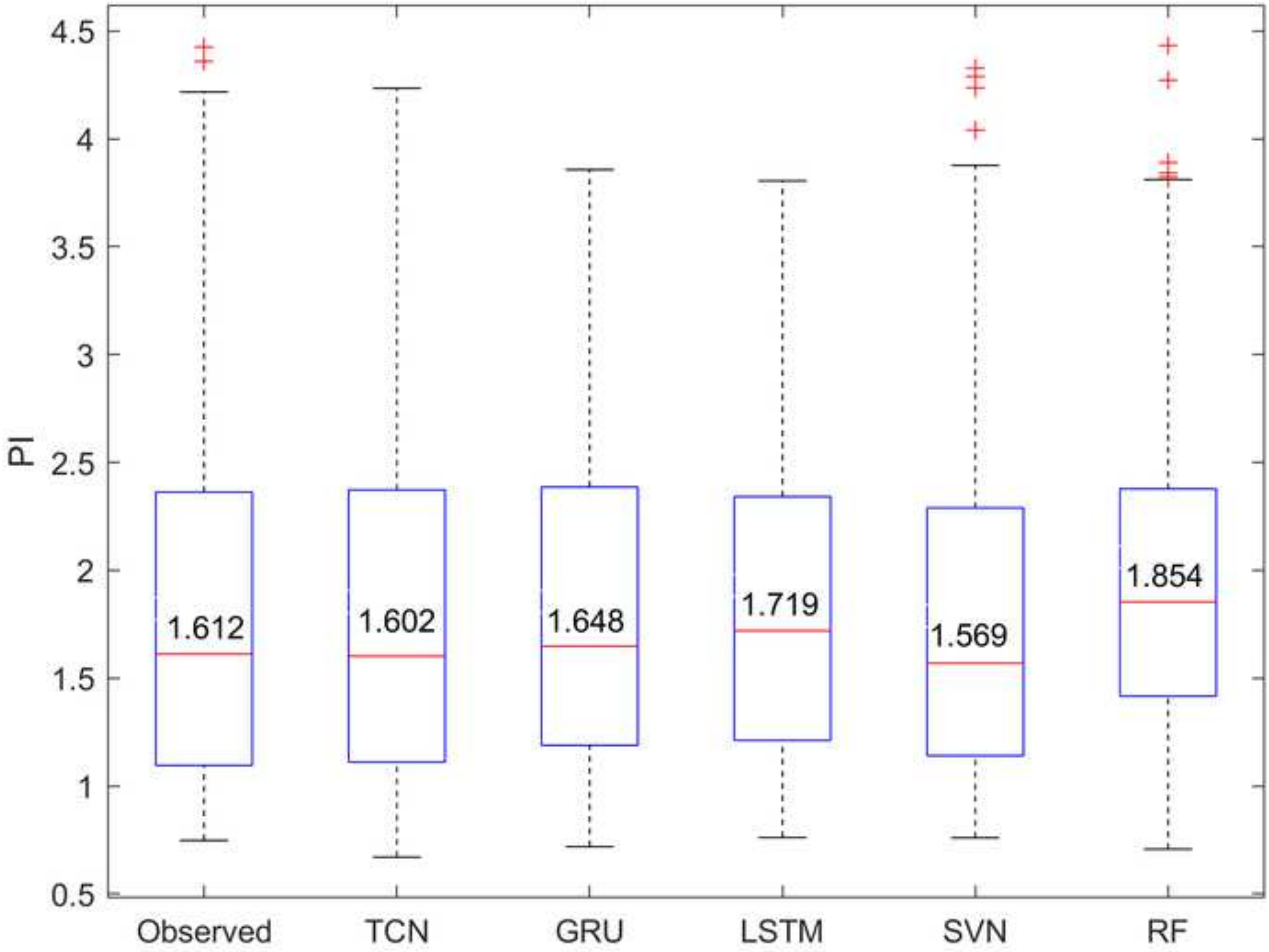

Fig10(b)

Click here to access/download;Figure;Fig10(b).tif

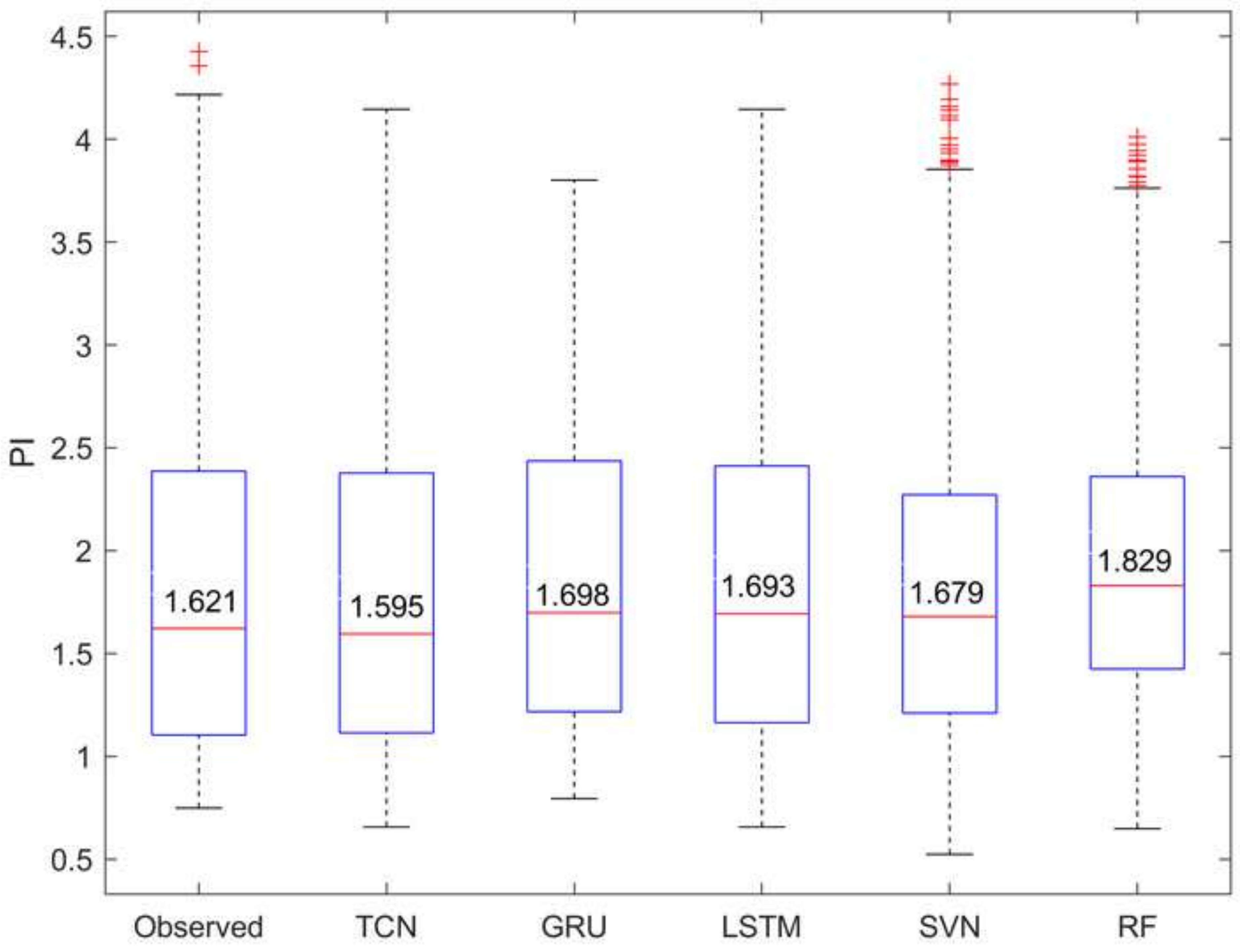

Fig10(c)

Click here to access/download;Figure;Fig10(c).tif

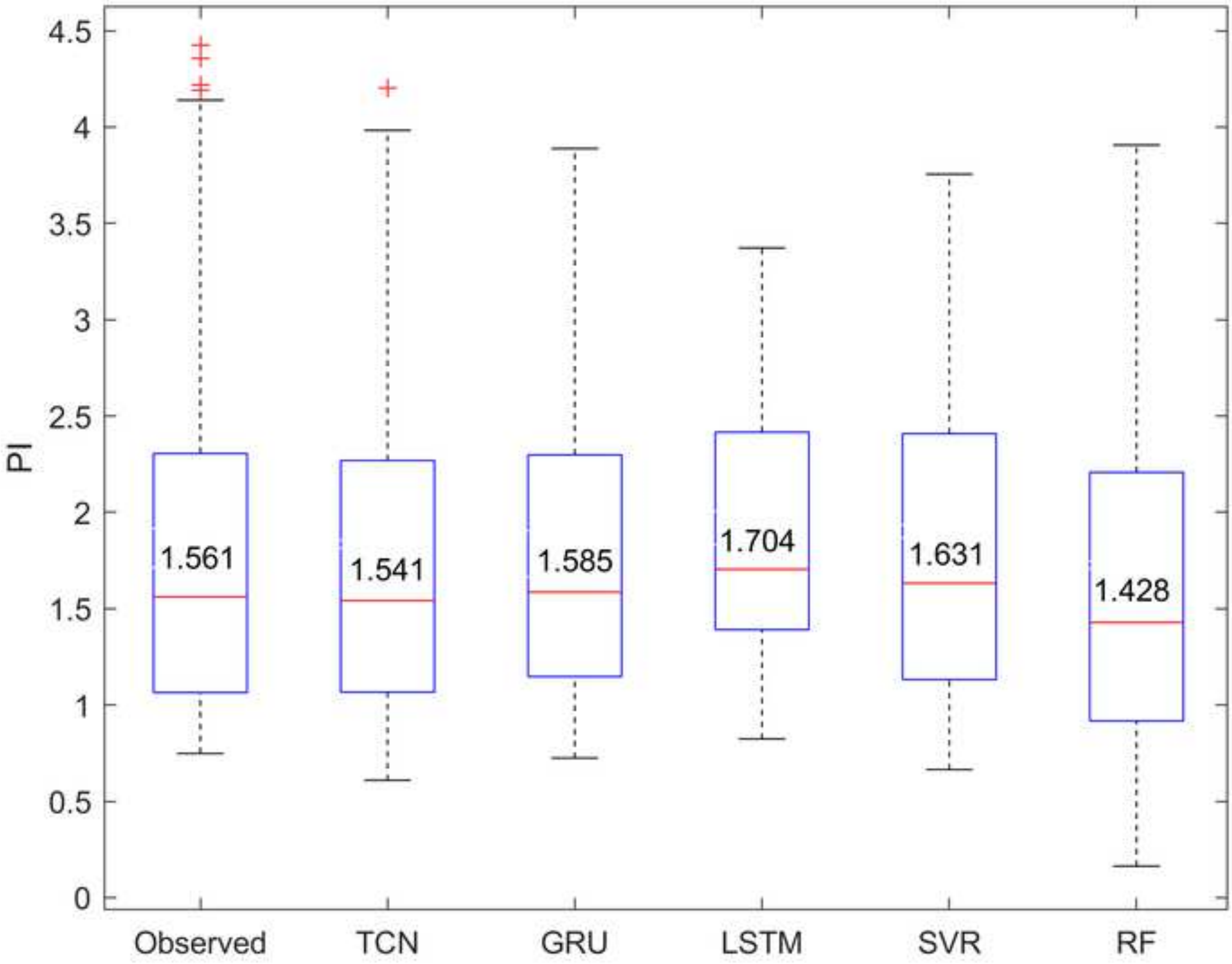

original data

Click here to access/download
Supporting Information
original data.xlsx

Revised Manuscript with Track Changes

Pollution index of waterfowl farm assessment and prediction based on temporal convoluted network

Jiande Huang[1,2,3,4,5,6], Shuangyin Liu[1,2,3,4,5,6,*], Shahbaz Gul Hassan[1,2,3,4,5,6, *],Longqin Xu[1,2,3,4,5,6]

[1]College of Information Science and Technology, Zhongkai University of Agriculture and Engineering, Guangzhou,510225, China

[2]Smart Agriculture Engineering Technology Research Center of Guangdong Higher Education Institues, Zhongkai University of Agriculture and Engineering, Guangzhou, 510225, China

[3]Guangzhou Key Laboratory of Agricultural Products Quality. Safety Traceability Information Technology, Zhongkai University of Agriculture and Engineering, Guangzhou 510225, China

[4]Guangdong Provincial Agricultural Products Safety Big Data Engineering Technology Research Center, Zhongkai University of Agriculture and Engineering, Guangzhou 510225, China

[5]Guangdong Province Key Laboratory of Waterfowl Healthy Breeding, Guangzhou 510225, China

[6]Academy of Smart Agricultural Engineering Innovations, Zhongkai University of Agriculture and Engineering, Guangzhou 510225, China

[*] Corresponding author.

[*] Authors contributed equallys.

E-mail address: shuangyinliu@zhku.edu.cn , mhasan387@zhku.edu.cn

[#a]Current Address: College of Information Science and Technology, Zhongkai University of Agriculture and Engineering, Guangzhou,510225, China.

Abstract

~~Environment~~Environmental quality is a major factor that directly impacts waterfowl productivity. Accurate prediction of PI is the key to improving environmental management and pollution control. This study applied a new neural network model called temporal convolutional network and a denoising algorithm called wavelet transform (WT) for predicting future 12~~-~~, 24~~-~~, and 48-~~hours~~hour PI values at a waterfowl farm in Shanwei, China. The TCN model performance was compared with that of ~~the~~ recurrent architectures with the same capacity, long-short time memory neural network (LSTM), and gated recurrent unit (GRU). Denoised environmental data, including ammonia, temperature, relative humidity, carbon dioxide (~~Co_2~~CO_2), and total suspended particles (TSP), were used ~~for constructing~~to construct the forecasting model. The simulation results showed that the TCN model in general produced a more precise PI prediction and provided the highest prediction accuracy for all phases (MAE=0.0842, 0.0859, and 0.1115;~~,~~ RMSE=0.0154, 0.0167, and 0.0273;~~,~~ R2=0.9789, 0.9791, and 0.9635). The PI assessment prediction model based on TCN exhibited the best prediction accuracy~~,~~ and general performance compared with other parallel forecasting models and is a suitable and useful tool for predicting PI in waterfowl farms.

~~Keyword~~Keywords: Agricultural informatization; environmental quality assessment; temporal convoluted network; regression prediction

Introduction

In response to the growing global demand for food, the Chinese waterfowl industry has grown to be a leader in both goose meat and goose egg production [1]. To meet this demand, one mechanism to increase production is to increase housing and manage more geese. However,

Commented [QCE2]: Abbreviations and acronyms are typically defined the first time the term is used within the abstract and again in the main text and then used throughout the remainder of the manuscript. Please consider adhering to this convention. The target journal may have a list of abbreviations that are considered common enough that they do not need to be defined.

as the scale of intensive culture increases, there is a growing concern~~,~~ in China~~,~~ that waterfowl should be raised under conditions that promote animal welfare since productivity is related to environmental conditions in which the waterfowl are raised [2]. For example, the organic matter present in excreta and/or litter resulting in ~~pollutants~~pollutant production~~ like~~, such as ~~Co2~~CO_2, ammonia, and TSP, will not only impair geese and staff health~~, there are also~~ but also have important consequences for society once the pollutants are out in the atmosphere [3–4]. According to this, to maintain and control optimal conditions for survival and good goose growth, establishing a habitat ~~to be~~that is closer to a standard ecosystem is justified [5].

Labor shortages and increasing biosecurity practices will make it more difficult for producers to monitor and manage the production, health, and welfare status of all of their birds. Employing modern poultry management technology is necessary to increase production [6]. An example of how modern management technology can be used to monitor and control the poultry house environment is exemplified by humidity regulation via ventilation rate changes mediated by relative humidity sensors, as relative humidity is one of the more important environmental aspects of a poultry house [7]. In addition, more advanced systems are being researched. Bustamante et al. used a multisensor system to effectively assess barn ventilation system function by tracking temperature, air velocity and differential pressure in broiler houses [8]. Hanif et al. proposed an internet of things technology-based protection and monitoring of the environment of a poultry house to monitor the environment-related parameters such as air temperature, air humidity, CO_2 level of concentration and ammonia concentration, which has been implemented successfully, leading to a safe environment and profit for the poultry industry [9]. The techniques mentioned above are both solutions for real-time environmental monitoring

and control; however, relying on hardware monitoring in real time cannot capture the trend of environmental changes [10], and it is easy to miss the best time for adjustment, which leads to waterfowl health damage and property loss.

Cultivation environment forecasting has been studied for many years and has made some achievements in aquaculture and livestock breeding [11–14]. The technique estimates or predicts the future changes in target variables that cannot be obtained directly. For example, Jackman et al. generated a prediction model by using sensor inputs of relative humidity, carbon dioxide, temperature, and ammonia for environmental parameter and crop yield prediction [15]. In waterfowl production, a system such as this would allow for actions to be taken sooner by farmers if the environment is projected to be bad. However, few studies have applied prediction in waterfowl breeding. Therefore, it is necessary to apply environmental prediction technology to waterfowl production to fill this gap.

~~Pollution~~The pollution index (PI) is a simple and easy assessment method for assessing ~~the environment~~ environmental quality, and ~~precision~~ precise ~~environment~~environmental quality assessment and prediction allow better ~~environment~~environmental management practices~~,~~ and contribute to a more sustainable ~~environment~~environmental management approach [16-17]. In recent years, many studies on pollution models have been carried out to evaluate the quality of the environment ~~base~~based on the PI method, such as ~~river's~~river quality status assessment and prediction [16,18–20], ~~air's~~air quality status assessment and prediction [21–23], and ~~soil's~~soil pollution assessment and prediction [24-25]. Forecasting the concentration of water, air, and soil pollutants is an effective method for protecting public health, productivity, and travel by providing early warnings of harmful pollutants. Likewise, it is also

important to assess and predict PI in advance for waterfowl farms so that, if necessary, the producer can intervene more quickly by using management practices ~~in order~~ to ensure a healthy context.

In general, PI formulations include lengthy ~~computation~~computations and thus require considerable time and effort. ~~Also~~Additionally, waterfowl environment pollutant data ~~is~~are dynamic, complex, and have high~~ly~~ temporal and spatial variability [26-27], and traditional ~~forecast~~forecasting methods such as multiple linear regression and autoregressive integrated moving average ~~model~~models have ~~bad~~poor performance. Hence, a method to calculate PI in an efficient and ~~precision~~precise way is required. Such approaches may benefit farmers when assessing and managing ~~environment~~environmental quality.

Over the past few decades, ~~Artificial Intelligence~~artificial intelligence has been increasingly applied to solve various environmental engineering problems, including water quality modeling [28-29]~~,~~ and air quality modeling [30]. Among ~~there~~them, STM is a class of neural networks~~, which~~ that can only use the current input information and historical information. Compared with other AI-based models, LSTM is powerful for modeling sequence data such as time series or natural language [31–33]. However, recursive neural networks such as LSTM and GRU process input sequences in parallel, so the cost of model training will increase with ~~the~~ increasing length of input sequences.~~,~~ Moreover, ~~the~~ distant historical memory will be forgotten, and they have ~~the~~ a weak ability ~~of~~ for temporal and spatial feature ~~extract~~extraction [34]. ~~So~~Therefore, ~~the~~ convolutional neural ~~network has~~networks have attracted ~~more and more~~ increasing attention in temporal and spatial sequence modeling. TCN [35], a model with a simple convolutional network architecture, is proposed to be applied to

language modeling and music modeling and has ~~obtained~~ demonstrated better performance than recurrent neural networks, especially in a long sequence. TCN has been ~~proved that performs~~proven to perform well in long sequence time series modeling. However, ~~TCN has~~TCNs have not yet been applied in PI prediction. Therefore, a better TCN prediction architecture is explored to promote the predicted precision of PI in this study.

Waterfowl house environment data usually contain many sources of noise. To eliminate noise interference, extract essential feature information, and obtain high-quality data ~~set~~sets, experts have proposed many methods~~,~~ such as WT, ~~Independent Component Analysis, and Empirical~~independent component analysis, and empirical mode decomposition [36–38]. Wavelet transform can decompose ~~the~~ time series with different resolutions~~,~~ and distinguish between noise and useful signals. It has been successfully applied to pattern recognition and noise elimination. Liu et al. proposed a hybrid wavelet analysis and least squares support vector regression with ~~cauchy~~a Cauchy partial swarm optimization algorithm model for dissolved oxygen prediction [11]. Kumar et al. obtained ~~the~~ better-quality ~~denoise Electrocardiogram~~denoised electrocardiogram signals by a denoising technique using a stationary wavelet transform compared with empirical mode decomposition, the Fourier decomposition method, and discrete wavelet transforms [39]. To effectively improve the GPR image resolution, Zhang et al. ~~combines the~~ combined WT and F-K filtering [40]. Samuel et al. ~~marked~~ conducted a comparative analysis of a set of machine learning models, and the results ~~shown~~showed that the best combination for predicting the streamflow into the Sobradinho Reservoir was the bootstrap, WT and neural network [41].

Based on the above studies, this paper proposes a new waterfowl house environmental

quality assessment and prediction model combining wavelet transform and a temporal convolutional network. The wavelet transform can reduce the noise of original ~~environment~~environmental data and obtain high-quality data, and the temporal convolutional network~~s~~ can extract the temporal and spatial features of processed data, output ~~precision~~ precise environmental quality assessment, and ~~prediction~~predict results.

Materials and Methods

Study area and data source

The waterfowl culture farm in Haifeng County (23 °05’N, 115 °19’E) in Shanwei ~~city~~City, China was ~~considered for~~ investigated in the present study. With an area of approximately 53.3 hm2, the farm ~~was~~ is a multifunctional integrated ~~aquaculture~~ aquacultural base~~d~~ integrating waterfowl breeding, seeding breeding and intensive aquaculture.

The ~~environment~~environmental data ~~was~~were collected by the waterfowl culture internet of thing (IoT) online monitoring terminal system, and its architecture is shown in Fig. 1. The system ~~was~~is ~~based latest~~ equipped with a temperature sensor, ammonia concentration sensor, humidity sensor, etc. The ~~environment~~environmental information ~~would be upload~~ is uploaded to the cloud, and users can obtain ~~the~~ on-site information by accessing the web server through mobile ~~phone~~phones or personal ~~computer~~computers.

Fig. 1~~Fig.1~~ Schematic diagram of the ~~the~~ waterfowl farm monitoring system based on the ~~Internet~~ internet of ~~Things~~things.

According to the importance of the environment and ~~the researches of experts, we have~~ expert research, we selected 5 environmental factors, as shown in Table~~.~~ 1. Among them, ammonia is a toxic gas and the greatest concern of environmental pollution in waterfowl

production, adversely affecting the ecosystem, environment, and health of birds and people. Less than 10 ppm is the ideal limit [42]. Relative humidity can impact bird health, and high relative humidity may worsen broiler geese performance; the ideal value is 70% [43]. Heat stress is a major concern in waterfowl production; high and low temperatures will reduce the growth performance and survivability of waterfowl, and temperatures less than 27°C and more than 10°C are ideal [44]. TSP is from the birds themselves as well as from the feed, litter, and building materials and may serve as a pathogen disseminator and bring about lung damage; less than 10 mg/m^3 is the ideal limit [42]. Additionally, the levels of relative humidity, ammonia, carbon dioxide, temperature and TSP are known to be correlated with each other [6]. These factors are important for waterfowl production and can be monitored and optimized.

Table 1Table.1 The selected environmentenvironmental parameters for PI prediction.

Environmental factor	Unit	Limit	Influences
Ammonia	ppm	10	Ammonia is a toxic gas impairing animal performance and bird and staff health [41-42].
Relative humidity	%	70	High relative humidity may worsen broiler geese performance [43].
Temperature	°C	10~27	Influences poultry welfare and food intake, as well as increases susceptibility to disease and flock mortality rate [44].
$Co_2$$CO_2$	ppm	1500	A decrease in production and bird health can occur [45].
TSP	mg/m^3	8	A higher incidence of lung damage [42].

Data sets

In this report, environmentenvironmental data included, including ammonia, relative humidity, temperature, Co_2 CO_2 and TSP, were collected from July 1^{stth} to September 28th, 2019, at intervals of 20 minutes. There were 72 sets of data collected per day with yield of totala total yield of 6480 observed samples. For a model generation, Thethe first 4536 sets of the data were

used for model training, and the remaining 1944 sets were used as the testing data to estimate the prediction performance of the constructed model.

Pollution index

~~This study using t~~The ~~formulation of~~ pollution index in this study~~evaluated~~ was formulated within the criteria for evaluating the environmental quality of ~~the~~ livestock and waterfowl ~~farm~~farms by the ~~quality~~ Quality and ~~technology~~ Technology ~~supervision~~ Supervision ~~bureau~~ Bureau of ~~china, the~~China. The single pollution index can be calculated as:

$$I = {C_i}/{C_{si}} \tag{1}$$

~~Where~~where C_i is the measured concentration of environmental pollutants~~,~~ and C_{si} is the standard concentration ~~limits~~limit of pollutants. ~~And the~~ The composite pollution index of waterfowl house environment quality can be described as ~~follow~~follows:

$$P_i = \left\{ \mathrm{I}_{\max} \cdot (1/n) \sum I_i \right\}^{1/2} \tag{2}$$

~~Where~~where I_{max} is the ~~max~~maximum single pollution index among all pollutants, n is the number of pollutants, and I_i is the single pollution index of pollutant i.

Wavelet transform

Continuous wavelet transforms for a given waterfowl environment signal $s(k)$ can be described as follows:

$$WTs(a,b) = \frac{1}{\sqrt{a}} \int_{\infty}^{-\infty} s(k) \overline{\left(\psi\left(\frac{t-b}{a} \right) \right)} dx \tag{3}$$

~~Where~~where $\overline{(\ )}$ ~~denoted~~denotes the complex conjugate, $\psi(x)$ is the wavelet function, a is the time scale dilation and b is the time translation. By controlling the ~~value~~values of ~~parameter~~parameters a and b ~~can achieve the~~ , signal time-frequency positioning~~. Wavelet~~ can

be achieved. The wavelet transform can decompose signalsignals into multi-resolution multiple resolutions. However, the symbols in realization communication are discrete data, so the continuous wavelet transform needs to be discretized. Suppose $a = a_0^j, b = kb_0 a_0^j, j,k \in Z$, when $a_0 = 2, b_0 = 1$:

$$\psi_{j,k}(n) = 2^{-j/2}\psi(2^{-j}n - k) \tag{4}$$

The discrete waterfowl environment signal s(n) can be transformed as:

$$D(j,k) = \sum_k s(n)\overline{\psi_{j,k}}(n) \tag{5}$$

The Mallat algorithm can quickly calculate the orthogonal wavelet transform coefficients and realize signal decomposition and reconstruction [47]. The approximation coefficients $s_{j+1}(n)$ and detail coefficients $d_{j+1}(n)$ can be recurrently relaterrelated by the Mallat algorithm as followfollows:

$$s_{j+1}(k) = \sum_n h_0(n-2k)s_j(n) \tag{6}$$

$$d_{j+1}(k) = \sum_n h_1(n-2k)s_j(n) \tag{7}$$

Wherewhere h_0 and h_1 are the high-pass filter and low-pass filter, respectively. The reconstruction is the inverse process of decomposition:

$$s_j(k) = \sum_n s_{j+1}(n)h_0(k-2n) + \sum_n d_{j+1}(n)h_1(k-2n) \tag{8}$$

WaveletThe wavelet transform can decompose noise signalsignals into different signal channels at various levels of resolution. In general, the useful signals are distributed in the high -level resolution channel, and the noise signal wasis likely to be distributed in the lower levels one-level channel. Therefore, the noise of the original data can be reduced as much as possible

~~base~~based ~~on the~~a ~~reasonable threshold. So~~Therefore~~, the wavelet denoised algorithm can be described as follows:~~

~~(1) Perform wavelet transform on the original signal *s(k)* to obtain wavelet coefficients of various scales.~~

~~(2) Determine the threshold and perform threshold denoise~~denoising ~~on different wavelet coefficients to obtain estimated wavelet coefficients.~~

~~(3)~~ Conduct wWavelet ~~reconstruction by equation. 11 to obtain the denoise signal~~ $\widehat{s(k)}$ ~~.~~

Support vector machine

SVM has good generalization ability in solving ~~non-linear~~nonlinear, small sample, and high-dimensional pattern recognition, and the optimal solution obtained is global, which solves the local optimal problem that cannot be avoided in other algorithms. The prediction process of the SVM includes ~~the~~ support vector determination, kernel function selection, kernel parameter determination, and solution. ~~The mathematical expression as follow:~~

$$N_t = \sum_{SVS} \alpha_i y_i K(x_i, x) + b_0 \quad (9)$$

~~Where *SVS* is the number of support vectors, α_i is the Lagrangian coefficient of each training sample, y_i (-1 or 1) is the vector label, $K(x_i, x)$ is the selected kernel function, and b_0 is the bias. The kernel function replaces the linear quantity in the traditional linear equation and maps the data to high-dimensional space processing. After selected the kernel function, the samples are trained to establish the SVM model and the prediction result *Nt* is finally obtained.~~

Random forest

RF is a ~~non-linear~~nonlinear ensemble model that establishes and averages a large number of

random distribution decision ~~tree~~trees for regression or classification tasks [48]. A decision tree or classification and regression tree that ~~construct~~constructs the RF is a ~~non-parametric~~nonparametric model. According to the complexity of the input data, the tree grows in the process of ~~the~~ learning. Decision nodes and leaf nodes are the main components of the decision tree. Each input sample is estimated by a test function of decision nodes and passed to different branches according to the features of the sample. After all trees are trained, each tree can predict the test sample set according to the node threshold, and the results of each tree are combined to vote to determine the final result of the entire random forest.

Long short-term memory neural network~~.~~

The LSTM neural network is a special kind of recurrent neural network. It was first proposed by Ho-chreiter and Schmidhuber [49]. Its appearance effectively solved the gradient explosion problem of traditional recurrent neural ~~network~~networks. At the same time, the LSTM neural network has long-term memory to handle long-term sequence data. ~~The LSTM cell structure consists of forget gate, input gate, output gate and cell state. LSTM structure is shown in Fig 2 At the time t, there are three input parameters of the LSTM network: input value x_t, at time t, output value h_{t-1} and cell state C_{t-1} at $t-1$. There are two output parameters of the LSTM network: output value h_t and cell state C_t at time t. Through the activation function σ LSTM realizes the control of the three gates so as to realize the retention and forgetting of historical information.~~

~~Fig.2. The structure of LSTM~~

Gated recurrent unit~~.~~

A gated recurrent unit (GRU) is a type of recurrent neural network (RNN). Similar to a long

short-term memory neural network (LSTM), it is also proposed to solve long-term and gradient backpropagation problems. LSTM and ~~a~~ GRU have similar performances, but compared with LSTM, ~~the~~ GRU is computationally cheaper. ~~The structure of GRU is shown in Fig 3 GRU can be descried as follows:~~

~~Firstly, two output gateds, *r* (reset gated) and *z* (update gated), were obtained by the input *X(t)* at *t* time and the output *h(t − 1)* at *t − 1* time.~~

$$r = \sigma(W_r \cdot [h(t-1), X(t)]) \tag{10}$$

$$z = \sigma(W_z \cdot [h(t-1), X(t)]) \tag{11}$$

~~Where σ CJ is a sigmoid function. Parameter *h'* selective re- members *X(t)*. Trough the reset gated *r* we can obtain *h'* as follows:~~

$$h' = \tanh(W_h \cdot [r * h(t-1), X(t)]) \tag{12}$$

~~Finally, the output *y(t)* and hidden state *h(t)* at t time can be calculated as follows:~~

$$h(t) = (1-z) * h(t-1) + z * h' \tag{13}$$

$$y(t) = \sigma(W' \cdot h(t)) \tag{14}$$

~~Where [ ] denoted the connection of two vectors and * denoted the product of matrices.~~

~~**Fig.3. The structure of GRU**~~

Temporal convoluted network~~.~~

TCN, like~~s~~ LSTM, is a novel neural network architecture that can be used ~~to~~for time series prediction. The outstanding advantage of ~~TCN was~~ TCNs is that they not only have much longer memory~~,~~ but also have higher computational efficiency ~~compared with LSTM and others~~than LSTM and other recurrent neural ~~network~~networks [50].

In general, a nature sequence modeling task is any function $f: X^{T+1} \rightarrow Y^{T+1}$ that produces the mapping:

$$y_0, y_1, \ldots, y_T = f(x_0, x_1, \ldots, x_t) \tag{9}$$

The goal of the sequence model is to fit this function *f* to minimize the expected loss. It ~~is~~ satisfies the causal constraint that y_t depends only on $x_0, x_1, \ldots, x_t$ and not on future inputs x_{t+1} and that the output has the same length as the input.

As ~~show as~~shown in Fig. 2, to ~~satisfies~~satisfy the causal constraint, TCN uses a 1D fully~~-~~ convolutional network ~~(FCN)~~ architecture [51], which is different~~ with~~ from the traditional convoluted neural network in that the value at time *t* only depends on the value at time *t* and before in the previous layer. ~~And~~In addition, zero padding of length (kernel size - 1) is added to keep subsequent layers the same length as previous ~~ones~~layers.

Fig. 2~~Fig.2~~ Causal convolution construction.

One of~~ goal for TCN~~ the goals of TCNs is a long effective history size, ~~that~~ which ~~mean~~means an extremely deep network or very large filters. However, more convolution layers or larger filters bring about the problems of disappearing gradients, complex training, and poor fitting effects. To solve the problems above, dilated convolutions [35] ~~was~~were employed in the TCN. Specifically, given a sequence input $X^{n+1} = \{x_0, x_1, \ldots, x_n\}$ and convolution function $f: \{0, 1, \ldots, k-1\} \rightarrow \mathbb{R}$, the dilated convolution operation ~~were~~was defined as follows:

$$F(s) = \sum_{i=0}^{k-1} f(i) \cdot x_{s-i \cdot d} \tag{10}$$

~~Where~~where *k* is the kernel size, *d* is the dilated factor, and $s - i \cdot d$ accounts for the direction

of the past. When *d=1*, a dilated convolution is equal to a regular convolution. As shown ~~as~~in Fig. 3, as the number of layers increases, the dilated factor *d* grows, and the top layer can represent a wider range of ~~input~~inputs. On the other ~~way~~hand, choosing a larger kernel size *k* of the filter can also effectively ~~expanding~~expand the receptive field of a ConvNet.

Fig. 3~~Fig.3~~ Dilated ~~Convolutions~~ convolution construction.

Residual connections proved to be an effective method for deep network training to converge quickly and reduce the risk of overfitting [52]. As shown ~~as~~in Fig. 4, the residual block used in the TCN has two branches from input to output. The first branch contains a series convolution layer, parameter regularization, rectified linear unit, and dropout layer in order. This was a flexible architecture that allows the layer to modify ~~the~~ parameters such as the activation function~~,~~ and dropout rate~~, etc~~. The second branch ensures that the output sequence length is equal to the length of the input: if the lengths of the input sequence and output sequence ~~were~~are equal, the output layer is connected to the input layer ~~trough~~through identity mapping~~,~~ ~~otherwise~~; otherwise, the output layer ~~was~~is connected to the input layer ~~trough~~ through a ~~1x1~~ 1×1 convolution.

Fig~~.~~. 4 An example of residual connection in a TCN.

Processing of environmental data

Different ~~environment~~environmental factors may contain different ~~kind~~kinds of noise~~,~~. ~~to~~To achieve the best effect of ~~denoise~~ the denoising process~~,~~ and obtain quality data, we adopt four wavelet ~~function include~~ functions, Db4, Haar, Coif, and Sym10, to process each ~~environment data, respectively. Signal~~environmental dataset. The signal-noise ratio (SNR) and normalized cross correlation (NCC) were used to evaluate the denoised effect, which can be

described as:

$$SNR = \frac{I_1^2}{(I_2 - I_1)^2} \tag{11}$$

$$NCC = \frac{\sum (I_1 - mean(I_1)) \cdot (I_2 - mean(I_2))}{\sqrt{\sum (I_1 - mean(I_1))^2 \times \sum (I_2 - mean(I_2))^2}} \tag{12}$$

Wherewhere I_1 is the denoised signal and I_2 is the original signal; theoretically, the larger the SNR and NCC are, the better the noise reduction effect, theoretically. As shown as in the Table.Table 2, wavelet function Db4 was suitable for ammonia data (SNR=9.4318, NCC=0.9485), Sym10 was suitable for temperature data (SNR=7.4635, NCC=0.9221), relative humidity data (SNR=4.0809, NCC=0.8630), and Co_2 CO_2 data (SNR=1.7785, NCC=0.7793), and Coif was suitable for TSP data (SNR=13.1443, NCC=0.9771). The best denoised results of each environment data was showed asenvironmental dataset are shown in Fig. 5.

Table 2Table.2 Denoised performance of five parameters.

Target	Performance criteria	Wavelet function			
		Db4	Haar	Coif	Sym10
Ammonia	SNR/(dB)	9.4318	8.8287	9.3769	9.3602
	NCC	0.9485	0.9432	0.9484	0.9474
Temperature	SNR/(dB)	7.2491	6.5372	7.0205	7.4635
	NCC	0.9192	0.9061	0.9149	0.9221
Relative humidity	SNR/(dB)	3.9422	3.6395	3.8920	4.0809
	NCC	0.8600	0.8559	0.8596	0.8630
$Co_2$$CO_2$	SNR/(dB)	1.7064	1.3855	1.4991	1.7785
	NCC	0.7764	0.7642	0.7689	0.7793

TSP	SNR/(dB)	12.8848	12.6578	13.1443	13.1142
	NCC	0.9756	0.9745	0.9771	0.9769

(a) Ammonia sequence.

(b) Temperature sequence.

(c) Relative humidity sequence.

(d) ~~Co2~~ CO_2 sequence.

(e) TSP sequence.

Fig. 5~~Fig.5~~ Results of noise reduction of five parameters by WT.

Performance criteria

In this paper, the mean absolute error (MAE), root mean square error (RMSE) and coefficient of determination (R^2) were selected to measure the prediction accuracy and operation efficiency, and MAE, RMSE and R^2 are defined as follows:

$$MAE=\frac{1}{N}\sum_{i=1}^{N}|y_i-y_i'| \tag{13}$$

$$RMSE=\sqrt{\frac{1}{N}\sum_{i=1}^{N}(y_i-y_i')^2} \tag{14}$$

$$R^2=\frac{\left(\sum_{i=1}^{N}(y_i-\overline{y})(y_i'-\overline{y}')\right)^2}{\sum_{i=1}^{N}(y_i-\overline{y})^2\cdot\sum_{i=1}^{N}(y_i'-\overline{y}')^2} \tag{15}$$

~~Where~~where N is the total number of actual points in the data, yi is the observed value of period i, y_i is the prediction value of period i, y is the average of observed values, and y_t is the average of prediction values.

The waterfowl house environment quality assessment and

prediction model~~.~~

The algorithms used in this paper were implemented in Python 3.7 programming language. The equipment used in this work has an Intel Core i5-5200u processor, CPU @2.20 GHz and 8.0 GB of random access memory installed.

Both WT and TCN have the unique advantage of being able to capture data characteristics in time series. Thus, this paper uses WT-TCN to construct a model to assess and forecast the pollution index of waterfowl ~~house~~houses. The implementation process for our model is shown in~~ ~~ Fig. 6. In this study, we first reduce or eliminate the noise of ~~environment~~environmental data by wavelet transform. Then, the ~~denoise~~denoised data and pollution index data were used as the input to train the TCN, and finally, ~~obtain~~ the waterfowl house environment quality assessment and prediction model was obtained.

Fig. 6~~Fig.6~~ Construction process of the PI prediction model ~~constructed process~~.

Results and discussion~~s~~

Simulation results and discussion~~s~~

In this study, we tested the TCN's memory and feature extraction capability for environment data sequences of different lengths. Fig. 7 shows that TCN consistently converges to ~~about~~approximately 0.01 MSE for all sequence lengths, whereas GRU and LSTM degenerate quickly as the sequence lengths grow. These results suggest that TCN is better at long sequence memory and feature extraction than its recurrent counterparts.

Fig. 7~~Fig.7~~ Performance of three models in sequences of different lengths.

Then, TCNs were used to predict PI at different ~~times interval~~time intervals, including

~~future~~ 12 hours, 24 hours, and 48 hours (predicting ~~future~~ 12 hours, 24 hours and 48 hours in the future every 20 minutes). Table~~.~~ 3 ~~shown~~shows that TCN has the best performance in all simulation results compared with the other models. We noted that ~~with~~as the prediction time interval ~~grew~~increased, the prediction effect ~~reduced~~decreased. This can be expected~~,~~ because a long prediction time interval needs a much longer effective history, which ~~was~~is a challenge for time series ~~model. Performance~~models. The performance of TCN fluctuates when the prediction time interval ~~changed form~~changes from 12 hours to 24 hours, but the wave motion ~~range~~ranges in MAE, RMSE and R_2 of TCN and GRU are less ~~the~~than 10%. However, the wave motion range in MAE, RMSE and R2 of LSTM ~~up to~~ reached 25% when the prediction time interval changed ~~form~~from 12 hours to 24 hours~~,~~ and ~~attain~~reached 112% when the prediction time interval changed ~~form~~from 24 hours to 48 hours. ~~Meanwhile~~Moreover, the traditional ~~model~~ SVM and RF ~~has bad~~models had poor performance in this study, ~~this~~which may be caused by the lack of long-~~-~~time series memory ability compared with the recurrent neural network. ~~Besides~~In addition, scatter plots, Taylor plots and box plots were also used here to visualize the predictive performance of various models.

Table 3~~Table.3~~ Comparison of model performance.

	MAE			RMSE			R^2		
Hours	12	24	48	12	24	48	12	48	48
TCN	0.0842	0.0859	0.1115	0.0154	0.0167	0.0273	0.9789	0.9791	0.9635
GRU	0.1728	0.1810	0.1922	0.0759	0.0789	0.0789	0.8937	0.8903	0.8898
LSTM	0.1892	0.2523	0.4434	0.0824	0.1388	0.2953	0.8896	0.8082	0.6078

SVM	0.5919	0.7245	0.8991	0.3292	0.3537	0.818 1	0.8512	0.774 8	0.661 7
RF	0.2744	0.5794	0.957 1	0.3407	0.3555	0.7513	0.880 9	0.805 0	0.679 9

The scatter plot in Fig. 8 visualized the agreement between the predicted and observed values of PI. In ~~the~~ Fig. 8, the baseline is drawn as a reference, and the perfect agreement between the observed and predicted data ~~was~~is described. SVM and RF models are far away from the best line. All three TCN models ~~shown~~showed outstanding prediction performance (points close to the best line). ~~Also~~Additionally, in the three phases, TCN ~~has~~is slightly far ~~away~~ from the best line when PI ~~up to~~reaches 3.5 ~~and~~or more, but GRU and LSTM perform worse. The reason for this phenomenon may be the input data of the environmental parameter C~~o~~O_2; ~~—~~all were in the normal range and no~~thing~~ data exceeded the standard value or were ~~distributes~~ distributed near the border value ~~(~~ (Fig. 5(d)) ~~)~~, leadings to the trained models having~~have~~ low sensitivity for a high PI and prediction difficulty~~ to predict~~.

(a) Future 12 ~~hours~~ hour prediction~~—~~. **(b) Future 24 ~~hours~~ hour prediction.**

(c) Future 48-hour~~s~~ prediction.

Fig. 8 Agreement between the observed and predicted PI values for the model considered in this study.

Furthermore, the models were also evaluated using Taylor plots for the three phases in Fig. 9. In a Taylor figure correlation coefficient, normalized standard deviation and RMSE were drawn, and the distance between the point corresponding to the model with the best prediction performance and the "observation" point ~~is~~was the least.

Formatted: Subscript

Again, Taylor plots showed that the TCN model has the best prediction performance.

(a) Future 12~~ hours ~~-hour prediction. **(b) Future 24~~ hours ~~-hour prediction.**

(c) Future 48~~-~~hour~~s~~ prediction.

Fig. 9 Normalized Taylor diagrams.

Finally, Fig. 10 compares ~~in box plot~~ the observed and predicted PI ~~values~~value dispersion and indicates the median (*M*) in a box plot. The performance of the three models was very similar~~,~~ in phase one, with the median values being very close (*$M_{observed}$=1.612, M_{TCN}=1.602, M_{GRU}=1.648, M_{LSTM}=1.719,M_{SVM}=1.569,* and *M_{RF}=1.854)*, but the box ~~'s~~ shape of TCN is closer to the box~~'s~~ shape of the observed data, ~~it~~ which also means that ~~the whole~~ all prediction results of TCN ~~was~~ were better than the others.~~—~~ In both phase two and phase three, the performances of LSTM and GRU ~~obvious gets worse as~~obviously worsen as the prediction interval grows, while the TCN performance ~~has~~does not change much. Overall, TCN substantially ~~outperform~~outperforms generic recurrent architectures such as LSTM and GRU.

(a) Future 12~~ hours ~~-hour prediction. **(b) Future ~~24hours~~24-hour prediction.**

(c) Future 48~~-~~hour~~s~~ prediction.

Fig. 10 Box plot of observed and predicted PI values.

In summary, TCN ~~that~~ combines best practices such as dilations and residual connections with the causal convolutions needed for autoregressive prediction. The experimental results indicate that for all prediction time interval phases, the TCN model provides high performance for PI forecasting, especially in the long prediction time interval problem. On the other hand, the LSTM model appears to be the 'weakest' model of all three models~~,~~; furthermore, it also

~~indicate~~indicates that a simple convolutional architecture is more effective across time sequence modeling tasks than recurrent architectures such as LSTM. On the other hand, a new type of temporal convoluted neural network is more competitive in the ~~waterfowl farm~~ PI time series prediction of waterfowl farms ~~compared with~~ than the traditional ~~Machine~~machine learning model.

Conclusions and future research

To further promote waterfowl house environment monitoring and controlling technology, reduce labor and increase the production effect, this study analyses the shortcomings of existing methods and introduces a new way to guide waterfowl house environment management by learning from other fields.

The new method investigates the application of denoised WT ~~denoised~~ and the performance of three neural network models and two mechanical learning models in predicting the pollution index of the waterfowl house environment using ~~environment~~environmental quality parameters at different intervals.~~Several wavelet function~~~~functions~~ ~~(Db4, Haar, Coif and Sym10) were considered in data denoising. Results~~~~The results~~ ~~suggest that wavelet function Db4 was suitable for ammonia data, Sym10 was suitable for temperature data, relative humidity data, and Co2~~ ~~CO_2~~ ~~data,~~ ~~and~~ ~~Coif was suitable for TSP data. Five denoised environment data~~~~environmental datasets~~ ~~(ammonia, temperature, relative humidity, CO_2, and TSP) were considered as input parameters. Results~~The results indicate that the TCN model has the best performance in predicting PI. The GRU model has similar performance~~,~~ but lower performance when the prediction time interval changed, and the LSTM model ~~was~~ performed the ~~least~~worst ~~performing~~ among the three models, although it still ~~provides~~ provided fairly accurate PI

predictions.

The models presented in this paper, ~~the TCN model,~~ in particular the TCN model, could provide ~~accuracy~~accurate and long-interval PI ~~prediction~~predictions of waterfowl house ~~environment~~environments and monitor ~~it~~them in real-~~-~~time. The simulation results show that this method can be applied in waterfowl house environment prediction. The future trend of the environment can be estimated and predicted compared with traditional real-time monitoring technology, which may allow better waterfowl house environment management practices, better culture plan design, and, in general, contribute to a more sustainable waterfowl house management approach.

The ~~environment~~environmental parameters selected in the present study may also pose a limitation because of the lack of equipment. Future work may include the use of more environmental parameters to evaluate PI and apply the model in more waterfowl breeding sites to improve the production effect and further verify the model.

Acknowledgment~~Acknowledgement~~s

The authors would like to thank native English experts Michele Genovese, Dr. Murtaza Hasan and AJE for revising the manuscript. Finally, ~~This~~this work was supported in part by the National Natural Science Foundation of China under Grants 61871475, 61471-131, and 61571444, in part by the special project of laboratory construction of Guangzhou Innovation Platform Construction Plan under Grant 201905010006, Guangzhou Innovation Pla~~n~~tform Construction Plan under Grant 2017B0101260016, ~~foundation~~ Foundation for High-level Talents in Higher Education of Guangdong Province under Grants 2017GCZX00014, 2016K-ZDXM0013, 2017KTSCX094, and 2018LM2168, and Beijing Natural Science Foundation

under Grant 4182023.

References

1. CSB CSB. Statistical Communique of the People's Republic of China on the 2019 National Economic and Social Development. 2020.
2. Qian Y, Song K, Hu T, Ying T. Environmental status of livestock and poultry sectors in China under current transformation stage. Sci Total Environ. 2018. doi:10.1016/j.scitotenv.2017.12.045
3. Valdebenito B ÁM, Pal S, Behrendt A, Wulfmeyer V, Lammel G. A novel approach for the characterisation of transport and optical properties of aerosol particles near sources - Part II: Microphysics-chemistry-transport model development and application. Atmos Environ. 2011. doi:10.1016/j.atmosenv.2010.09.004
4. Behrendt A, Pal S, Wulfmeyer V, Valdebenito B. ÁM, Lammel G. A novel approach for the characterization of transport and optical properties of aerosol particles near sources - Part I: Measurement of particle backscatter coefficient maps with a scanning UV lidar. Atmos Environ. 2011. doi:10.1016/j.atmosenv.2011.02.061
5. Pereira WF, Fonseca L da S, Putti FF, Góes BC, Naves L de P. Environmental monitoring in a poultry farm using an instrument developed with the internet of things concept. Comput Electron Agric. 2020. doi:10.1016/j.compag.2020.105257
6. Astill J, Dara RA, Fraser EDG, Roberts B, Sharif S. Smart poultry management: Smart sensors, big data, and the internet of things. Computers and Electronics in Agriculture. 2020. doi:10.1016/j.compag.2020.105291

7. Conley S. Poultry Barn Meteorology Managing air quality through humidity control. 2012. Available: https://www.canadianpoultrymag.com/%0Aproduction/bird-management/poultry-barn-meteorology-12641
8. Bustamante E, Guijarro E, García-Diego FJ, Balasch S, Hospitaler A, Torres AG. Multisensor system for isotemporal measurements to assess indoor climatic conditions in poultry farms. Sensors (Switzerland). 2012. doi:10.3390/s120505752
9. Lashari MH, Memon AA, Shah SAA, Nenwani K, Shafqat F. IoT Based poultry environment monitoring system. Proceedings - 2018 IEEE International Conference on Internet of Things and Intelligence System, IOTAIS 2018. 2019. doi:10.1109/IOTAIS.2018.8600837
10. Li D, Yang H. State-of-the-art Review for Internet of Things in Agriculture. Nongye Jixie Xuebao/Transactions of the Chinese Society for Agricultural Machinery. 2018. doi:10.6041/j.issn.1000-1298.2018.01.001
11. Liu S, Xu L, Jiang Y, Li D, Chen Y, Li Z. A hybrid WA-CPSO-LSSVR model for dissolved oxygen content prediction in crab culture. Eng Appl Artif Intell. 2014;29: 114–124. doi:10.1016/j.engappai.2013.09.019
12. Li C, Li Z, Wu J, Zhu L, Yue J. A hybrid model for dissolved oxygen prediction in aquaculture based on multi-scale features. Inf Process Agric. 2018. doi:10.1016/j.inpa.2017.11.002
13. Yang L, Liu C, Guo Y, Deng H, Li D, Duan Q. Prediction of Ammonia Concentration in Fattening Piggery Based on EMD-LSTM. Nongye Jixie Xuebao/Transactions Chinese Soc Agric Mach. 2019. doi:10.6041/j.issn.1000-1298.2019.S0.054

14. Liu CH, Yang L, Deng H, Guo YC, Li DL, Duan QL. Prediction of ammonia concentration in piggery based on ARIMA and BP neural network. Zhongguo Huanjing Kexue/China Environ Sci. 2019.

15. Jackman P, Ward S, Brennan L, Corkery G, McCarthy U. Application of wireless technologies to forward predict crop yields in the poultry production chain. Agric Eng Int CIGR J. 2015.

16. Asadollah SBHS, Sharafati A, Motta D, Yaseen ZM. River water quality index prediction and uncertainty analysis: A comparative study of machine learning models. J Environ Chem Eng. 2020. doi:10.1016/j.jece.2020.104599

17. Odiete WE. Novel metric for managing the protection of humanity and the environment against pollution and its adverse effects. Heliyon. 2020;6: e05555. doi:https://doi.org/10.1016/j.heliyon.2020.e05555

18. Martinus Y, Astono W, Hendrawan D. Water quality study of Sunter River in Jakarta, Indonesia. IOP Conference Series: Earth and Environmental Science. 2018. doi:10.1088/1755-1315/106/1/012022

19. Rubio-Arias H, Contreras-Caraveo M, Quintana RM, Saucedo-Teran RA, Pinales-Munguia A. An overall water quality index (WQI) for a man-made aquatic reservoir in Mexico. Int J Environ Res Public Health. 2012. doi:10.3390/ijerph9051687

20. Suriadikusumah A, Mulyani O, Sudirja R, Sofyan ET, Maulana MHR, Mulyono A. Analysis of the water quality at Cipeusing river, Indonesia using the pollution index method. Acta Ecol Sin. 2020. doi:10.1016/j.chnaes.2020.08.001

21. Leong WC, Kelani RO, Ahmad Z. Prediction of air pollution index (API) using

support vector machine (SVM). J Environ Chem Eng. 2020. doi:10.1016/j.jece.2019.103208

22. Thach TQ, Tsang H, Cao P, Ho LM. A novel method to construct an air quality index based on air pollution profiles. Int J Hyg Environ Health. 2018. doi:10.1016/j.ijheh.2017.09.012

23. Khanoranga, Khalid S. Phytomonitoring of air pollution around brick kilns in Balochistan province Pakistan through air pollution index and metal accumulation index. J Clean Prod. 2019. doi:10.1016/j.jclepro.2019.05.050

24. Yang H, Wang F, Yu J, Huang K, Zhang H, Fu Z. An improved weighted index for the assessment of heavy metal pollution in soils in Zhejiang, China. Environ Res. 2021. doi:10.1016/j.envres.2020.110246

25. Jorfi S, Maleki R, Jaafarzadeh N, Ahmadi M. Pollution load index for heavy metals in Mian-Ab plain soil, Khuzestan, Iran. Data Br. 2017. doi:10.1016/j.dib.2017.10.017

26. Ramsey DSL, O'Brien DJ, Smith RW, Cosgrove MK, Schmitt SM, Rudolph BA. Management of on-farm risk to livestock from bovine tuberculosis in Michigan, USA, white-tailed deer: Predictions from a spatially-explicit stochastic model. Prev Vet Med. 2016. doi:10.1016/j.prevetmed.2016.09.022

27. Choi HL, Sudiarto SIA, Renggaman A. Prediction of livestock manure and mixture higher heating value based on fundamental analysis. Fuel. 2014. doi:10.1016/j.fuel.2013.08.064

28. Kargar K, Samadianfard S, Parsa J, Nabipour N, Shamshirband S, Mosavi A, et al. Estimating longitudinal dispersion coefficient in natural streams using empirical

models and machine learning algorithms. Eng Appl Comput Fluid Mech. 2020. doi:10.1080/19942060.2020.1712260

29. Alizadeh MJ, Kavianpour MR, Danesh M, Adolf J, Shamshirband S, Chau KW. Effect of river flow on the quality of estuarine and coastal waters using machine learning models. Eng Appl Comput Fluid Mech. 2018. doi:10.1080/19942060.2018.1528480

30. Seng D, Zhang Q, Zhang X, Chen G, Chen X. Spatiotemporal prediction of air quality based on LSTM neural network. Alexandria Eng J. 2021;60: 2021–2032. doi:https://doi.org/10.1016/j.aej.2020.12.009

31. Csábrági A, Molnár S, Tanos P, Kovács J. Application of artificial neural networks to the forecasting of dissolved oxygen content in the Hungarian section of the river Danube. Ecol Eng. 2017;100: 63–72. doi:10.1016/j.ecoleng.2016.12.027

32. Ranković V, Radulović J, Radojević I, Ostojić A, Čomić L. Neural network modeling of dissolved oxygen in the Gruža reservoir, Serbia. Ecol Modell. 2010;221: 1239–1244. doi:10.1016/j.ecolmodel.2009.12.023

33. Sun M, Hassan SG, Li D. Models for estimating feed intake in aquaculture: A review. Comput Electron Agric. 2016;127: 425–438. doi:10.1016/j.compag.2016.06.024

34. Peng Q, Liu Z-W. Short-Term Residential Load Forecasting Based on Smart Meter Data Using Temporal Convolutional Networks. 2020 39th Chinese Control Conference (CCC). 2020. pp. 5423–5428. doi:10.23919/CCC50068.2020.9188453

35. Bai S, Kolter JZ, Koltun V. An empirical evaluation of generic convolutional and recurrent networks for sequence modeling. arXiv. 2018.

36. Bavkar S, Iyer B, Deosarkar S. Detection of Alcoholics using EEG and EMD Domain

Statistical Features. 2020 International Conference on Emerging Smart Computing and Informatics, ESCI 2020. 2020. pp. 153–157. doi:10.1109/ESCI48226.2020.9167564

37. Yan X, Boudrias MH, Mitsis GD. Artifact Removal in tACS-EEG Recordings: A Combined Methodology Based on the Empirical Wavelet Transform. Proceedings of the Annual International Conference of the IEEE Engineering in Medicine and Biology Society, EMBS. 2020. pp. 944–947. doi:10.1109/EMBC44109.2020.9176488

38. You SD, Hung MJ. Reducing Dimensionality of Spectro-Temporal Data by Independent Component Analysis. 2020 2nd International Conference on Computer Communication and the Internet, ICCCI 2020. 2020. pp. 93–97. doi:10.1109/ICCCI49374.2020.9145984

39. Kumar A, Tomar H, Mehla VK, Komaragiri R, Kumar M. Stationary wavelet transform based ECG signal denoising method. ISA Trans. 2020. doi:https://doi.org/10.1016/j.isatra.2020.12.029

40. Zhang L, Ling T, Yu B, Huang F, Zhang S. Intensive Interferences Processing for GPR signal based on the Wavelet Transform and F-K Filtering. J Appl Geophys. 2021. doi:10.1016/j.jappgeo.2021.104273

41. Saraiva SV, Carvalho F de O, Santos CAG, Barreto LC, Freire PK de MM. Daily streamflow forecasting in Sobradinho Reservoir using machine learning models coupled with wavelet transform and bootstrapping. Appl Soft Comput. 2021. doi:10.1016/j.asoc.2021.107081

42. David B, Moe RO, Michel V, Lund V, Mejdell C. Air Quality in Alternative Housing Systems May Have an Impact on Laying Hen Welfare. Part I—Dust. 2015; 495–511.

doi:10.3390/ani5030368

43. Najafi P, Zulkifli I, Amat Jajuli N, Farjam AS, Ramiah SK, Amir AA, et al. Environmental temperature and stocking density effects on acute phase proteins, heat shock protein 70, circulating corticosterone and performance in broiler chickens. Int J Biometeorol. 2015. doi:10.1007/s00484-015-0964-3

44. Gebregeziabhear E, Ameha N. The effect of stress on productivity of animals: a review. J Biol Agric Healthc. 2015;5: 165–172.

45. David B, Mejdell C, Michel V, Lund V, Moe RO. Air quality in alternative housing systems may have an impact on laying hen welfare. Part II-ammonia. Animals. 2015. doi:10.3390/ani5030389

46. K.Othman J, R. Mahmood J, Y. A. Al- Emarah G. Design and Implementation of Smart Relay based Remote Monitoring and Controlling of Ammonia in Poultry Houses. Int J Comput Appl. 2014. doi:10.5120/18093-9149

47. Mallat SG. A Theory for Multiresolution Signal Decomposition: The Wavelet Representation. IEEE Trans Pattern Anal Mach Intell. 1989. doi:10.1109/34.192463

48. Breiman L. Random forests. Mach Learn. 2001. doi:10.1023/A:1010933404324

49. Sepp H, J?urgen S. 1997Hochreiter_LSTM. Neural Comput. 1997.

50. Zhang Z, Chen H, Huang Y, Lee WJ. Quantile Huber Function Guided TCN for Short-Term Consumer-Side Probabilistic Load Forecasting. 2020 IEEE/IAS Industrial and Commercial Power System Asia, I and CPS Asia 2020. 2020. doi:10.1109/ICPSAsia48933.2020.9208545

51. Long J, Shelhamer E, Darrell T. Fully convolutional networks for semantic

segmentation. Proceedings of the IEEE Computer Society Conference on Computer Vision and Pattern Recognition. 2015. doi:10.1109/CVPR.2015.7298965

52. He K, Zhang X, Ren S, Sun J. Deep residual learning for image recognition. Proceedings of the IEEE Computer Society Conference on Computer Vision and Pattern Recognition. 2016. doi:10.1109/CVPR.2016.90

Response to Journal:

Reviewer #1: While I commend the authors for working diligently on the suggested comments by both reviewers, the manuscript is still not in a publishable state. I still maintain that the work is interesting and prudent, but the authors have not fully addressed some of the major comments brought up previously. First being the English is still not at a level of an academic paper and really needs major edits which I cannot put forth the time to do.

Answer: Thank you very much for your prestigious comments. This paper was edited for proper English language, grammar, punctuation, spelling, and overall style by one or more of the highly qualified native English speaking editors at AJE.

AJE

Editing Certificate

This document certifies that the manuscript

Pollution index of waterfowl farm assessment and prediction based on temporal convoluted network

prepared by the authors

Jiande Huang, Shuangyin Liu, Shahbaz Gul Hassan,Longqin Xu

was edited for proper English language, grammar, punctuation, spelling, and overall style by one or more of the highly qualified native English speaking editors at AJE.

This certificate was issued on **May 4, 2021** and may be verified on the AJE website using the verification code **09C4-B4D5-4FF4-9D83-C383** .

Neither the research content nor the authors' intentions were altered in any way during the editing process. Documents receiving this certification should be English-ready for publication; however, the author has the ability to accept or reject our suggestions and changes. To verify the final AJE edited version, please visit our verification page at aje.com/certificate. If you have any questions or concerns about this edited document, please contact AJE at support@aje.com.

AJE provides a range of editing, translation, and manuscript services for researchers and publishers around the world. For more information about our company, services, and partner discounts, please visit aje.com.

Second, the paper is still very methods focus despite the fact that both reviewers pointed out that there needs to be more on the "why" do the study. What's the motivation? How can it help farmers. The authors added a few lines here and there but it is still not compelling.

Answer: We have added more references to this study. It includes why we need to monitor and control the poultry house environment, what methods have been used to do that thing by others, how can they help the farmer and why we need the new method.

Labor shortages and increasing biosecurity practices will make it more difficult for producers to monitor and manage the production, health, and welfare status of all of their birds.

Employing modern poultry management technology is necessary to increase production [6]. An example of how modern management technology can be used to monitor and control the poultry house environment is exemplified by humidity regulation via ventilation rate changes mediated by relative humidity sensors, as relative humidity is one of the more important environmental aspects of a poultry house [7]. In addition, more advanced systems are being researched. Bustamante et al. used a multisensor system to effectively assess barn ventilation system function by tracking temperature, air velocity and differential pressure in broiler houses [8]. Hanif et al. proposed an internet of things technology-based protection and monitoring of the environment of a poultry house to monitor the environment-related parameters such as air temperature, air humidity, CO_2 level of concentration and ammonia concentration., which has been implemented successfully, leading to a safe environment and profit for the poultry industry [9]. The techniques mentioned above are both solutions for real-time environmental monitoring and control; however, relying on hardware monitoring in real time cannot capture the trend of environmental changes [10], and it is easy to miss the best time for adjustment, which leads to waterfowl health damage and property loss.

Cultivation environment forecasting has been studied for many years and has made some achievements in aquaculture and livestock breeding [11–14]. The technique estimates or predicts the future changes in target variables that cannot be obtained directly. For example, Jackman et al. generated a prediction model by using sensor inputs of relative humidity, carbon dioxide, temperature, and ammonia for environmental parameter and crop yield prediction [15]. In waterfowl production, a system such as this would allow for actions to be taken sooner by farmers if the environment is projected to be bad. However, few studies have applied prediction

in waterfowl breeding. Therefore, it is necessary to apply environmental prediction technology to waterfowl production to fill this gap.

1. While the first table highlights the data chosen for the PI. The text does not go into much detail about why these were chosen, and what are the acceptable limits. I still do not see a mention of other similar studies on waterfowl or poultry in the introduction.

Answer: We have added more references to detail describe why data were chosen, and what are the acceptable limits.

Labor shortages and increasing biosecurity practices will make it more difficult for producers to monitor and manage the production, health, and welfare status of all of their birds. Employing modern poultry management technology is necessary to increase production [6]. An example of how modern management technology can be used to monitor and control the poultry house environment is exemplified by humidity regulation via ventilation rate changes mediated by relative humidity sensors, as relative humidity is one of the more important environmental aspects of a poultry house [7]. In addition, more advanced systems are being researched. Bustamante et al. used a multisensor system to effectively assess barn ventilation system function by tracking temperature, air velocity and differential pressure in broiler houses [8]. Hanif et al. proposed an internet of things technology-based protection and monitoring of the environment of a poultry house to monitor the environment-related parameters such as air temperature, air humidity, CO_2 level of concentration and ammonia concentration., which has been implemented successfully, leading to a safe environment and profit for the poultry industry [9]. The techniques mentioned above are both solutions for real-time environmental monitoring and control; however, relying on hardware monitoring in real time cannot capture the trend of environmental changes [10], and it is easy to miss the best time for adjustment, which leads to

waterfowl health damage and property loss.

Cultivation environment forecasting has been studied for many years and has made some achievements in aquaculture and livestock breeding [11–14]. The technique estimates or predicts the future changes in target variables that cannot be obtained directly. For example, Jackman et al. generated a prediction model by using sensor inputs of relative humidity, carbon dioxide, temperature, and ammonia for environmental parameter and crop yield prediction [15]. In waterfowl production, a system such as this would allow for actions to be taken sooner by farmers if the environment is projected to be bad. However, few studies have applied prediction in waterfowl breeding. Therefore, it is necessary to apply environmental prediction technology to waterfowl production to fill this gap.

According to the importance of the environment and expert research, we selected 5 environmental factors, as shown in Table 1. Among them, ammonia is a toxic gas and the greatest concern of environmental pollution in waterfowl production, adversely affecting the ecosystem, environment, and health of birds and people. Less than 10 ppm is the ideal limit [42]. Relative humidity can impact bird health, and high relative humidity may worsen broiler geese performance; the ideal value is 70% [43]. Heat stress is a major concern in waterfowl production; high and low temperatures will reduce the growth performance and survivability of waterfowl, and temperatures less than 27°C and more than 10°C are ideal [44]. TSP is from the birds themselves as well as from the feed, litter, and building materials and may serve as a pathogen disseminator and bring about lung damage; less than 10 mg/m^3 is the ideal limit [42]. Additionally, the levels of relative humidity, ammonia, carbon dioxide, temperature and TSP are known to be correlated with each other [6]. These factors are important for waterfowl

production and can be monitored and optimized.

2. There is now way too much detail in the methods. I'm not sure you need every detail and plot for each AI technique in your paper unless this is the first time they are introduced. You should provide a brief summary of each and then if you want to include this detail move to SI.

Answer: We have simplify introduction of some methods.

Wavelet transform

Continuous wavelet transforms for a given waterfowl environment signal *s(k)* can be described as follows:

$$WTs(a,b) = \frac{1}{\sqrt{a}} \int_{\infty}^{-\infty} s(k) \overline{\left(\psi\left(\frac{t-b}{a} \right) \right)} dx \tag{3}$$

where $\overline{(\ )}$ denotes the complex conjugate, $\psi(x)$ is the wavelet function, *a* is the time scale dilation and *b* is the time translation. By controlling the values of parameters *a* and *b*, signal time-frequency positioning can be achieved. The wavelet transform can decompose signals into multiple resolutions. However, the symbols in realization communication are discrete data, so the continuous wavelet transform needs to be discretized. Suppose $a = a_0^j, b = kb_0 a_0^j, j,k \in Z$, when $a_0 = 2, b_0 = 1$:

$$\psi_{j,k}(n) = 2^{-j/2} \psi(2^{-j} n - k) \tag{4}$$

The discrete waterfowl environment signal s(n) can be transformed as:

$$D(j,k) = \sum_k s(n) \overline{\psi_{j,k}}(n) \tag{5}$$

The Mallat algorithm can quickly calculate the orthogonal wavelet transform coefficients and realize signal decomposition and reconstruction [47]. The approximation coefficients $s_{j+1}(n)$ and detail coefficients $d_{j+1}(n)$ can be recurrently related by the Mallat algorithm as follows:

$$s_{j+1}(k) = \sum_n h_0(n-2k)s_j(n) \quad (6)$$

$$d_{j+1}(k) = \sum_n h_1(n-2k)s_j(n) \quad (7)$$

where h_0 and h_1 are the high-pass filter and low-pass filter, respectively. The reconstruction is the inverse process of decomposition:

$$s_j(k) = \sum_n s_{j+1}(n)h_0(k-2n) + \sum_n d_{j+1}(n)h_1(k-2n) \quad (8)$$

The wavelet transform can decompose noise signals into different signal channels at various levels of resolution. In general, the useful signals are distributed in the high-level resolution channel, and the noise signal is likely to be distributed in the lower-level channel. Therefore, the noise of the original data can be reduced as much as possible based on a reasonable threshold. Therefore, the wavelet denoised algorithm can be described as follows:

(1) Perform wavelet transform on the original signal *s(k)* to obtain wavelet coefficients of various scales.

(2) Determine the threshold and perform threshold denoising on different wavelet coefficients to obtain estimated wavelet coefficients.

(3) Conduct wavelet reconstruction by equation 11 to obtain the denoise signal $\widehat{s(k)}$.

Support vector machine

SVM has good generalization ability in solving nonlinear, small sample, and high-dimensional pattern recognition, and the optimal solution obtained is global, which solves the local optimal problem that cannot be avoided in other algorithms. The prediction process of the SVM includes support vector determination, kernel function selection, kernel parameter determination, and solution. The mathematical expression as follow:

$$N_t = \sum_{SVS} \alpha_i y_i K(x_i, x) + b_0 \quad (9)$$

~~Where *SVS* is the number of support vectors, α_i is the Lagrangian coefficient of each training sample, y_i (-1 or 1) is the vector label, $K(x_i, x)$ is the selected kernel function, and b_0 is the bias. The kernel function replaces the linear quantity in the traditional linear equation and maps the data to high-dimensional space processing. After selected the kernel function, the samples are trained to establish the SVM model and the prediction result *Nt* is finally obtained.~~

Long short-term memory neural network

The LSTM neural network is a special kind of recurrent neural network. It was first proposed by Ho-chreiter and Schmidhuber [49]. Its appearance effectively solved the gradient explosion problem of traditional recurrent neural networks. At the same time, the LSTM neural network has long-term memory to handle long-term sequence data.~~ The LSTM cell structure consists of forget gate, input gate, output gate and cell state. LSTM structure is shown in Fig 2 At the time *t*, there are three input parameters of the LSTM network: input value x_t, at time *t*, output value h_{t-1} and cell state C_{t-1} at *t-1*. There are two output parameters of the LSTM network: output value h_t and cell state C_t at time *t*. Through the activation function σ LSTM realizes the control of the three gates so as to realize the retention and forgetting of historical information.~~

~~**Fig.2. The structure of LSTM**~~

Gated recurrent unit

A gated recurrent unit (GRU) is a type of recurrent neural network (RNN). Similar to a long short-term memory neural network (LSTM), it is also proposed to solve long-term and gradient backpropagation problems. LSTM and GRU have similar performances, but compared with LSTM,

GRU is computationally cheaper. The structure of GRU is shown in Fig 3 GRU can be descried as follows:

Firstly, two output gateds, *r* (reset gated) and *z* (update gated), were obtained by the input *X(t)* at *t* time and the output *h(t − 1)* at *t − 1* time.

$$r = \sigma(W_r \cdot [h(t-1), X(t)]) \quad (10)$$

$$z = \sigma(W_z \cdot [h(t-1), X(t)]) \quad (11)$$

Where σ CJ is a sigmoid function. Parameter *h'* selective re- members *X(t)*. Trough the reset gated *r* we can obtain *h'* as follows:

$$h' = \tanh(W_h \cdot [r * h(t-1), X(t)]) \quad (12)$$

Finally, the output *y(t)* and hidden state *h(t)* at t time can be calculated as follows:

$$h(t) = (1-z) * h(t-1) + z * h' \quad (13)$$

$$y(t) = \sigma(W' \cdot h(t)) \quad (14)$$

Where [ ] denoted the connection of two vectors and * denoted the product of matrices.

Fig.3. The structure of GRU

3. Conclusions and future research again focus way too much on methods and not on the "why".

Answer: We have modified the conclusions.

To further promote waterfowl house environment monitoring and controlling technology, reduce labor and increase the production effect, this study analyses the shortcomings of existing methods and introduces a new way to guide waterfowl house environment management by learning from other fields.

The new method investigates the application of denoised WT and the performance of three neural network models and two mechanical learning models in predicting the pollution index

of the waterfowl house environment using environmental quality parameters at different intervals. The results indicate that the TCN model has the best performance in predicting PI. The GRU model has similar performance but lower performance when the prediction time interval changed, and the LSTM model performed the worst among the three models, although it still provided fairly accurate PI predictions.

The models presented in this paper, in particular the TCN model, could provide accurate and long-interval PI predictions of waterfowl house environments and monitor them in real time. The simulation results show that this method can be applied in waterfowl house environment prediction. The future trend of the environment can be estimated and predicted compared with traditional real-time monitoring technology, which can allow better waterfowl house environment management practices, better culture plan design, and, in general, contribute to a more sustainable waterfowl house management approach.

The environmental parameters selected in the present study may also pose a limitation because of the lack of equipment. Future work may include the use of more environmental parameters to evaluate PI and apply the model in more waterfowl breeding sites to improve the production effect and further verify the model.
