# Supplementary material for: Pollution index of waterfowl farm assessment and prediction based on temporal convoluted network
Source: PLoS One. 2021 Jul 23;16(7):e0254179. doi: 10.1371/journal.pone.0254179 (PMC8301615; doi:10.1371/journal.pone.0254179)
Supplement: Attachment — Submitted filename: Response to Reviewers.docx [file pone.0254179.s002.docx]

Response to Journal:

1. Please ensure that your manuscript meets PLOS ONE's style requirements, including those for file naming. The PLOS ONE style templates can be found at

https://journals.plos.org/plosone/s/file?id=wjVg/PLOSOne_formatting_sample_main_body.pdf and

<https://journals.plos.org/plosone/s/file?id=ba62/PLOSOne_formatting_sample_title_authors_affiliations.pdf>

Answer: We have modified the format following your suggestion. Please see the highlighted version of the manuscripts.

2. PLOS requires an ORCID iD for the corresponding author in Editorial Manager on papers submitted after December 6th, 2016. Please ensure that you have an ORCID iD and that it is validated in Editorial Manager. To do this, go to ‘Update my Information’ (in the upper left-hand corner of the main menu), and click on the Fetch/Validate link next to the ORCID field. This will take you to the ORCID site and allow you to create a new iD or authenticate a pre-existing iD in Editorial Manager. Please see the following video for instructions on linking an ORCID iD to your Editorial Manager account: <https://www.youtube.com/watch?v=_xcclfuvtxQ>

Answer:Following your suggesstions we have updated ORCID.

3. We note you have included a table to which you do not refer in the text of your manuscript. Please ensure that you refer to Table 3 in your text; if accepted, production will need this reference to link the reader to the Table.

Answer: We have modified the changes following your suggesstion, which are:

Table.3 shown that TCN has the best performance in all simulation results compared with other

Reviewer #1: This manuscript develops a method to apply AI to predict a pollution index for a poultry farm in China. While I think the methods have promise, the authors need to address the following major issues before it is suitable for publication.

Major:

> The English and grammar are fair to poor throughout. I have included in the minor comments below some instances in just the abstract and first couple of paragraphs but I do not have time to edit the entire manuscript. This needs to be addressed as it made it difficult to follow along.

Answer: Thank you very much for your prestigious comments we have modified the manuscript and made changes.

Comment: The motivation for the paper is lacking. Why do we need such a robust prediction method? Can the facilities actually use it to make changes? If so, does the interval required to make adjustments align with the model's ability to predict (Figure 8). For example, L58-59 is the only place you mention that it would benefit farmers, but would it? In what way? How could it be used?

Answer: Provide a good growing environment for the goose to ensure the normal growth and development of the goose. For example, by predicting future changes in PI the farmer can make arrangements for the work time or power of environmental control equipment such as fans, exhaust fans, and/or nebulizers in advance.

Comment: At its most basic premise, this paper is quite simple. Calculate a very simple pollution index from a few variables and see if you can predict with ML methods. It would benefit by comparing these computationally intensive methods to simpler prediction methods...does it even warrant the use

of ML? What about other pollution indices? Or individual variables? Another example is you state that (L56-58) traditional methods have bad performance. So why not show that with your data to really make the case for your work?

Answer: Following your suggestion we make comparison with tradition method and the results are given below:

**Table.3 Comparison of model performance.**

|  | MAE | | | RMSE | | | R^2^ | | |
| --- | --- | --- | --- | --- | --- | --- | --- | --- | --- |
| Hours | 12 | 24 | 48 | 12 | 24 | 48 | 12 | 48 | 48 |
| TCN | 0.0842 | 0.0859 | 0.1115 | 0.0154 | 0.0167 | 0.0273 | 0.9789 | 0.9791 | 0.9635 |
| GRU | 0.1728 | 0.1810 | 0.1922 | 0.0759 | 0.0789 | 0.0789 | 0.8937 | 0.8903 | 0.8898 |
| LSTM | 0.1892 | 0.2523 | 0.4434 | 0.0824 | 0.1388 | 0.2953 | 0.8896 | 0.8082 | 0.6078 |
| SVM | 0.5919 | 0.7245 | 0.8991 | 0.3292 | 0.3537 | 0.8181 | 0.8512 | 0.7748 | 0.6617 |
| RF | 0.2744 | 0.5794 | 0.9571 | 0.3407 | 0.3555 | 0.7513 | 0.8809 | 0.8050 | 0.6799 |

Comment: L73-74: You state that TCN can better solve the modeling problem associated with PI, but you have yet to establish what those problems are. Is it just the noise which you discuss in the next paragraph? If so, why not compare the traditional methods with denoised data as well to see if the denoising was the most helpful thing or the ML approach?

Answer: PI prediction is a long sequence time series modeling problem, but the traditional model has low precision in long sequence time series modeling. So, we use TCN to solve the long sequence time series modeling problem.

TCN has been proved that performs well in long sequence time series modeling. However, TCN has not yet been applied in PI prediction. Therefore, a better TCN prediction architecture is explored to promote the predicted precision of PI in this study.

Comment: L92-95: no need to outline the paper. Delete these lines.

Answer: Following your suggestion we have deleted these lines.

Comment: Section 2.1 --you need a LOT more information on the study area. Include a map of where Shanwei is. L 99-101 describe the system but a schematic would be helpful including where the sampling is occurring. I'm also confused about what kind of animal is in the farm. A couple of times the paper mentions chicken but here it says waterfowl. So, what kind of animal is in the farm and how many are there typically? What kind of structure is the facility? Are their air ventilation systems, etc. that can help to control the parameters being measured? The data source section is missing a lot of information. I cannot tell what most of the things in Figure 1 are. It needs a narrative or more details. What kinds of sensors were there? What methods were they using to detect? What is the time frame of collection? What season did you collect data?

Answer: Following your suggestion we have improve data source information as given below, and the animal in the farm is geese we have made changes throughout manuscript.

 **Fig.1 Schematic diagram of the waterfowl farm monitoring based on the Internet of Things.**

Comment: Section 2.4 you need to include descriptions of all of the methods you used, not just TCN (eg LSTM, GRU)

Answer: Following your suggestion we have included the description of all method given below:

**Support vector machine**

SVM has good generalization ability in solving non-linear, small sample, high-dimensional pattern recognition, and the optimal solution obtained is global, which solves the local optimal problem that cannot be avoided in other algorithms. The prediction process of the SVM includes the support vector determination, kernel function selection, kernel parameter determination, and solution. The mathematical expression as follow:

 (9)

Where *SVS* is the number of support vectors, *α_i_* is the Lagrangian coefficient of each training sample, *y_i_* (-1 or 1) is the vector label, *K (x_i_, x)* is the selected kernel function, and *b_0_* is the bias. The kernel function replaces the linear quantity in the traditional linear equation and maps the data to high-dimensional space processing. After selected the kernel function, the samples are trained to establish the SVM model and the prediction result *Nt* is finally obtained.

**Random forest**

RF is a non-linear ensemble model that establishes and averages a large number of random distribution decision tree for regression or classification tasks [38]. A decision tree or classification and regression tree that construct the RF is a non-parametric model. According to the complexity of the input data, the tree grows in the process of the learning. Decision nodes and leaf nodes are the main components of decision tree. Each input sample is estimated by a test function of decision nodes and passed to different branches according to the features of the sample. After all trees are trained, each tree can predict the test sample set according to the node threshold, and the results of each tree are combined to vote to determine the final result of the entire random forest.

**Long short-term memory neural network.**

LSTM neural network is a special kind of recurrent neural network. It was first proposed by Ho-chreiter and Schmidhuber [39]. Its appearance effectively solved the gradient explosion problem of traditional recurrent neural network. At the same time, LSTM neural network has long-term memory to handle long-term sequence data.

The LSTM cell structure consists of forget gate, input gate, output gate and cell state. LSTM structure is shown in Figure. At the time *t*, there are three input parameters of the LSTM network: input value *x_t,_* at time *t*, output value *h_t-1_* and cell state *C_t-1_* at *t-1*. There are two output parameters of the LSTM network: output value *h_t_* and cell state *C_t_* at time *t.* Through the activation function *σ* LSTM realizes the control of the three gates so as to realize the retention and forgetting of historical information.

**Fig.2. The structure of LSTM**

**Gated recurrent unit.**

GRU is one type of Recurrent Neural Network. Like the Long-short term memory Neural Network (LSTM), it is also proposed to solve long-term and gradient backpropagation problems. The structure of GRU is shown in Fig.3. GRU can be descried as follows:

Firstly, two output gateds, *r* (reset gated) and *z* (update gated), were obtained by the input *X(t)* at *t* time and the output *h(t − 1)* at *t − 1* time.

 (10)

 (11)

Where CJ is a sigmoid function. Parameter *h'* selective re- members *X(t)*. Trough the reset gated *r* we can obtain *h’* as follows:

 (12)

Finally, the output *y(t)* and hidden state *h(t)* at t time can be calculated as follows:

 (13)

 (14)

Where [ ] denoted the connection of two vectors and * denoted the product of matrices.

**Fig.3. The structure of GRU**

> You have SO MANY methods in the results. All of sections 4.1, 4.2, 4.3, then lines 251-255 of section 4.4 are methods and need to be in the methods section.

Answer: Following your suggestion we have modified the changes:

**Processing of environment data**

Different environment factors may contain different kind of noise, to achieve the best effect of denoise process, obtain quality data, we adopt four wavelet function include Db4, Haar, Coif, and Sym10 to process each environment data, respectively. Signal-noise ratio (SNR) and normalized cross correlation (NCC) were used to evaluate the denoised effect which can be described as:

 (17)

 (18)

Where *I_1_* is denoised signal and *I_2_* is original signal, the larger the SNR and NCC, the better the noise reduction effect, theoretically. As shown as the Table.2, wavelet function Db4 was suitable for ammonia data (SNR=9.4318, NCC=0.9485), Sym10 was suitable for temperature data (SNR=7.4635, NCC=0.9221), relative humidity data (SNR=4.0809, NCC=0.8630), and Co_2_ data (SNR=1.7785, NCC=0.7793), Coif was suitable for TSP data (SNR=13.1443, NCC=0.9771). The best denoised results of each environment data was showed as Fig.6.

Table.2 Denoised performance of five parameters.

| Target | Performance criteria | Wavelet function | | | |
| --- | --- | --- | --- | --- | --- |
|  |  | Db4 | Haar | Coif | Sym10 |
| Ammonia | SNR/(dB) | 9.4318 | 8.8287 | 9.3769 | 9.3602 |
|  | NCC | 0.9485 | 0.9432 | 0.9484 | 0.9474 |
| Temperature | SNR/(dB) | 7.2491 | 6.5372 | 7.0205 | 7.4635 |
|  | NCC | 0.9192 | 0.9061 | 0.9149 | 0.9221 |
| Relative humidity | SNR/(dB) | 3.9422 | 3.6395 | 3.8920 | 4.0809 |
|  | NCC | 0.8600 | 0.8559 | 0.8596 | 0.8630 |
| Co_2_ | SNR/(dB) | 1.7064 | 1.3855 | 1.4991 | 1.7785 |
|  | NCC | 0.7764 | 0.7642 | 0.7689 | 0.7793 |
| TSP | SNR/(dB) | 12.8848 | 12.6578 | 13.1443 | 13.1142 |
|  | NCC | 0.9756 | 0.9745 | 0.9771 | 0.9769 |

(a) **Ammonia sequence.**

**(b) Temperature sequence.**

**(c) Relative humidity sequence.**

**(d) Co2 sequence.**

**(e) TSP sequence.**

**Fig.8 Results of noise reduction of five parameters by WT.**

**Performance criteria**

In this paper, mean absolute error (MAE), root mean square error (RMSE) and coefficient of determination (R^2^) are selected to measure the prediction accuracy and operation efficiency, and MAE, RMSE and R^2^ are defined as follows:

 (19)

 (20)

 (21)

Where *N* is the total number of actual points in the data *yi* is the observed value of period *i*, *y_i_* is the prediction value of period *i*, *y* is the average of observed values, *y_t_* is the average of prediction values.

Comment: It's not clear from your methods how you split up to test the prediction of future time intervals. What time intervals were used for training? For example were your training sets 12 hour intervals trying to predict future 12, 24, and 48? It's unclear

Answer: Our training sets 20 minute intervals trying to prediction future 12, 24, and 48 hours.

(L335-336)Then, TCNs were used to predict PI at different times interval, including future 12 hours, 24 hours, and 48 hours (predicting future 12 hours, 24 hours and 48 hours every 20 minutes).

Comment: There is a severe lack of discussion here. What does it mean that you can predict reasonably well the future 12 hours? How does the fact that your data come from a very short time period in one year affect your results? Why do all of your predictions fail in the higher levels of pollution indices? It would be helpful to provide plots (even if in SI) of the individual data that went into the PI predictions. When the PI was high and difficult to predict, which variables were responsible, and how could this help improve future predictions? There is a lot of information hiding behind the PI calculation that would be useful to readers and for you to dive into.

Answer: The data we collected in July 1^th^ to September 28^th^, 2019. The data for this time period is representative, because this time period is important for geese to lay eggs off-season. Only precise control of the environment can ensure the normal egg production of the goose.

The reason for this phenomenon may be the input data of environmental parameter Co2 all in the normal range and nothing data exceed the standard value or distributes near the border value（fig 8(d)）, leads to the trained models have low sensitivity for high PI and difficult to predict.

Collecting more abnormal CO_2_ data or use a weighting method to reduce the weight of CO_2_ may help improve future predictions.

Comment: Your last line 329-330 leaves me wondering what international guidelines you are referring to and would your study have been better if you used them, so why didn't you use them and what are they?

Answer: Due to confusing statement we have removed these line.

Figures:

Comment: - very confusing and not enough detail. Again, you need to describe the equipment used to obtain the data and the methods on board.

Answer: we have modified the figure, and provided the suggested information as follows:

Comment: I don't think this is quite right. Shouldn't environmental data that has been wavelet transformed have an arrow going into the pollution index, and not into the TCN? And didn't you calculate GRU and LTSM as well so where are they?

Answer: we have modified changes following your suggesstion as shown in figure below:

Comment: Figure 6 - units for data? what kind of range do these data have? What is the x-axis?

Minor (only for abstract and first paragraph):

Answer: Following your suggesstion we have made changes as follows:

Comment: L 20-21: The first sentence is very strong, do you have anything to back up this claim? If not, reword.

Answer: we have made changes following your suggesstion, which are:

Environment quality is a major factor that directly impacts waterfowl productivity.

Comment: L21-22: Sounds like you invented TCN and WT, which I don't think is the case so reword. I think what you mean is your study uses/applied these methods.

Answer: we have made changes following your suggesstion, which are:

This study applied a new neural network model called temporal convolutional network (TCN) and a denoising algorithm called wavelet transform (WT) for predictin..

Comment: L23: the poultry to a poultry

Answer: we have made changes following your suggesstion, which are:

Comment: L23: Shanwei may not mean anything to people outside of China so suggest saying China or in Shanwei, China

Answer: we have made changes following your suggesstion, which are:

PI values at a waterfowl farm in Shanwei, China.

Comment: L24: is to was

Answer: we have made changes following your suggesstion, which are:

The TCN model performance was compared

Comment: L26: Probably better to use "environmental" than "environment" when referring to the data you used throughout.

Answer: we have made changes following your suggesstion, which are:

Denoised environmental data including….

Comment: L27: Co2 should be CO2 (with the 2 as a subscript)

Answer: we have made changes following your suggesstion, which are:

Comment: L27: I've never heard it called "total particulate suspended". Do you mean total suspended particles?

Answer: we have made changes following your suggesstion, which are:

and total suspended particles (TSP)

Comment: L27: con-structing is just constructing

Answer: we have made changes following your suggesstion.

Comment: L27: is to are

Answer: we have made changes following your suggesstion.

Comment: L28: "the" in front of TCN

Answer: we have made changes following your suggesstion.

Comment: L29: prediction

Answer: we have made changes following your suggesstion such as.

TCN model in general produces a more precise PI prediction and provides the highest ..

Comment: L29: phases

Answer: we have made changes following your suggesstion such as:

prediction accuracy for all phases (MAE = 0.0842, 0.0859, 0.1115, RMSE = 0.0154, 0.0167,…

Comment: L32: generalization to general

Answer: we have made changes following your suggesstion such as:

general performance compared with other parallel forecasting models and is a suitable and

Comment: L32: per-formance is just performance

Answer: we have made changes following your suggesstion such as

general performance compared with other parallel forecasting models and is a suitable and…

Comment: In general, do not use acronyms in your abstract if you never use them later (eg. CO2, TPS, GRU, LTSM)

Answer: we have made changes following your suggesstion, acronyms are removed

Comment: L37-38: This first sentence is just odd. Suggest rewording to something like, "In response to growing global demand for food (or do you mean specifically poultry?), the Chinese poultry industry has grown to be a leader in meat and egg production".

Answer: we have made changes following your suggesstion such as:

In response to the growing global demand for food, the Chinese waterfowl industry has grown to be a leader in both goose meat and goose egg production [1][2].

Comment: L38-39: The first clause adds nothing to the sentence and so your sentence doesn't make sense. Why is there such a concern that poultry need to be grown in a controlled environment? You have not made that clear.

Answer:However, as the scale of intensive culture increases, there is a growing concern, in China, that waterfowl should be raised under conditions that promote animal welfare since productivity is related to environmental conditions in which the waterfowl are raised[3].

Comment: L39-41: "In this sense" should be "because of this desire/need/concern" if you correct the sentence before. Then you have a sort of run-on here. I think what you are trying to say is that poultry farms need to be controlled environments that can maintain a healthy ecosystem and farm productivity? or something like that.

Answer: we have made changes following your suggesstion such as:

According to this, to maintain and control optimal conditions for survival and good goose growth, establishing a habitat to be closer to a standard ecosystem is justified[4].

Comment: L41-44: reword to "Thus the main goal of controlled environments for poultry production are to maintain optimal conditions for survival and growth by establishing a habitat similar to their typical natural environment." And with this sentence as such you may not need the sentence before as it would be redundant.

Answer : we have modified and delete the redundant sentence.

Reviewer #2:

I have provided below some suggestions. But, the poor quality of the language did not help me to make a comprehensive review. I highly encourage the authors to reach out to some colleagues for help and get some editing done before re-submission. The topic is interesting, but, the manuscript contains mainly some generic information about the WT which are well known. Also, there should be more emphasis on the need for this study on global scale. please make some review work on this topic for other countries of the world.

Answer: thank you very much for your prestigious comments we have made changes following your suggestions given below:

In recent years, many studies on pollution models have been carried out to evaluate the quality of environment base on PI method, such as river's quality status assessment and prediction[8,10–12], air’s quality status assessment and prediction [13–15], and soil’s pollution assessment and prediction [16,17]. Forecasting the concentration of water, air, and soil pollutants is an effective method for protecting public health, productivity, and travel by providing early warnings of harmful pollutants. Likewise, it is also important to assess and predict PI in advance for waterfowl farms so that, if necessary, the producer can intervene more quickly by using management practices in order to ensure a health context.

Comment: Line 201: It is not clear, what is the projection time for these analyses! Authors mentioned “forecast the pollution index of waterfowl house”. Pleas provide an appropriate time scale.

Answer: we have made changes following your suggesstion given below:

In this report, environment data includes ammonia, relative humidity, temperature, Co_2_ and TSP were collected from July 1^th^ to September 28^th^, 2019, at intervals of 20 minutes. 72 sets of data collected per day yield a total of 6480 observations samples. For a model generation, the first 4536 sets of the data were used for model training, and the remaining 1944 sets were used as the testing data to estimate the prediction performance of the constructed model.

Comment: On page 14, authors mentioned “Perform wavelet transform on the original signal s(k) to obtain wavelet coefficients of various scales.” These are very basic or generic statement about the WT. Please provide most useful information only related to this study. Authors are also encouraged to see other applications of WT in different fields and cite.

Answer:

To effectively improve the GPR image resolution, Zhang et al. combines the WT and F-K filtering [31]. Samuel et al. marked a comparative analysis of a set of machine learning models, the results shown that the best combination for predicting the streamflow into the Sobradinho Reservoir was the bootstrap, WT and neural network [32].

Comment: It will be useful if the authors could bring up a flow chart to guide the readers about step-by-step processes of making forecasts or projection which is the aim of this study.

Answer: we have made flow chart following your suggestions，given below:

Comment: It will be good if the authors take some serious time for a careful proofreading. There are some errors which automated word processor grammar and corrections can detect.

Answer: we have made changes following your suggesstion

\#

Comment: Fig. 10 outcome needs to be discussed well, being the key conclusion.

Answer: we have made changes following your suggesstion, given below

Finally, Fig.11 compares in box plot the observed and predicted PI values dispersion and indicates the median (*M*). The performance of the three models is very similar, in phase one, with median values very close *(M_observed_=1.612, M_TCN_=1.602, M_GRU_=1.648, M_LSTM_=1.719,M_SVM_=1.569,M_RF_=1.854)*, but the box’s shape of TCN is more close to the box’s shape of observed, it also mean that the whole prediction result of TCN is better than others’. In both phase two and phase three, the performance of LSTM, GRU, SVM and RF obvious gets worse as prediction interval grows, while TCN has not change much. Overall, TCN substantially outperform generic recurrent architectures such as LSTM and GRU and traditional model such as SVM and RF.

Comment: Once the pollutants are out in the atmosphere, there is also important consequences for the society. There are plenty of literature on this topic as well. But, I referred to one of them below.

A novel approach for the characterization of transport and optical properties of aerosol particles near sources–Part I and Part II

Answer：we have made changes following your suggesstion, given below：

For example, the organic matter present in excreta and/or litter resulting in pollutants production like Co2, ammonia, and TSP, will not only impair geese and staff health, there are also important consequences for society once the pollutants are out in the atmosphere [3,4].

3.	Valdebenito B ÁM, Pal S, Behrendt A, Wulfmeyer V, Lammel G. A novel approach for the characterisation of transport and optical properties of aerosol particles near sources - Part II: Microphysics-chemistry-transport model development and application. Atmos Environ. 2011. doi:10.1016/j.atmosenv.2010.09.004

4.	Behrendt A, Pal S, Wulfmeyer V, Valdebenito B. ÁM, Lammel G. A novel approach for the characterization of transport and optical properties of aerosol particles near sources - Part I: Measurement of particle backscatter coefficient maps with a scanning UV lidar. Atmos Environ. 2011. doi:10.1016/j.atmosenv.2011.02.061
