# Supplementary material for: Pollution index of waterfowl farm assessment and prediction based on temporal convoluted network
Source: PLoS One. 2021 Jul 23;16(7):e0254179. doi: 10.1371/journal.pone.0254179 (PMC8301615; doi:10.1371/journal.pone.0254179)
Supplement: Attachment — Submitted filename: Response to Reviewers.docx [file pone.0254179.s003.docx]

Response to Journal:

Reviewer #1: While I commend the authors for working diligently on the suggested comments by both reviewers, the manuscript is still not in a publishable state. I still maintain that the work is interesting and prudent, but the authors have not fully addressed some of the major comments brought up previously. First being the English is still not at a level of an academic paper and really needs major edits which I cannot put forth the time to do.

Answer: Thank you very much for your prestigious comments. This paper was edited for proper English language, grammar, punctuation, spelling, and overall style by one or more of the highly qualified native English speaking editors at AJE.

Second, the paper is still very methods focus despite the fact that both reviewers pointed out that there needs to be more on the "why" do the study. What's the motivation? How can it help farmers. The authors added a few lines here and there but it is still not compelling.

Answer: We have added more references to this study. It includes why we need to monitor and control the poultry house environment, what methods have been used to do that thing by others, how can they help the farmer and why we need the new method.

Labor shortages and increasing biosecurity practices will make it more difficult for producers to monitor and manage the production, health, and welfare status of all of their birds. Employing modern poultry management technology is necessary to increase production [6]. An example of how modern management technology can be used to monitor and control the poultry house environment is exemplified by humidity regulation via ventilation rate changes mediated by relative humidity sensors, as relative humidity is one of the more important environmental aspects of a poultry house [7]. In addition, more advanced systems are being researched. Bustamante et al. used a multisensor system to effectively assess barn ventilation system function by tracking temperature, air velocity and differential pressure in broiler houses [8]. Hanif et al. proposed an internet of things technology-based protection and monitoring of the environment of a poultry house to monitor the environment-related parameters such as air temperature, air humidity, CO_2_ level of concentration and ammonia concentration., which has been implemented successfully, leading to a safe environment and profit for the poultry industry [9]. The techniques mentioned above are both solutions for real-time environmental monitoring and control; however, relying on hardware monitoring in real time cannot capture the trend of environmental changes [10], and it is easy to miss the best time for adjustment, which leads to waterfowl health damage and property loss.

Cultivation environment forecasting has been studied for many years and has made some achievements in aquaculture and livestock breeding [11–14]. The technique estimates or predicts the future changes in target variables that cannot be obtained directly. For example, Jackman et al. generated a prediction model by using sensor inputs of relative humidity, carbon dioxide, temperature, and ammonia for environmental parameter and crop yield prediction [15]. In waterfowl production, a system such as this would allow for actions to be taken sooner by farmers if the environment is projected to be bad. However, few studies have applied prediction in waterfowl breeding. Therefore, it is necessary to apply environmental prediction technology to waterfowl production to fill this gap.

1. While the first table highlights the data chosen for the PI. The text does not go into much detail about why these were chosen, and what are the acceptable limits. I still do not see a mention of other similar studies on waterfowl or poultry in the introduction.

Answer: We have added more references to detail describe why data were chosen, and what are the acceptable limits.

Labor shortages and increasing biosecurity practices will make it more difficult for producers to monitor and manage the production, health, and welfare status of all of their birds. Employing modern poultry management technology is necessary to increase production [6]. An example of how modern management technology can be used to monitor and control the poultry house environment is exemplified by humidity regulation via ventilation rate changes mediated by relative humidity sensors, as relative humidity is one of the more important environmental aspects of a poultry house [7]. In addition, more advanced systems are being researched. Bustamante et al. used a multisensor system to effectively assess barn ventilation system function by tracking temperature, air velocity and differential pressure in broiler houses [8]. Hanif et al. proposed an internet of things technology-based protection and monitoring of the environment of a poultry house to monitor the environment-related parameters such as air temperature, air humidity, CO_2_ level of concentration and ammonia concentration., which has been implemented successfully, leading to a safe environment and profit for the poultry industry [9]. The techniques mentioned above are both solutions for real-time environmental monitoring and control; however, relying on hardware monitoring in real time cannot capture the trend of environmental changes [10], and it is easy to miss the best time for adjustment, which leads to waterfowl health damage and property loss.

Cultivation environment forecasting has been studied for many years and has made some achievements in aquaculture and livestock breeding [11–14]. The technique estimates or predicts the future changes in target variables that cannot be obtained directly. For example, Jackman et al. generated a prediction model by using sensor inputs of relative humidity, carbon dioxide, temperature, and ammonia for environmental parameter and crop yield prediction [15]. In waterfowl production, a system such as this would allow for actions to be taken sooner by farmers if the environment is projected to be bad. However, few studies have applied prediction in waterfowl breeding. Therefore, it is necessary to apply environmental prediction technology to waterfowl production to fill this gap.

According to the importance of the environment and expert research, we selected 5 environmental factors, as shown in Table 1. Among them, ammonia is a toxic gas and the greatest concern of environmental pollution in waterfowl production, adversely affecting the ecosystem, environment, and health of birds and people. Less than 10 ppm is the ideal limit [42]. Relative humidity can impact bird health, and high relative humidity may worsen broiler geese performance; the ideal value is 70% [43]. Heat stress is a major concern in waterfowl production; high and low temperatures will reduce the growth performance and survivability of waterfowl, and temperatures less than 27°C and more than 10°C are ideal [44]. TSP is from the birds themselves as well as from the feed, litter, and building materials and may serve as a pathogen disseminator and bring about lung damage; less than 10 mg/m^3^ is the ideal limit [42]. Additionally, the levels of relative humidity, ammonia, carbon dioxide, temperature and TSP are known to be correlated with each other [6]. These factors are important for waterfowl production and can be monitored and optimized.

2. There is now way too much detail in the methods. I'm not sure you need every detail and plot for each AI technique in your paper unless this is the first time they are introduced. You should provide a brief summary of each and then if you want to include this detail move to SI.

Answer: We have simplify introduction of some methods.

**Wavelet transform**

Continuous wavelet transforms for a given waterfowl environment signal *s(k)* can be described as follows:

 (3)

where denotes the complex conjugate, is the wavelet function, *a* is the time scale dilation and *b* is the time translation. By controlling the values of parameters *a* and *b*, signal time-frequency positioning can be achieved. The wavelet transform can decompose signals into multiple resolutions. However, the symbols in realization communication are discrete data, so the continuous wavelet transform needs to be discretized. Suppose, when:

 (4)

The discrete waterfowl environment signal s(n) can be transformed as:

 (5)

The Mallat algorithm can quickly calculate the orthogonal wavelet transform coefficients and realize signal decomposition and reconstruction [47]. The approximation coefficients and detail coefficients can be recurrently related by the Mallat algorithm as follows:

 (6)

 (7)

where *h_0_* and *h_1_* are the high-pass filter and low-pass filter, respectively. The reconstruction is the inverse process of decomposition:

 (8)

~~The wavelet transform can decompose noise signals into different signal channels at various levels of resolution. In general, the useful signals are distributed in the high-level resolution channel, and the noise signal is likely to be distributed in the lower-level channel. Therefore, the noise of the original data can be reduced as much as possible based on a reasonable threshold. Therefore, the wavelet denoised algorithm can be described as follows:~~

(1) ~~Perform wavelet transform on the original signal~~ *~~s(k)~~* ~~to obtain wavelet coefficients~~ ~~of various scales.~~
(2) ~~Determine the threshold and perform threshold denoising on different wavelet coefficients to obtain estimated wavelet coefficients.~~
(3) ~~Conduct wavelet reconstruction by equation 11 to obtain the denoise signal~~~~.~~

**Support vector machine**

SVM has good generalization ability in solving nonlinear, small sample, and high-dimensional pattern recognition, and the optimal solution obtained is global, which solves the local optimal problem that cannot be avoided in other algorithms. The prediction process of the SVM includes support vector determination, kernel function selection, kernel parameter determination, and solution. ~~The mathematical expression as follow:~~

 ~~(9)~~

~~Where~~ *~~SVS~~* ~~is the number of support vectors,~~ *~~α~~_~~i~~_* ~~is the Lagrangian coefficient of each training sample,~~ *~~y~~_~~i~~_* ~~(-1 or 1) is the vector label,~~ *~~K (x~~_~~i~~_~~, x)~~* ~~is the selected kernel function, and~~ *~~b~~_~~0~~_* ~~is the bias. The kernel function replaces the linear quantity in the traditional linear equation and maps the data to high-dimensional space processing. After selected the kernel function, the samples are trained to establish the SVM model and the prediction result~~ *~~Nt~~* ~~is finally obtained.~~

**Long short-term memory neural network**

The LSTM neural network is a special kind of recurrent neural network. It was first proposed by Ho-chreiter and Schmidhuber [49]. Its appearance effectively solved the gradient explosion problem of traditional recurrent neural networks. At the same time, the LSTM neural network has long-term memory to handle long-term sequence data. ~~The LSTM cell structure consists of forget gate, input gate, output gate and cell state. LSTM structure is shown in Fig 2 At the time~~ *~~t~~*~~, there are three input parameters of the LSTM network: input value~~ *~~x~~_~~t,~~_* ~~at time~~ *~~t~~*~~, output value~~ *~~h~~_~~t-1~~_* ~~and cell state~~ *~~C~~_~~t-1~~_* ~~at~~ *~~t-1~~*~~. There are two output parameters of the LSTM network: output value~~ *~~h~~_~~t~~_* ~~and cell state~~ *~~C~~_~~t~~_* ~~at time~~ *~~t.~~* ~~Through the activation function~~ *~~σ~~* ~~LSTM realizes the control of the three gates so as to realize the retention and forgetting of historical information.~~

**~~Fig.2. The structure of LSTM~~**

**Gated recurrent unit**

A gated recurrent unit (GRU) is a type of recurrent neural network (RNN). Similar to a long short-term memory neural network (LSTM), it is also proposed to solve long-term and gradient backpropagation problems. LSTM and GRU have similar performances, but compared with LSTM, GRU is computationally cheaper. ~~The structure of GRU is shown in Fig 3 GRU can be descried as follows:~~

~~Firstly, two output gateds,~~ *~~r~~* ~~(reset gated) and~~ *~~z~~* ~~(update gated), were obtained by the input~~ *~~X(t)~~* ~~at~~ *~~t~~* ~~time and the output~~ *~~h(t − 1)~~* ~~at~~ *~~t − 1~~* ~~time.~~

 ~~(10)~~

 ~~(11)~~

~~Where~~~~CJ is a sigmoid function. Parameter~~ *~~h'~~* ~~selective re- members~~ *~~X(t)~~*~~. Trough the reset gated~~ *~~r~~* ~~we can obtain~~ *~~h’~~* ~~as follows:~~

 ~~(12)~~

~~Finally, the output~~ *~~y(t)~~* ~~and hidden state~~ *~~h(t)~~* ~~at t time can be calculated as follows:~~

 ~~(13)~~

 ~~(14)~~

~~Where [ ] denoted the connection of two vectors and * denoted the product of matrices.~~

**~~Fig.3. The structure of GRU~~**

3. Conclusions and future research again focus way too much on methods and not on the "why".

Answer: We have modified the conclusions.

To further promote waterfowl house environment monitoring and controlling technology, reduce labor and increase the production effect, this study analyses the shortcomings of existing methods and introduces a new way to guide waterfowl house environment management by learning from other fields.

The new method investigates the application of denoised WT and the performance of three neural network models and two mechanical learning models in predicting the pollution index of the waterfowl house environment using environmental quality parameters at different intervals. The results indicate that the TCN model has the best performance in predicting PI. The GRU model has similar performance but lower performance when the prediction time interval changed, and the LSTM model performed the worst among the three models, although it still provided fairly accurate PI predictions.

The models presented in this paper, in particular the TCN model, could provide accurate and long-interval PI predictions of waterfowl house environments and monitor them in real time. The simulation results show that this method can be applied in waterfowl house environment prediction. The future trend of the environment can be estimated and predicted compared with traditional real-time monitoring technology, which can allow better waterfowl house environment management practices, better culture plan design, and, in general, contribute to a more sustainable waterfowl house management approach.

The environmental parameters selected in the present study may also pose a limitation because of the lack of equipment. Future work may include the use of more environmental parameters to evaluate PI and apply the model in more waterfowl breeding sites to improve the production effect and further verify the model.
