# Supplementary material for: Pollution index of waterfowl farm assessment and prediction based on temporal convoluted network
Source: PLoS One. 2021 Jul 23;16(7):e0254179. doi: 10.1371/journal.pone.0254179 (PMC8301615; doi:10.1371/journal.pone.0254179)
Supplement: Attachment — Submitted filename: Response to Reviewers.docx [file pone.0254179.s005.docx]

Response to Journal:

Journal Requirements:

Please review your reference list to ensure that it is complete and correct. If you have cited papers that have been retracted, please include the rationale for doing so in the manuscript text, or remove these references and replace them with relevant current references. Any changes to the reference list should be mentioned in the rebuttal letter that accompanies your revised manuscript. If you need to cite a retracted article, indicate the article’s retracted status in the References list and also include a citation and full reference for the retraction notice.

Answer: We have removed these references :1, 7, 10, 13, 14, 49 and replaced them with other relevant current references.

**References**

1.	Qian Y, Song K, Hu T, Ying T. Environmental status of livestock and poultry sectors in China under current transformation stage. Sci Total Environ. 2018. doi:10.1016/j.scitotenv.2017.12.045

~~1.	CSB CSB. Statistical Communique of the People’s Republic of China on the 2019 National Economic and Social Development. 2020.~~

2.	Valdebenito B ÁM, Pal S, Behrendt A, Wulfmeyer V, Lammel G. A novel approach for the characterisation of transport and optical properties of aerosol particles near sources - Part II: Microphysics-chemistry-transport model development and application. Atmos Environ. 2011. doi:10.1016/j.atmosenv.2010.09.004

3.	Behrendt A, Pal S, Wulfmeyer V, Valdebenito B. ÁM, Lammel G. A novel approach for the characterization of transport and optical properties of aerosol particles near sources - Part I: Measurement of particle backscatter coefficient maps with a scanning UV lidar. Atmos Environ. 2011. doi:10.1016/j.atmosenv.2011.02.061

4.	Pereira WF, Fonseca L da S, Putti FF, Góes BC, Naves L de P. Environmental monitoring in a poultry farm using an instrument developed with the internet of things concept. Comput Electron Agric. 2020. doi:10.1016/j.compag.2020.105257

5.	Astill J, Dara RA, Fraser EDG, Roberts B, Sharif S. Smart poultry management: Smart sensors, big data, and the internet of things. Computers and Electronics in Agriculture. 2020. doi:10.1016/j.compag.2020.105291

6.	Bustamante E, Guijarro E, García-Diego FJ, Balasch S, Hospitaler A, Torres AG. Multisensor system for isotemporal measurements to assess indoor climatic conditions in poultry farms. Sensors (Switzerland). 2012. doi:10.3390/s120505752

7.	Lashari MH, Memon AA, Shah SAA, Nenwani K, Shafqat F. IoT Based poultry environment monitoring system. Proceedings - 2018 IEEE International Conference on Internet of Things and Intelligence System, IOTAIS 2018. 2019. doi:10.1109/IOTAIS.2018.8600837

~~7.	Conley S. Poultry Barn Meteorology Managing air quality through humidity control. 2012. Available: https://www.canadianpoultrymag.com/%0Aproduction/bird-management/poultry-barn-meteorology-12641~~

8.	Wei Y, Jiao Y, An D, Li D, Li W, Wei Q. Review of dissolved oxygen detection technology: From laboratory analysis to online intelligent detection. Sensors (Switzerland). 2019. doi:10.3390/s19183995

9.	Liu S, Xu L, Jiang Y, Li D, Chen Y, Li Z. A hybrid WA-CPSO-LSSVR model for dissolved oxygen content prediction in crab culture. Eng Appl Artif Intell. 2014;29: 114–124. doi:10.1016/j.engappai.2013.09.019

10.	Li C, Li Z, Wu J, Zhu L, Yue J. A hybrid model for dissolved oxygen prediction in aquaculture based on multi-scale features. Inf Process Agric. 2018. doi:10.1016/j.inpa.2017.11.002

~~10.	Li D, Yang H. State-of-the-art Review for Internet of Things in Agriculture. Nongye Jixie Xuebao/Transactions of the Chinese Society for Agricultural Machinery. 2018. doi:10.6041/j.issn.1000-1298.2018.01.001~~

11.	Jackman P, Ward S, Brennan L, Corkery G, McCarthy U. Application of wireless technologies to forward predict crop yields in the poultry production chain. Agric Eng Int CIGR J. 2015.

12.	Asadollah SBHS, Sharafati A, Motta D, Yaseen ZM. River water quality index prediction and uncertainty analysis: A comparative study of machine learning models. J Environ Chem Eng. 2020. doi:10.1016/j.jece.2020.104599

~~13.	Yang L, Liu C, Guo Y, Deng H, Li D, Duan Q. Prediction of Ammonia Concentration in Fattening Piggery Based on EMD-LSTM. Nongye Jixie Xuebao/Transactions Chinese Soc Agric Mach. 2019. doi:10.6041/j.issn.1000-1298.2019.S0.054~~

~~14.	Liu CH, Yang L, Deng H, Guo YC, Li DL, Duan QL. Prediction of ammonia concentration in piggery based on ARIMA and BP neural network. Zhongguo Huanjing Kexue/China Environ Sci. 2019.~~

13.	Odiete WE. Novel metric for managing the protection of humanity and the environment against pollution and its adverse effects. Heliyon. 2020;6: e05555. doi:https://doi.org/10.1016/j.heliyon.2020.e05555

14.	Martinus Y, Astono W, Hendrawan D. Water quality study of Sunter River in Jakarta, Indonesia. IOP Conference Series: Earth and Environmental Science. 2018. doi:10.1088/1755-1315/106/1/012022

15.	Rubio-Arias H, Contreras-Caraveo M, Quintana RM, Saucedo-Teran RA, Pinales-Munguia A. An overall water quality index (WQI) for a man-made aquatic reservoir in Mexico. Int J Environ Res Public Health. 2012. doi:10.3390/ijerph9051687

16.	Suriadikusumah A, Mulyani O, Sudirja R, Sofyan ET, Maulana MHR, Mulyono A. Analysis of the water quality at Cipeusing river, Indonesia using the pollution index method. Acta Ecol Sin. 2020. doi:10.1016/j.chnaes.2020.08.001

17.	Leong WC, Kelani RO, Ahmad Z. Prediction of air pollution index (API) using support vector machine (SVM). J Environ Chem Eng. 2020. doi:10.1016/j.jece.2019.103208

18.	Thach TQ, Tsang H, Cao P, Ho LM. A novel method to construct an air quality index based on air pollution profiles. Int J Hyg Environ Health. 2018. doi:10.1016/j.ijheh.2017.09.012

19.	Khanoranga, Khalid S. Phytomonitoring of air pollution around brick kilns in Balochistan province Pakistan through air pollution index and metal accumulation index. J Clean Prod. 2019. doi:10.1016/j.jclepro.2019.05.050

20.	Yang H, Wang F, Yu J, Huang K, Zhang H, Fu Z. An improved weighted index for the assessment of heavy metal pollution in soils in Zhejiang, China. Environ Res. 2021. doi:10.1016/j.envres.2020.110246

21.	Jorfi S, Maleki R, Jaafarzadeh N, Ahmadi M. Pollution load index for heavy metals in Mian-Ab plain soil, Khuzestan, Iran. Data Br. 2017. doi:10.1016/j.dib.2017.10.017

22.	Ramsey DSL, O’Brien DJ, Smith RW, Cosgrove MK, Schmitt SM, Rudolph BA. Management of on-farm risk to livestock from bovine tuberculosis in Michigan, USA, white-tailed deer: Predictions from a spatially-explicit stochastic model. Prev Vet Med. 2016. doi:10.1016/j.prevetmed.2016.09.022

23.	Choi HL, Sudiarto SIA, Renggaman A. Prediction of livestock manure and mixture higher heating value based on fundamental analysis. Fuel. 2014. doi:10.1016/j.fuel.2013.08.064

24.	Kargar K, Samadianfard S, Parsa J, Nabipour N, Shamshirband S, Mosavi A, et al. Estimating longitudinal dispersion coefficient in natural streams using empirical models and machine learning algorithms. Eng Appl Comput Fluid Mech. 2020. doi:10.1080/19942060.2020.1712260

25.	Alizadeh MJ, Kavianpour MR, Danesh M, Adolf J, Shamshirband S, Chau KW. Effect of river flow on the quality of estuarine and coastal waters using machine learning models. Eng Appl Comput Fluid Mech. 2018. doi:10.1080/19942060.2018.1528480

26.	Seng D, Zhang Q, Zhang X, Chen G, Chen X. Spatiotemporal prediction of air quality based on LSTM neural network. Alexandria Eng J. 2021;60: 2021–2032. doi:https://doi.org/10.1016/j.aej.2020.12.009

27.	Csábrági A, Molnár S, Tanos P, Kovács J. Application of artificial neural networks to the forecasting of dissolved oxygen content in the Hungarian section of the river Danube. Ecol Eng. 2017;100: 63–72. doi:10.1016/j.ecoleng.2016.12.027

28.	Ranković V, Radulović J, Radojević I, Ostojić A, Čomić L. Neural network modeling of dissolved oxygen in the Gruža reservoir, Serbia. Ecol Modell. 2010;221: 1239–1244. doi:10.1016/j.ecolmodel.2009.12.023

29.	Sun M, Hassan SG, Li D. Models for estimating feed intake in aquaculture: A review. Comput Electron Agric. 2016;127: 425–438. doi:10.1016/j.compag.2016.06.024

30.	Peng Q, Liu Z-W. Short-Term Residential Load Forecasting Based on Smart Meter Data Using Temporal Convolutional Networks. 2020 39th Chinese Control Conference (CCC). 2020. pp. 5423–5428. doi:10.23919/CCC50068.2020.9188453

31.	Bai S, Kolter JZ, Koltun V. An empirical evaluation of generic convolutional and recurrent networks for sequence modeling. arXiv. 2018.

32.	Bavkar S, Iyer B, Deosarkar S. Detection of Alcoholics using EEG and EMD Domain Statistical Features. 2020 International Conference on Emerging Smart Computing and Informatics, ESCI 2020. 2020. pp. 153–157. doi:10.1109/ESCI48226.2020.9167564

33.	Yan X, Boudrias MH, Mitsis GD. Artifact Removal in tACS-EEG Recordings: A Combined Methodology Based on the Empirical Wavelet Transform. Proceedings of the Annual International Conference of the IEEE Engineering in Medicine and Biology Society, EMBS. 2020. pp. 944–947. doi:10.1109/EMBC44109.2020.9176488

34.	You SD, Hung MJ. Reducing Dimensionality of Spectro-Temporal Data by Independent Component Analysis. 2020 2nd International Conference on Computer Communication and the Internet, ICCCI 2020. 2020. pp. 93–97. doi:10.1109/ICCCI49374.2020.9145984

35.	Kumar A, Tomar H, Mehla VK, Komaragiri R, Kumar M. Stationary wavelet transform based ECG signal denoising method. ISA Trans. 2020. doi:https://doi.org/10.1016/j.isatra.2020.12.029

36.	Zhang L, Ling T, Yu B, Huang F, Zhang S. Intensive Interferences Processing for GPR signal based on the Wavelet Transform and F-K Filtering. J Appl Geophys. 2021. doi:10.1016/j.jappgeo.2021.104273

37.	Saraiva SV, Carvalho F de O, Santos CAG, Barreto LC, Freire PK de MM. Daily streamflow forecasting in Sobradinho Reservoir using machine learning models coupled with wavelet transform and bootstrapping. Appl Soft Comput. 2021. doi:10.1016/j.asoc.2021.107081

38.	David B, Moe RO, Michel V, Lund V, Mejdell C. Air Quality in Alternative Housing Systems May Have an Impact on Laying Hen Welfare. Part I—Dust. 2015; 495–511. doi:10.3390/ani5030368

39.	Najafi P, Zulkifli I, Amat Jajuli N, Farjam AS, Ramiah SK, Amir AA, et al. Environmental temperature and stocking density effects on acute phase proteins, heat shock protein 70, circulating corticosterone and performance in broiler chickens. Int J Biometeorol. 2015. doi:10.1007/s00484-015-0964-3

40.	Gebregeziabhear E, Ameha N. The effect of stress on productivity of animals: a review. J Biol Agric Healthc. 2015;5: 165–172.

41.	David B, Mejdell C, Michel V, Lund V, Moe RO. Air quality in alternative housing systems may have an impact on laying hen welfare. Part II-ammonia. Animals. 2015. doi:10.3390/ani5030389

42.	K.Othman J, R. Mahmood J, Y. A. Al- Emarah G. Design and Implementation of Smart Relay based Remote Monitoring and Controlling of Ammonia in Poultry Houses. Int J Comput Appl. 2014. doi:10.5120/18093-9149

43.	Mallat SG. A Theory for Multiresolution Signal Decomposition: The Wavelet Representation. IEEE Trans Pattern Anal Mach Intell. 1989. doi:10.1109/34.192463

44.	Breiman L. Random forests. Mach Learn. 2001. doi:10.1023/A:1010933404324

45.	Gers FA, Schmidhuber J, Cummins F. Learning to forget: Continual prediction with LSTM. Neural Comput. 2000. doi:10.1162/089976600300015015

46.	Zhang Z, Chen H, Huang Y, Lee WJ. Quantile Huber Function Guided TCN for Short-Term Consumer-Side Probabilistic Load Forecasting. 2020 IEEE/IAS Industrial and Commercial Power System Asia, I and CPS Asia 2020. 2020. doi:10.1109/ICPSAsia48933.2020.9208545

47.	Long J, Shelhamer E, Darrell T. Fully convolutional networks for semantic segmentation. Proceedings of the IEEE Computer Society Conference on Computer Vision and Pattern Recognition. 2015. doi:10.1109/CVPR.2015.7298965

48.	He K, Zhang X, Ren S, Sun J. Deep residual learning for image recognition. Proceedings of the IEEE Computer Society Conference on Computer Vision and Pattern Recognition. 2016. doi:10.1109/CVPR.2016.90

~~49.	Sepp H, J?urgen S. 1997Hochreiter_LSTM. Neural Comput. 1997.~~

Reviewer #1: The authors have done a much better job addressing remaining concerns, especially with language. I made suggested grammatical changes in the attached document. I have some other minor things to address below, but then should be suitable for publication.

Comment:1. The order of the methods and results seems off. For example there is a wavelet transform section and then further section on wavelet transform starting on 270. Then you also have results of denoising in methods not results. I think rearranging for a better flow is needed.

Answer: Thank you very much for your prestigious comments we have modified the manuscript and made changes.

**Wavelet transform**

Continuous WTs for a given waterfowl environment signal *s(k)* can be described as follows:

$WTs\left(a,b\right)=\frac{1}{\sqrt{a}}\int_{\infty}^{-\infty} s\left(k\right)\overline{\left(\psi\left(\frac{t-b}{a}\right)\right)}dx$ (3)

where denotes the complex conjugate, $\psi\left(x\right)$ is the wavelet function, *a* is the time scale dilation and *b* is the time translation. By controlling the values of parameters *a* and *b*, signal time-frequency positioning can be achieved. The WT can decompose signals into multiple resolutions. However, the symbols in realization communication are discrete data, so the continuous WT needs to be discretized. Suppose $a=a_{0}^{j},b=kb_{0}a_{0}^{j},j,k\in Z$, when $a_{0}=2,b_{0}=1$:

$\psi_{j,k}\left(n\right)=2^{\frac{-j}{2}}\psi\left(2^{-j}n-k\right)$ (4)

The discrete waterfowl environment signal *s(n)* can be transformed as:

$D\left(j,k\right)=\sum_{k} s\left(n\right)\overline{\psi_{j,k}}\left(n\right)$ (5)

The Mallat algorithm can quickly calculate the orthogonal WT coefficients and realize signal decomposition and reconstruction [43]. The approximation coefficients $s_{j+1}\left(n\right)$and detail coefficients $d_{j+1}\left(n\right)$ can be recurrently related by the Mallat algorithm as follows:

$s_{j+1}\left(k\right)=\sum_{n} h_{0}\left(n-2k\right)s_{j}\left(n\right)$ (6)

$d_{j+1}\left(k\right)=\sum_{n} h_{1}\left(n-2k\right)s_{j}\left(n\right)$ (7)

where *h_0_* and *h_1_* are the high-pass filter and low-pass filter, respectively. The reconstruction is the inverse process of decomposition:

$s_{j}\left(k\right)=\sum_{n} s_{j+1}\left(n\right)h_{0}\left(k-2n\right)+\sum_{n} d_{j+1}\left(n\right)h_{1}\left(k-2n\right)$ (8)

Different environmental factors may contain different kinds of noise. To achieve the best effect of the denoising process and obtain quality data, we adopt four wavelet functions, Db4, Haar, Coif, and Sym10, to process each environmental dataset. The signal-noise ratio (SNR) and normalized cross correlation (NCC) were used to evaluate the denoised effect, which can be described as:

$SNR=\frac{I_{1}^{2}}{\left(I_{2}-I_{1}\right)^{2}}$ (9)

$NCC=\frac{\sum\left(I_{1}-mean\left(I_{1}\right)\right)\cdot\left(I_{2}-mean\left(I_{2}\right)\right)}{\sqrt{\sum\left(I_{1}-mean\left(I_{1}\right)\right)^{2}\times\sum\left(I_{2}-mean\left(I_{2}\right)\right)^{2}}}$ (10)

where *I_1_* is the denoised signal and *I_2_* is the original signal; theoretically, the larger the SNR and NCC are, the better the noise reduction effect.

……………………………….

**Results and discussion**

**Simulation results and discussion**

As shown in Table 2, wavelet function Db4 was suitable for ammonia data (SNR=9.4318, NCC=0.9485), Sym10 was suitable for temperature data (SNR=7.4635, NCC=0.9221), relative humidity data (SNR=4.0809, NCC=0.8630), and CO2 data (SNR=1.7785, NCC=0.7793), and Coif was suitable for TSP data (SNR=13.1443, NCC=0.9771). The best denoised results of each environmental dataset are shown in Fig. 6.

Table 2 Denoised performance of five parameters.

| Target | Performance criteria | Wavelet function | | | |
| --- | --- | --- | --- | --- | --- |
|  |  | Db4 | Haar | Coif | Sym10 |
| Ammonia | SNR/(dB) | 9.4318 | 8.8287 | 9.3769 | 9.3602 |
|  | NCC | 0.9485 | 0.9432 | 0.9484 | 0.9474 |
| Temperature | SNR/(dB) | 7.2491 | 6.5372 | 7.0205 | 7.4635 |
|  | NCC | 0.9192 | 0.9061 | 0.9149 | 0.9221 |
| Relative humidity | SNR/(dB) | 3.9422 | 3.6395 | 3.8920 | 4.0809 |
|  | NCC | 0.8600 | 0.8559 | 0.8596 | 0.8630 |
| CO_2_ | SNR/(dB) | 1.7064 | 1.3855 | 1.4991 | 1.7785 |
|  | NCC | 0.7764 | 0.7642 | 0.7689 | 0.7793 |
| TSP | SNR/(dB) | 12.8848 | 12.6578 | 13.1443 | 13.1142 |
|  | NCC | 0.9756 | 0.9745 | 0.9771 | 0.9769 |

(a) **Ammonia sequence.**

**(b) Temperature sequence.**

**(c) Relative humidity sequence.**

**(d) CO_2_ sequence.**

**(e) TSP sequence.**

**Fig. 6 Results of noise reduction of five parameters by WT.**

Comment:2. Acronyms need to be fixed. You should define acronyms the first time only and then use the acronym after. You have many in the manuscript so this needs to be addressed.

Answer: Following your suggestion, we have checked and modified the acronyms problems all over the paper.

Comment:3. Figures 6a-e are still hard to read. Maybe try removing the line on the original data so that the observed points can be seen overlain on denoised data?

Answer: Following your suggestion, we have modified the figures 6a-e.

(a) **Ammonia sequence.**

**(b) Temperature sequence.**

**(c) Relative humidity sequence.**

**(d) CO_2_ sequence.**

**(e) TSP sequence.**

**Fig. 6 Results of noise reduction of five parameters by WT.**
